# Complex Mixtures: Array PBPK Modeling of Jet Fuel Components

**DOI:** 10.3390/toxics11020187

**Published:** 2023-02-17

**Authors:** Teresa R. Sterner, Tammie R. Covington, David R. Mattie

**Affiliations:** 1 Henry M. Jackson Foundation for the Advancement of Military Medicine, Wright-Patterson Air Force Base, Dayton, OH 45433, USA; 2 Air Force Research Laboratory, 711HPW/RHBAF, Wright-Patterson Air Force Base, Dayton, OH 45433, USA

**Keywords:** PBPK, mixture, jet fuel

## Abstract

An array physiologically-based pharmacokinetic (PBPK) model represents a streamlined method to simultaneously quantify dosimetry of multiple compounds. To predict internal dosimetry of jet fuel components simultaneously, an array PBPK model was coded to simulate inhalation exposures to one or more selected compounds: toluene, ethylbenzene, xylenes, n-nonane, n-decane, and naphthalene. The model structure accounts for metabolism of compounds in the lung and liver, as well as kinetics of each compound in multiple tissues, including the cochlea and brain regions associated with auditory signaling (brainstem and temporal lobe). The model can accommodate either diffusion-limited or flow-limited kinetics (or a combination), allowing the same structure to be utilized for compounds with different characteristics. The resulting model satisfactorily simulated blood concentration and tissue dosimetry data from multiple published single chemical rat studies. The model was then utilized to predict tissue kinetics for the jet fuel hearing loss study (JTEH A, 25:1-14). The model was also used to predict rat kinetic comparisons between hypothetical exposures to JP-8 or a Virent Synthesized Aromatic Kerosene (SAK):JP-8 50:50 blend at the occupational exposure limit (200 mg/m^3^). The array model has proven useful for comparing potential tissue burdens resulting from complex mixture exposures.

## 1. Introduction

The jet fuel JP-8 is a kerosene-range petroleum fuel currently utilized for land-based operations by the U.S. Armed Forces [1] and NATO [2], and is the largest chemical exposure experienced by warfighters [3]. Globally, aircraft pilots, technicians and maintenance crews experience increased hearing loss, presumably from noise exposure. Swedish commercial aircraft technicians and mechanics [4], French military fighter, transport and helicopter pilots [5], and Thai helicopter pilots, aircrew, aircraft technicians and mechanics [6] have documented excessive hearing loss compared to reference populations. A preliminary epidemiology study of military workers found that JP-4 fuel exposure appeared to correlate with increased hearing loss, as compared to noise exposure alone [7]. These findings were corroborated by Prasher et al. [8] and Guest et al. [9], both of whom found that aircraft and fuel tank maintenance workers exposed to fuels, solvents and noise experienced an increase in hearing impairments when compared to populations in noise-alone environments. A literature review by Warner et al. [10] concluded that central auditory nervous system (CANS) evaluations should be mandatory for all military workers exposed to noise and solvents, including jet fuel.

Multiple rat studies support the role of JP-8 in enhancing noise-induced hearing loss (NIHL) [11,12,13]. A rat inhalation study by Guthrie et al. [14] indicated that JP-8 jet fuel exposure, with and without noise, resulted in minimal effects on peripheral auditory systems. However, this study revealed clear central auditory processing dysfunction (CAPD) from JP-8 exposure, both with and without co-exposure to regulatory safe levels of noise (85 dBA). Subsequent data analyses [15,16,17] indicated delayed neural transmission times, impaired neural adaptation, and degeneration of neural circuitry when fuel and noise are combined. Therefore, CAPD would be an expected outcome among military workers involved with aircraft.

JP-8 is composed of thousands of compounds, concentrations of which fluctuate depending on the source of the crude oil and refining variability [18]. Most of the JP-8 constituents incorporated into the array model are ototoxicants. The aromatics toluene, ethylbenzene, p-xylene are known to cause cochlear hair cell damage [19,20]. White spirit components, n-nonane and n-decane, primarily affect the central auditory pathway [21,22]. Although naphthalene is not a known ototoxicant, it comprises up to 1 percent of the JP-8 mixtures [23], is currently listed by the U.S. Environmental Protection Agency as a possible human carcinogen [24], and its metabolites can be used as biomarkers of jet fuel exposure [25,26].

**Figure 1:**
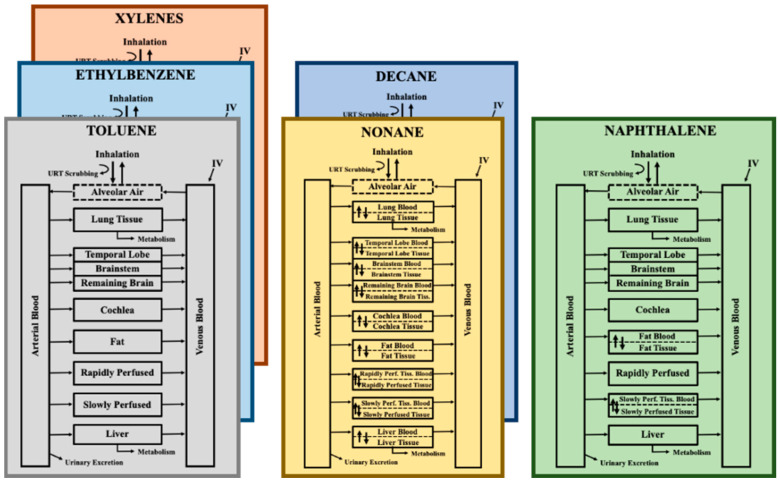
Hypothetical PBPK Model Schematic for a Traditionally Designed Six Compound JP-8 Mixtures Model. Each sub-model shows the compartments and processes for which code would be written and parameterized individually. Sub-models with the same configurations are stacked to further highlight the repetitiveness of traditional mixtures modeling. IV = intravenous; Perf. = perfused; Tiss. = Tissue; URT = upper respiratory tract.

Physiologically-based pharmacokinetic (PBPK) models are tools utilized to simulate absorption, distribution, metabolism, and excretion over different exposure scenarios and to extrapolate exposures between animals and humans for single compounds or mixtures [27]. Traditional mixtures models have been and are still coded in parallel with lines of code for each individual chemical in the mixture, and repeated for each tissue simulated (e.g., Martin et al. [28] and Ruiz et al. [29]). Increasing the number of chemicals rapidly inflates the lines of code required, resulting in long models and multiple opportunities for coding errors. The idea to develop a mixture model in an array format was conceived to remove the need to add an additional sub-model to the code each time the mixture was expanded. Figure 1 visually represents a hypothetical traditional mixtures model with sub-models for six JP-8 constituents.

The objective of this work was to develop an array PBPK mixtures model capable of describing the tissue dose of JP-8 components along the peripheral and central auditory pathways. The array would allow for easy addition of new compounds to the mixture, but also enable simulation of tissue concentrations of just one or two components if needed. The design of the model would also promote the comparison of predicted kinetics for different fuels or fuel mixtures for the purpose of exploring the potential outcome of switching to alternative fuels.

## 2. Materials and Methods

Development of the array model began by adapting a PBPK model for isopropanol and its metabolite, acetone (source model) [30], to an array format. The array model has the same structure (i.e., tissue compartments and kinetic descriptions) for all chemicals in the mixture. Code for each tissue is included only once, regardless of the number of components in the mixture. Physiological parameters that would not change based on the chemical (e.g., cardiac output or fraction of liver weight) were defined as scalar (single) values. Chemical-specific parameters (e.g., molecular weight) and physico-chemical properties (e.g., tissue:blood partition coefficients and metabolic constants) were defined as arrays (rows and columns of values). Values were assigned to the arrays based on the array (column) position assigned to the compound. Toluene, ethylbenzene, xylenes, nonane, decane and naphthalene were assigned column positions 1 through 6, respectively. Expansion of the model to additional compounds would simply require resetting the size of the arrays and adding script files accordingly. The array model code and script files, written for acslX (Aegis Technologies Group, Orlando, FL, USA), are found in the Supplemental Materials.

**Figure 2:**
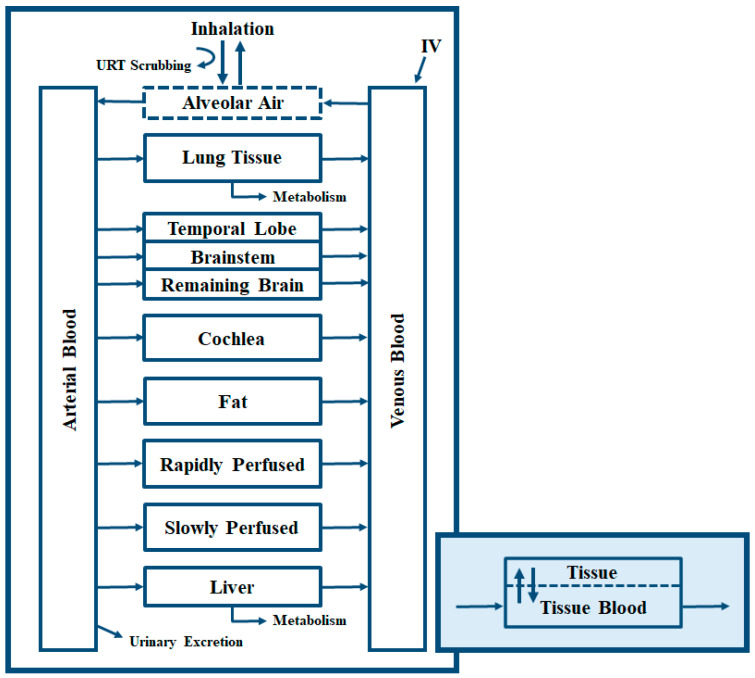
Physiologically-Based Pharmacokinetic Model Schematic. The general model schematic (white box) indicates the tissue compartments in the model structure. Each tissue in the general schematic is divided into the tissue itself and the tissue blood (blue box). This convention allows the model to simulate diffusion-limited chemicals as needed. IV = intravenous; URT = upper respiratory tract.

The array model was coded to simulate kinetics simultaneously in nine tissue compartments: brainstem, temporal lobe, remaining brain tissue, cochlea, lung, fat, liver, rapidly perfused tissues, and slowly perfused tissues (Figure 2). Dose routes coded into the model include intravenous (IV) and inhalation, with the latter coded as partitioning between alveolar air and blood. IV injection was modelled as a zero-order rate into venous blood, based upon the length of the injection time. Inhalation was simulated for a specified air concentration and a species-specific ventilation rate. Oral gavage and drinking water dosing equations were retained in the code from prior projects but were not used for this project. The upper respiratory tract (URT) scrubbing code from the source model [30] was modified to align with the scrubbing code from the decane model. Scrubbing describes the loss of chemical due to the filtering functions of the URT, thereby reducing the concentration available for absorption in the alveoli [31].

Chemical permeation into tissues is frequently described using the concentration in blood entering the tissue and simple tissue:blood partition coefficients (PCs), known as flow- or perfusion-limited distribution. To accommodate diffusion limitation, where chemical permeation is dependent on processes other than blood flow and partitioning [32], all tissue compartments were coded for diffusion-limited distribution (blue box in Figure 2). Diffusion limitation in any or all tissue compartments can be essentially nullified by setting the permeability coefficient to a very high number (e.g., 10,000) to closely approximate flow limitation. Naphthalene metabolism in the rat lung has been previously incorporated in PBPK models [33,34,35,36]; the array model was designed to include saturable metabolism in both the lung and liver compartments. For the other five compounds, lung metabolism is of minor significance and maximal rates of reaction for the lung were assigned a value of zero.

Rat physiological parameters were primarily based on the source model from which the array model was originally adapted [30] (Table 1). Lung parameters were obtained from literature sources, as described in Merrill et al. [36]. Brain and cochlea parameter sources were fully explained in Sterner et al. [37] and included ex vivo measurements. The model code calculated the volume (proportion of body weight in kg) assigned to a pair of rat cochlea using Equation (1), derived by linear regression from data reported in Sterner et al. [37].

Cochlea Pair Weight in kg = 7 × 10^−5^ (Body Weight in kg^0.2348^) (1)

Sources of chemical specific parameters for the array model (Table 2) have been documented in Sterner et al. [36,37,43]. PCs were obtained using literature, ex vivo vial equilibration measurements, and target tissue composition quantitative structure property relationship (QSPR) algorithms developed by Ruark et al. [44]. Target tissue composition (water, protein, neutral lipids, neutral phospholipids, and acidic phospholipids) was measured for the tissues of interest [37,45]. All xylene isomers were described with the same PCs in the array model. For nonane and decane, a white spirits study [46] provided total brain concentrations. Total brain concentrations (brain remainder, temporal lobe, and brainstem) were fitted to the published brain data. The fit PCs were assigned to the brain remainder, to represent average brain:blood values, and QSPR calculations were used to determine relative concentrations for the brainstem and temporal lobe (Table 2). Using these calculated PCs, the predicted brain concentrations were then re-simulated against the data to assure a good fit. QSPR algorithms [44] and tissue composition information developed for the project [45] allowed naphthalene parameterization [36], as this compound was modeled later, and will also permit the incorporation of additional JP-8 compounds in the future.

Metabolic and excretion parameters for each compound were obtained through literature (published models) and data fitting [36,37,43] (Table 3). Upper respiratory tract scrubbing was modified to reflect its use in the decane model [31]; a smaller scrubbing factor was fitted [37] to allow the model to simulate published nonane data. Values for the oral administration route were set to 0 and not included below. First order metabolism (kFC) was included from the source model [30] but set to 0 as saturable metabolism was used for the six compounds in the array model.

Following parameterization, each compound was simulated and compared to published kinetic data. Unreferenced parameters were set based on visual goodness of fit. Reasonable visually determined fits were obtained for individual compounds. The model was then utilized to predict first day target tissue concentration estimates following a select JP-8 exposure. Guthrie et al. [14] exposed Long-Evans rats (male, mean starting body weight 105 g) to 1000 mg/m^3^ JP-8 for 6 h per day, 5 days a week, for 4 weeks, without noise. This important exposure, repeated over 4 weeks, resulted in CAPD but not peripheral hearing changes.

The Guthrie et al. study [14] used a JP-8 fuel blend referred to as POSF 4658, which is maintained at the Air Force Research Laboratory Fuels and Energy Branch (AFRL/RQTF) located at Wright-Patterson Air Force Base, OH, USA. RQTF was formerly known as the Air Force Wright Aeronautical Laboratories fuel lab (AFWAL/POSF); fuels are still identified using POSF log book numbers. Predictions of the key components in this exposure scenario were based upon their percent by volume in POSF 4658 fuel, assuming standard temperature and pressure. The percentages were measured using tandem gas chromatography (GC × GC) analysis [18] and are shown in Table 4.

The model was then utilized to predict rat tissue concentrations at the JP-8 OEL (200 mg/m^3^ [48]), for both JP-8 and a 50:50 mixture of Virent Synthesized Aromatic Kerosene (SAK). Virent SAK (POSF 10326) is an alternative fuel derived from bio-based feed stocks using Virent’s BioForming^®^ process. This fuel is in the ASTM International process for approval. A jet engine test flight was successfully completed using Virent SAK in 2016 [49]. Percent weight values of the six key components in Virent SAK are also shown in Table 4, as well as the values for the 50:50 blend of the two fuels.

Finally, the model was assessed using a local sensitivity analysis. The simulation used for the process was a rat JP-8 exposure at the OEL [48] for 6 h, followed by a 6-h recovery. Dose metrics were the maximum concentration (Cmax) and area under the curve (AUC) for arterial blood, brainstem tissue, and liver tissue. Input parameters were varied by 1% and the resulting effects on the dose metrics were recorded. When a fractional tissue blood flow or tissue volume was adjusted by 1%, the remaining fractions were also adjusted within the code to maintain the total cardiac output or fraction of body weight represented, respectively. For chemical specific array parameters, values were varied individually for each chemical. Moreover, as chemical interactions are not currently coded in the model, output from varying a chemical specific parameter was only recorded for that specific chemical (i.e., only output for toluene was recorded when varying the toluene specific parameters) as there was no effect on the output for the other chemicals. Parameters not used (e.g., metabolic rates set to zero or permeability coefficients set to 10,000.0) were not included in the analysis. Normalized sensitivity coefficients were calculated as the fractional change in output divided by the fractional change in input using the central difference method and were ranked as having low (0.1–0.2), medium (>0.2 to 0.5), or high (>0.5) sensitivity [32].

## 3. Results

### 3.1. Model Usefulness and Validation

The array PBPK model allowed simulation of kinetic information for multiple components simultaneously using an identical model structure for each component. The model used species-specific physiological parameters and chemical-specific characteristics to simultaneously solve for tissue concentrations of each modeled compound. Changes to the model structure, such as addition of a tissue, required alteration of just one set of equations, as opposed to a special set of equations and parameter names for each chemical modeled, thus decreasing coding time and the likelihood of introduced error. Further, increasing the number of compounds was as simple as changing the array size and adding chemical-specific parameters in the script files, provided that the current model structure met the kinetic descriptions needed by the newly added compound. The model code and script files for running different scenarios are recorded in the Supplemental Materials.

**Figure 3:**
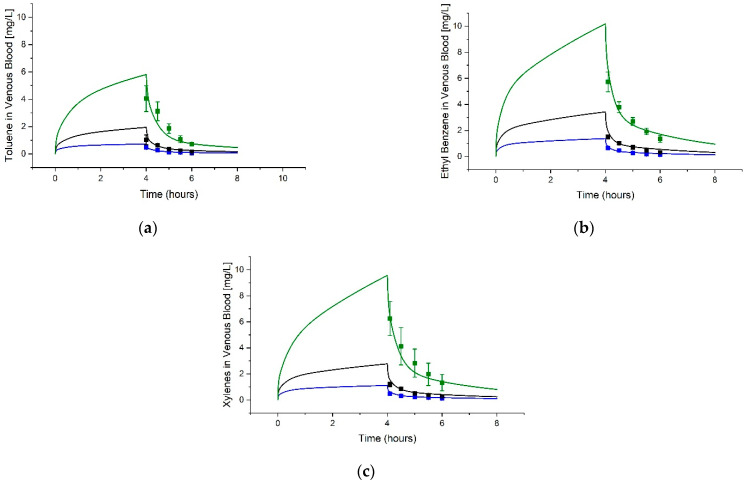
Example simulations of Toluene, Ethylbenzene, and Xylenes Venous Blood Concentrations. The graphs show model simulations (curves) and data (squares showing mean and standard deviation (STDEV)) digitized from Haddad et al. [47] for (**a**) toluene, (**b**) ethylbenzene, and (**c**) xylenes. Male Sprague-Dawley rats were exposed to 50 ppm (blue), 100 ppm (black), or 200 ppm (green) of each compound for 4 h.

**Figure 4:**
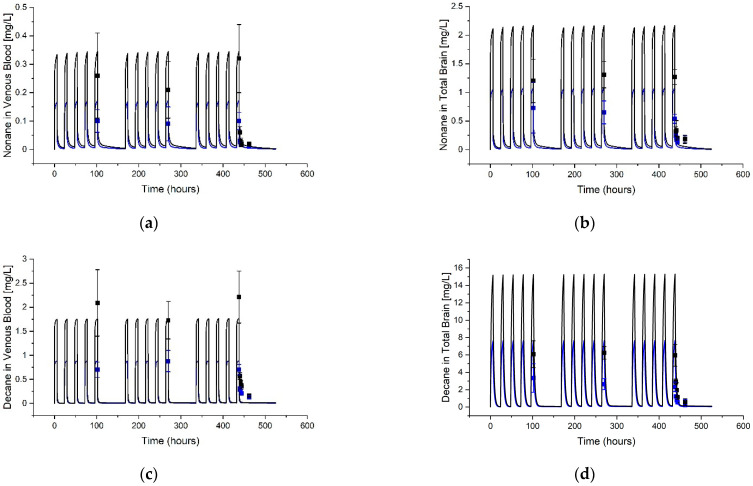
Example Simulations of Nonane and Decane Blood and Total Brain Concentrations. The graphs show model simulations (curves) and data (squares showing mean and STDEV) digitized from Lof et al. [46] for (**a**) nonane venous blood, (**b**) nonane total brain, (**c**) decane venous blood, and (**d**) decane total brain concentrations. Male Wistar rats were exposed to white spirits. Exposures were equivalent to 14.4 ppm (blue lines and squares) or 28.8 ppm (black lines and squares) nonane plus 106 ppm (blue lines and squares) or 212 ppm (black lines and squares) decane for 6 h daily, 5 days a week, for 3 weeks.

**Figure 5:**
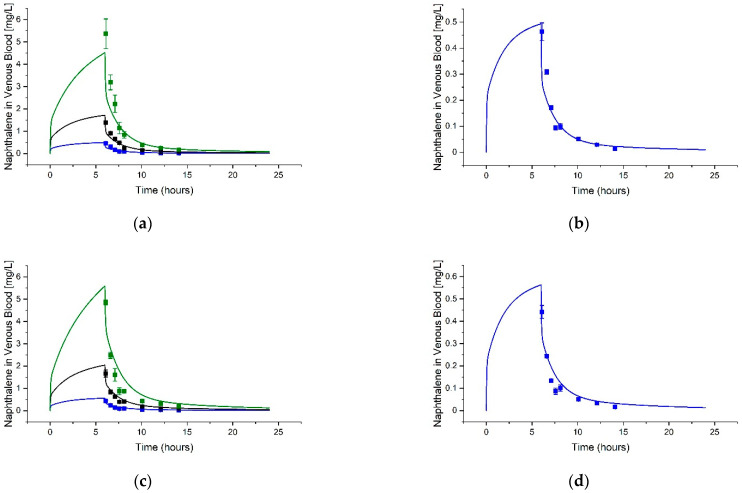
Example Simulations of Naphthalene Blood Concentrations. F344 rats were exposed to naphthalene for 6 h [53]. (**a**) Simulations depict male rat venous concentrations from 10 ppm (blue squares and lines), 30 ppm (black squares and lines), and 60 ppm (green squares and lines). (**b**) Closeup simulation for the male rat 10 ppm exposure only is shown. (**c**) Simulations depict female rat venous concentrations using the same color scheme. (**d**) Closeup simulation for the female rat 10 ppm exposure only is shown.

An example simulation against published data is shown for each jet fuel component in Figure 3, Figure 4 and Figure 5. The remainder of the data sets and simulations used during parameterization are found in the Supplemental Materials, including simulations based on data from additional sources not cited in the main article [50,51,52]. In general, visually acceptable fidelity was found between the simulations and published data following fitting of specific parameters, noted in Table 3.

### 3.2. Guthrie et al. (2014) [14] Tissue Predictions

**Figure 6:**
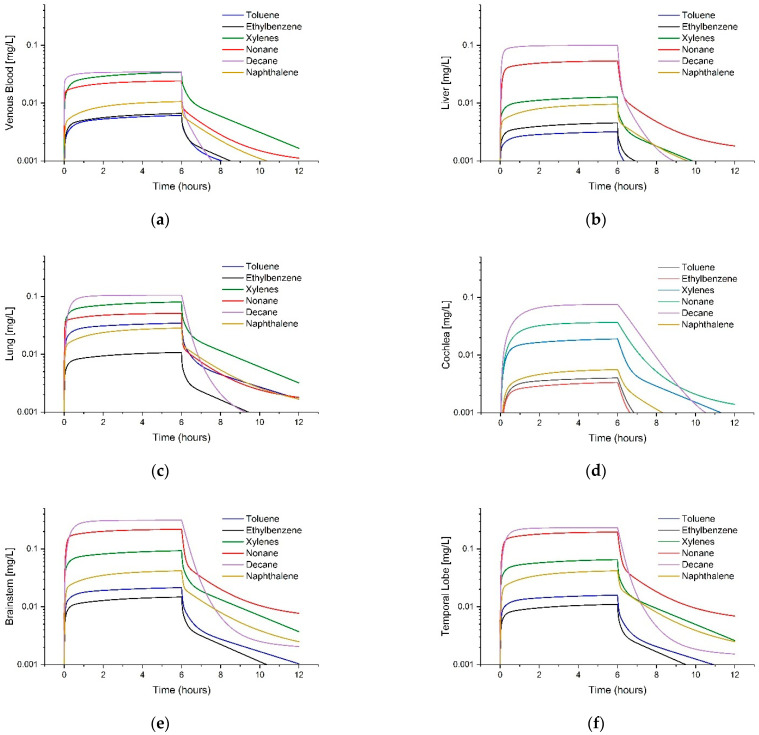
Predictions of Toluene, Ethylbenzene, Xylenes, Nonane, Decane, and Naphthalene Tissue Concentrations. The graphs show model predicted concentrations in (**a**) venous blood, (**b**) liver, (**c**) lung, (**d**) cochlea, (**e**) brainstem, and (**f**) temporal lobe following inhalation of 1000 mg/m^3^ of jet fuel (POSF 4658) by rats for 6 h [14].

Figure 6 contains tissue concentration predictions for the six volatile hydrocarbons following a 6-h exposure to 1000 mg/m^3^ JP-8 (POSF 4658) to mimic the Guthrie et al. [14] rat study. These predictions were equivalent to first day tissue profiles; in the study, rats were exposed for 6 h/day, 5 days/week for 4 weeks. The Y-axis on each graph is plotted on the same scale to facilitate tissue comparisons.

### 3.3. Comparison of Tissue Concentrations between Fuels

**Figure 7:**
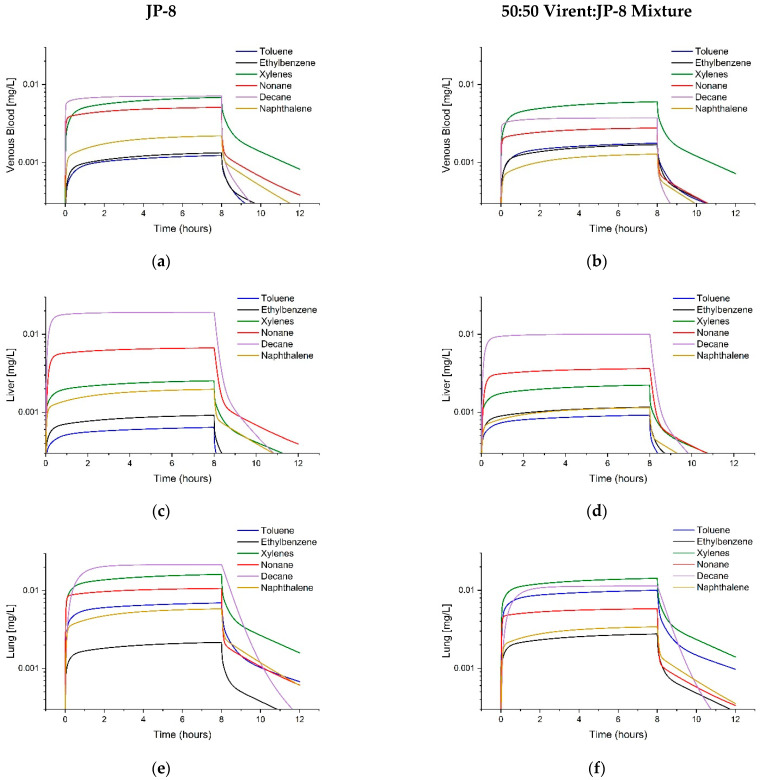
Comparison of Tissue Concentration Predictions in Rats for JP-8 and 50:50 Virent:JP-8 Mixture at the OEL. The graphs show paired tissue concentration predictions following exposure to 200 mg/m^3^ JP-8 (left graph) or a Virent:JP-8 mixture (right graph): (**a**,**b**) venous blood, (**c**,**d**) liver, and (**e**,**f**) lung.

**Figure 8:**
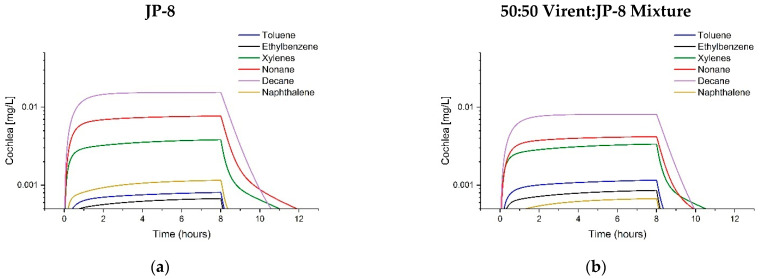

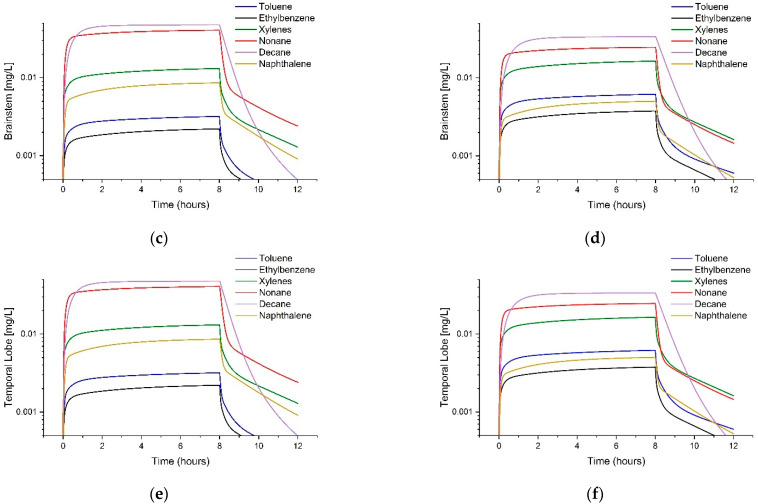
Comparison of Auditory Pathway Tissue Concentration Predictions in Rats for JP-8 and 50:50 Virent:JP-8 Mixture at the OEL. The graphs show paired tissue concentration predictions following exposure to 200 mg/m^3^ JP-8 (left graph) or a Virent:JP-8 mixture (right graph): (**a**,**b**) cochlea, (**c**,**d**) brainstem, and (**e**,**f**) temporal lobe.

To demonstrate the adaptability of the array model in predicting tissue concentrations for different fuels and the potential health effects of integrating alternative (non-petroleum) fuels into the supply chain, the model code was used to predict key fuel component concentrations in rat tissues for JP-8 and a 50:50 blend of Virent SAK:JP-8. These comparisons are shown in Figure 7 and Figure 8.

### 3.4. Sensitivity Analysis

Sensitivity analysis results are summarized by ranking in Table 5. A coefficient of 1 or −1 represents a 1% change in output to correspond to the 1% change in input. Negative coefficients represent a negative correlation between input and output or a decrease in the output parameter for an increase in the input parameter.

Apart from nonane and decane, all dose metrics (arterial blood, brainstem, and liver concentration AUCs and peak concentrations) were highly sensitive to ventilation rate (QPC), with particularly high sensitivity for naphthalene; an increase in ventilation rate typically results in a greater amount absorbed and a higher tissue dose. For the two normal alkanes (nonane and decane), the model was instead highly sensitive to the blood:air partition (PB) and the fraction scrubbed (Scrub), with a higher sensitivity to Scrub for decane (<−2); these alkanes were the only ones described using scrubbing and diffusion limitation for all tissues.

Similar to QPC, arterial blood and brainstem tissue dose metrics were highly sensitive to cardiac output (QCC) for toluene, ethylbenzene, and xylenes, and moderately sensitive for naphthalene, with increases in QCC resulting in decreases in concentration. Liver tissue dose metrics had medium to low sensitivity to QCC for toluene, ethylbenzene, and xylenes; conversely, these displayed a positive correlation (i.e., increase in output with an increase in QCC). All dose metrics for nonane and decane, and liver tissue dose metrics for naphthalene had minimal sensitivity to QCC (i.e., sensitivity coefficients < 0.1 in absolute value).

Arterial blood and brainstem tissue AUC and peak concentrations were highly or moderately sensitive to fractional blood flow to liver (QLivC) and rapidly perfused tissues (QRapC) for toluene, ethylbenzene, xylenes, and naphthalene; QLivC coefficients were negative while QRapC were positive. For toluene and xylenes only, arterial blood and brainstem tissue dose metrics also had positive low sensitivity coefficients for the fractional blood flow in slowly perfused tissues (QSlwC). Coefficients for nonane and decane were all less than 0.1 in absolute value for these dose metrics for the remaining physiological parameters. Liver tissue dose metrics had positive medium sensitivity to QLivC for toluene and positive low sensitivity for ethylbenzene and xylenes. All other liver tissue coefficients for toluene, ethylbenzene and xylenes for the remaining physiological parameters were less than 0.1 in absolute value. Except for a negative high coefficient for body weight (BW) for nonane, all liver tissue coefficients for nonane, decane and naphthalene for the remaining physiological parameters were less than 0.1 in absolute value.

Each dose metric had at least positive low sensitivity coefficients for the blood:air partition (PB) for all chemicals except naphthalene, for which the sensitivity coefficients were less than 0.1 in absolute value. The sensitivity coefficients were high for nonane (approximately 0.75) and decane (almost 1), were medium for toluene, and were low for ethylbenzene and xylenes. Dose metrics for brainstem tissue and liver tissue were highly sensitive (coefficients of 1) to their respective tissue:blood partitions (PBrnStm and PLiv) for all chemicals. Liver tissue dose metrics were highly sensitive (~0.75) to the liver permeability coefficient (PALiv) for nonane. Sensitivity coefficients were all less than 0.1 in absolute value for all dose metrics and chemicals for the remaining partition and permeability coefficients.

Sensitivity coefficients for the maximal rate of reaction in the liver (VMaxC) were all negative, as an increase in metabolism resulted in a decrease in the tissue dose. Liver affinity constants (Km) had the same magnitude yet opposite (positive) correlation from VMaxC, confirming that metabolism was in the linear, not saturable, range for each compound at the exposure concentration used for this analysis. Except for nonane and decane, VMaxC sensitivity coefficients for the chemicals were, in absolute value, all 0.9 or higher for liver tissue dose metrics. The VMaxC coefficient for nonane was ranked high, but ranked low for decane. For arterial blood and brainstem tissue dose metrics, sensitivity coefficients for VMaxC and Km were medium for ethylbenzene and naphthalene, low for xylenes, and less than 0.1 in absolute value for the remaining chemicals.

## 4. Discussion

The mixtures array model developed for this work met the objectives of the project. It gave tissue dose predictions for six JP-8 components along the peripheral and central auditory pathways. The array structure allows for easy addition of new compounds to the mixture; future expansion will be limited primarily by data availability for new mixture components. The structure allowed for one compound or any combination of compounds to be simultaneously simulated without the necessity of changing the array size every time.

Validation for the individual compounds for many tissues was not possible due to a lack of kinetic data. In particular, the validity of predictions for auditory signaling tissues (cochlea, brainstem, and temporal lobe) are unknown as they simply are not measured in kinetic studies. Furthermore, the model was found to be sensitive to several non-measured (predicted by QSPR or fit) parameters for the compounds best modeled using diffusion limitation (nonane, decane, and naphthalene). However, the predictions generated served the general purpose of informing likely tissue concentrations in order to compare tissues to each other or evaluate fuel exposure scenarios.

A sensitivity analysis was performed to investigate the dependency of the model on uncertain parameters. Of the 56 model parameters varied in the sensitivity analysis, dose metrics showed a sensitivity level ranked low, medium, or high to only 13 of them. Of these 13, several are well described in published literature, making any level of sensitivity less concerning as it contributes a low level of uncertainty. These parameters are cardiac output (QCC), ventilation rate (QPC) and the fractional blood flows to liver (QLivC) and rapidly (QRapC) and slowly (QSlwC) perfused tissues. Studies often report body weights (BW) and accepted default BW values are readily available from various sources (e.g., U.S. Environmental Protection Agency [38]).

Partition coefficients are often measured; however, in the work presented here, some were estimated using QSPR techniques, adding to uncertainty in the values used. The dose metrics chosen were notably sensitive to only three PCs: the blood:air (PB), liver:blood (PLiv), and rapidly perfused:blood (PRap) partitions. Permeability coefficients were utilized for nonane and decane and two tissue compartments for naphthalene. Liver dose metrics were only sensitive to the liver permeability coefficient (PALiv) for nonane, but as the sensitivity ranked high, the uncertainty for this parameter increased. Nonane liver concentration data from one study [54] were adequately simulated (see the Supplemental Materials), improving the confidence in this parameter to some extent.

Models are typically sensitive to liver metabolism parameters (VMaxC and Km), particularly when predicting liver dose metrics. These parameters are often fitted to data, although measured values are sometimes available. The higher level of sensitivity of liver dose metrics helps underscore the importance of having good estimates for these parameters. Several values used herein were set in previously developed models, which should increase confidence.

The last parameter to which dose metrics were sensitive was the fraction of scrubbing (Scrub); all dose metrics were highly sensitive for this parameter for nonane and decane, the only two chemicals that used this parameter. Sensitivity coefficients for decane were much higher than for nonane (approximately −2.3 versus −0.67), likely due to the higher percentage of dose scrubbed for decane than nonane (70% versus 40%). The decane Scrub parameter was set using a published fit value [54]. The level of uncertainty is greatly increased here given the high sensitivity of the dose metrics to this parameter and that the values for both chemicals were fitted. Overall, the sensitivity analysis indicated high sensitivity to six uncertain parameters; few of these parameters can be measured to improve uncertainty in their values.

An additional objective for building the array model was to generate tissue concentration predictions for the Guthrie et al. [14] hearing loss study. Tissue concentration comparisons were of interest to potentially explain the lack of peripheral (cochlea) hearing damage paired with CAPD effects, which occur in the nuclei of the auditory neural pathway in the brainstem and temporal lobe; those effects were further explored in subsequent publications [15,16,17]. The array model predictions indicated that nonane and decane concentrations in the brainstem might be expected to be about nine times higher than blood levels. Nonane and decane are major components of white spirits, a solvent known to affect the central auditory pathway [21,22]. The brainstem was found to contain the highest lipid content of the brain regions tested in prior work [37,45]; Chavko et al. [55] reported twice as much total lipid in the brainstem as compared to the frontal and temporal lobes. Therefore, the highly lipophilic n-alkanes in the mixture would partition preferentially into the brainstem.

Any prediction of chemical concentrations in the brain should consider the presence of the blood-brain barrier (BBB). The BBB is the system of tight junctions between endothelial cells lining brain blood vessels that prevent large or polar compounds from crossing from the blood supply into brain tissue. Small hydrophobic compounds, such as the JP-8 components in the array model, cross the BBB passively [56,57]. Lof et al. [46] measured brain concentrations of nonane and decane following a rat inhalation exposure to dearomatized white spirits. The array model successfully simulated these data as total brain concentrations (summation of brainstem, temporal lobe, and remainder of brain tissue compartments). These simulations, seen in the Supplemental Materials, increased the confidence in model predictions for nonane and decane in the general brain. Differential predictions of brainstem or temporal lobe concentrations relied solely on QSPR predicted PCs [44] using target tissue water, protein, and lipids composition of those tissues [45].

It should be noted that many data sets used in model parameterization favored male rats. One toluene study [58] and a nonane study [54] were performed with female rats instead of male. Two naphthalene studies [53,59] were conducted with both male and female rat populations. While the model was able to adequately describe male and female data sets using study specific body weights (no other parameters were altered), parallel data for each mixture component would be needed to determine if the model consistently simulates equally well for males and females. Future in vivo or in vitro research into the kinetics of JP-8 components should include both male and female populations.

The final objective for the array model was to be able to compare tissue kinetics for similar mixtures, such as traditional petroleum fuels and synthetic alternative fuels. To this end, the array model was used to predict tissue levels of the six components for rats exposed at the established OEL (200 mg/m^3^ [48]) to petroleum-derived JP-8 and a 50:50 blend of JP-8 with Virent SAK. Use of the 50:50 blend decreased nonane and decane exposure concentrations by about 50%; this decrease was mirrored in the predicted brainstem concentrations. However, toluene exposure increased by 144% with a resulting 200% increase in predicted maximum blood concentrations. Although use of this SAK may decrease potential CAPD effects by reducing overall exposure to nonane and decane, the non-linear increase in toluene blood concentrations indicate that toluene processes (metabolism, excretion, or fat deposition) may be saturated during this exposure. The use of the SAK would decrease petroleum dependency but may not improve potential toxicity of fuel exposure.

The array PBPK model for simulating mixtures continues to be expanded and modified. Development began several years ago in what is now an unavailable and unsupported software, acslX; for future use, a modeling platform that has the capability to allow the use of arrays for model constants and variables, as well as the ability to integrate across arrays, will be sought for model conversion. Future versions of the model will be parameterized for human simulations; parameterization of the auditory tissues present the main issue for incorporating human capabilities in the model.

Metabolic inhibition, recently featured in the Ruiz et al. [29] mixtures model, should also be added to the array model in the next iteration. Jet fuel components are metabolized by relatively few pathways. Aromatic class compound metabolism is primarily mediated by cytochrome P450 mixed function oxidase system, subfamily CYP2E1. Lighter weight aliphatics such as n- and iso-alkanes are metabolized through CYP1A2 and CYP2B6 pathways [28]. The code is capable of this addition; Ruiz’s work [29] was closely duplicated by the array model in a trial (simulations not shown). However, sufficient binary mixture inhibition constants (Ki) only exist for pairs of toluene, ethylbenzene, and xylenes in rats [47], preventing incorporation of inhibition in this version of the model. For chemical pairs with unavailable Ki, inhibition would have to be assumed to be zero or possibly additive if the same P450 is involved in their metabolism. At this point, the incorporation of metabolic interaction is not expected to have a visible effect at OEL exposure concentrations. The predicted maximal venous blood concentrations (Figure 5 and Figure 6) are below the *Km* values (Table 3); significant competitive metabolic inhibition is not anticipated. Similarly, metabolic interaction was not seen in humans exposed to pairs of these compounds (toluene, ethylbenzene, and xylenes), with respect to expected metabolism data from single compound exposures at similar concentrations to the OEL [60,61]. Competitive metabolic inhibition could become more important to the array model if the whole fuel is accounted for through lumping, thereby increasing the additive metabolic load on each cytochrome subfamily.

Future expansion of the model may target trimethylbenzenes, major components of Virent SAK. Additionally, many jet fuel components of similar molecular weight exhibit qualitatively similar pharmacokinetic properties in living organisms, which makes them suitable to lump with similar components for modeling. This concept has been explored using traditional PBPK modeling techniques [28,62] and can be incorporated relatively easily using the array model. Components of interest could be tracked and the rest of the fuel accounted for in aliphatic and aliphatic lumped fractions using the equivalent carbon fractions set up by the Total Petroleum Hydrocarbon Working Group (TPHCWG) [63]. Although the TPHCWG chemical groupings were established for soil cleanup purposes, the fraction constructs were based on general chemical properties, which not only influence the migration of the compounds in soil and groundwater, but also affect their partitioning in tissues.

## 5. Conclusions

In conclusion, the jet fuel component array model fulfilled the objectives for the project. Those objectives were to describe tissue doses of multiple jet fuel components simultaneously, describe tissue doses for peripheral and central auditory pathway tissues, allow for easy expansion to new compounds, enable validation of one compound at a time without simulation of all compounds, and allow for easy exposure scenario changes for comparison of fuels on tissue concentration. Once converted into a more sustainable modeling platform, this work has the potential to simultaneously predict new mixture tissue concentrations outside the realm of jet fuel components. The model incorporated both flow and diffusion limited options, Michaelis-Menten kinetics, and urinary excretion; these capabilities allow for model expansion to different chemical exposure scenarios.

## Figures and Tables

**Table 1:** Physiological constants for rat simulations.

Constant	Constant Name	Value ^a^
*BW*	Body weight (kg)	0.25 ^b^
*QCC*	Cardiac output (L/(h·kg^0.75^))	14.6
*QPC*	Alveolar/pulmonary flow (L/(h·kg^0.75^))	24.75
**Tissue Blood Flows [fraction of cardiac output]**		
*QBrC*	Remainder of brain tissues	0.013
*QBrnStmC*	Brainstem	0.004
*QBrnTLC*	Temporal lobe	0.003
*QCocC*	Cochlea	0.00004 ^d^
*QFatC*	Fat	0.07
*QLivC*	Liver	0.183
*QLngC*	Lung	0.021 ^c^
*QRapC*	Rapidly perfused	0.536
*QSlwC*	Slowly perfused	0.17
**Tissue Volumes [fraction of BW] ^e^**		
*VAlvC*	Alveolar blood	0.007
*VBrnC*	Remainder of brain tissue	0.004 ^f^
*VBrnStmC*	Brainstem	0.001 ^f^
*VBrnTLC*	Temporal lobe	0.001 ^g^
*VFatC*	Fat	0.10
*VLivC*	Liver	0.034
*VLngC*	Lung	0.005 ^c^
*VRapC*	Rapidly perfused	0.039
*VSlwC*	Slowly perfused	0.65
**Tissue Blood Volumes [fraction of tissue volume]**		
*VBrnBldC*	Remainder of brain tissue	0.03 ^c^
*VBrnStmBldC*	Brainstem	0.03 ^c^
*VBrnTLBldC*	Temporal lobe	0.03 ^c^
*VCocBldC*	Cochlea	0.0183 ^h^
*VFatBldC*	Fat	0.0154 ^c^
*VLivBldC*	Liver	0.21 ^c^
*VLngBldC*	Lung	0.36 ^c^
*VRapBC*	Rapidly perfused	0.2075 ^c^
*VSlwBldC*	Slowly perfused	0.0333 ^i^

^a^ All parameters are from Clewell et al. [30] source model, unless noted otherwise. ^b^ Model used study specific BW whenever provided; default is U.S. EPA value [38]. ^c^ [39]. ^d^ Average reference value [40]. ^e^ Cochlea volume was calculated using Equation 1. ^f^ Total brain value with VBrnStmC and VBrnTLC subtracted [41]. ^g^ Mean weight [37]. ^h^ [42]. ^i^ Average of slowly perfused tissues, Table 30 [39].

**Table 2:** Array PBPK partition and permeability coefficients for key hydrocarbons.

Constant	Constant Name	Toluene	Ethylbenzene	Xylenes	Nonane	Decane	Naphthalene
*MW*	Molecular weight	92.14 ^a^	106.16 ^a^	106.16 ^a^	128.25 ^a^	142.28 ^a^	128.17 ^a^
*PB*	Blood:air PC	18.0 ^b^	42.7 ^b^	46.0 ^b^	5.2 ^c^	5.0 ^c^	571.0 ^g^
**Tissue:Blood Partition Coefficients (unitless)**							
*PBrn*	Brain	2.0 ^c^	1.22 ^c^	1.38 ^c^	5.0 ^c^	10.0 ^c^	3.5 ^h^
*PStm*	Brainstem	2.87 ^c^	1.93 ^c^	2.29 ^c^	(2.9/1.7) × PBrn ^f^	(1.56/1.75) × PBrn ^f^	3.5 ^h^
*PTL*	Temporal lobe	2.13 ^c^	1.44 ^c^	1.61 ^c^	(2.6/1.7) × PBrn ^f^	(1.16/1.75) × PBrn ^f^	3.5 ^h^
*PCoc*	Cochlea	0.54 ^c^	0.44 ^c^	0.47 ^c^	1.45 ^c^	2.15 ^c^	0.47 ^h^
*PFat*	Fat	56.7 ^b^	36.4 ^b^	40.4 ^b^	282.0 ^c^	328.0 ^c^	49.0 ^g^
*PLiv*	Liver	4.64 ^b^	1.96 ^b^	1.98 ^b^	8.0 ^c^	3.0 ^c^	1.6 ^g^
*PLng*	Lung	4.64 ^d^	1.41 ^d^	1.98 ^d^	2.0 ^d^	3.0 ^d^	3.5 ^g^
*PRap*	Rapidly perfused	4.64 ^c^	1.41 ^c^	1.98 ^b^	2.0 ^c^	3.0 ^c^	3.5 ^g^
*PSlw*	Slowly perfused	1.54 ^b^	0.61 ^b^	0.91 ^b^	4.0 ^3^	0.85 ^c^	3.5 ^g^
**Permeability Coefficients (L/h)**							
*PABrn*	Brain	10,000 ^e^	10,000 ^e^	10,000 ^e^	0.5 ^c^	0.005 ^c^	10,000 ^e^
*PAStm*	Brainstem	10,000 ^e^	10,000 ^e^	10,000 ^e^	0.5 ^c^	0.005 ^c^	10,000 ^e^
*PATL*	Temporal lobe	10,000 ^e^	10,000 ^e^	10,000 ^e^	0.5 ^c^	0.005 ^c^	10,000 ^e^
*PACoc*	Cochlea	10,000 ^e^	10,000 ^e^	10,000 ^e^	l.0 ^c^	1.0 ^c^	10,000 ^e^
*PAFat*	Fat	10,000 ^e^	10,000 ^e^	10,000 ^e^	0.5 ^c^	0.07 ^c^	(0.2 × BW^0.75^) ^h^
*PALiv*	Liver	10,000 ^e^	10,000 ^e^	10,000 ^e^	0.07 ^c^	0.15 ^c^	10,000 ^e^
*PALng*	Lung	10,000 ^e^	10,000 ^e^	10,000 ^e^	1.0 ^c^	0.005 ^c^	10,000 ^e^
*PARap*	Rapidly perfused	10,000 ^e^	10,000 ^e^	10,000 ^e^	l.0 ^c^	0.005 ^c^	10,000 ^e^
*PASlw*	Slowly perfused	10,000 ^e^	10,000 ^e^	10,000 ^e^	0.5 ^c^	0.14 ^c^	(2.0 × BW^0.75^) ^h^

^a^ https://pubchem.ncbi.nlm.nih.gov/ (accessed on 18 January 2023); ^b^ [47]; ^c^ [37]; ^d^ Lung parameters = rapidly perfused tissues, in the absence of data; ^e^ No diffusion limitation; ^f^ Predicted PC using ratio of brain region/total brain tissue composition data [45] and fit values [37] to total brain concentration data [46]; ^g^ [35]; ^h^ Values were not pre-scaled to BW [36].

**Table 3:** Array PBPK inhalation, metabolic, and clearance parameters for key hydrocarbons.

Constant	Constant Name	Toluene	Ethylbenzene	Xylenes	Nonane	Decane	Naphthalene
*VmaxC*	Maximal rate of reaction–liver (mg/(h kg^0.75^))	3.44 ^a^	6.39 ^a^	6.49 ^a^	0.1 ^e^	0.005 ^e^	((8.28 × BW)/ BW^0.75^) ^g^
*Km*	Affinity constant–liver (mg/L)	0.13 ^a^	1.04 ^e^	0.45 ^a^	0.1 ^e^	0.1 ^e^	2.18 ^g^
*VmaxLngC*	Maximal rate of reaction–lung (mg/(h kg^0.75^))	0 ^b^	0 ^b^	0 ^b^	0 ^b^	0 ^b^	((0.45 × BW)/ BW^0.75^) ^g^
*KmLng*	Affinity constant–lung (mg/L)	1 ^b^	1 ^b^	1 ^b^	1 ^b^	1 ^b^	2.18 ^g^
*ClUrC*	Urinary clearance (L/(h kg^0.75^))	0.004 ^c^	0.04 ^e^	0.004 ^c^	0.4 ^e^	0.004 ^c^	0 ^h^
*Scrub*	Upper respiratory tract scrubbing (fraction)	0 ^d^	0 ^d^	0 ^d^	0.4 ^e^	0.7 ^f^	0 ^d^

^a^ [47]; ^b^ Assumption of no significant metabolism in lung (*VMaxLngC* = 0 & *KmLng* = 1 to avoid division by 0); ^c^ Default if urinary clearance assumed [30]; ^d^ Default if no URT scrubbing assumed; ^e^ Fit using available kinetic data [37]; ^f^ [31]; ^g^ Merrill et al. [36] reported the maximum rate of reaction values in mg/(h kg); this equation allows use of these units in the model, which scales to BW^0.75^; ^h^ Assumed 100% metabolism.

**Table 4:** Percent Volume Values of Key Components in JP-9 and Virent SAK.

Compound	Compound Percent by Volume			% Difference
	JP-8	Virent SAK	50-50 Blend	Blend vs. JP-8
Toluene	0.16	0.30	0.23	144
Ethylbenzene	0.11	0.17 ^1^	0.14 ^2^	127 ^2^
Xylenes	0.67	0.50 ^1^	0.59 ^2^	87 ^2^
Nonane	1.14	0.09	0.62	54
Decane	2.55	0.13	1.34	53
Naphthalene	0.12	0.02	0.07	58

^1^ The total C2-benzene content in Virent SAK is 0.67% by weight. This category includes ethylbenzene and xylenes. The total C2-benzene content was divided by 4, the number of compounds possible in the category. One portion of the content was assigned to ethylbenzene and the remaining three portions were assigned to o-, m-, and p-xylene as a group. ^2^ Values are approximate, based on assumptions outlined in ^1^.

**Table 5:** Array Model Normalized Sensitivity Coefficient Categorization.

Sensitivity Category ^1^	Toluene	Ethyl Benzene	Xylenes	Nonane	Decane	Naphthalene
**Arterial Blood Concentration (Peak and AUC)**						
High	QCC-, QPC+, QLivC-	QCC-, QPC+	QCC-, QPC+, QLivC-	PB+, Scrub-	PB+, **Scrub-**	**QPC+**
Medium	QRapC+, PB+	QLivC-, QRapC+, VMaxC-, Km+	QRapC+	QPC+		QCC-, QLivC-, QRapC+, VMaxC-, Km+
Low	QSlwC+	PB+	QSlwC+, PB+, VMaxC-, Km+			
**Brainstem Concentration (Peak and AUC)**						
High	QCC-, QPC+, QLivC-, **PBrnStm+**	QCC-, QPC+, **PBrnStm+**	QCC-, QPC+, QLivC-, **PBrnStm+**	PB+, **PBrnStm+**, Scrub-	PB+, **PBrnStm+**, **Scrub-**	**QPC+**, **PBrnStm+**
Medium	QRapC+, PB+	QLivC-, QRapC+, VMaxC-, Km+	QRapC+	QPC+		QCC-, QLivC-, QRapC+, VMaxC-, Km+
Low	QSlwC+	PB+	QSlwC+, PB+, VMaxC-, Km+			
**Liver Concentration (Peak and AUC)**						
High	QPC+, **PLiv+**, **VMaxC-**, **Km+**	QPC+, **PLiv+**, VMaxC-, Km+	QPC+, **PLiv+**, **VMaxC-**, **Km+**	BW-, PB+, **PLiv+**, PALiv+, VMaxC-, Km+, Scrub-	PB+, **PLiv+**, **Scrub-**	**QPC+**, **PLiv+**, VMaxC-, Km+
Medium	QCC+, QLivC+, PB+			QPC+		
Low	QRapC-	QCC+, QLivC+, QRapC-, PB+	QCC+, QLivC+, PB+		VMaxC-, Km+	

^1^ Categories were assigned based on recommended limits [32]. Sensitivity coefficients with bolded parameter names were greater than 0.95 in absolute value. Normalized sensitivity coefficient ratings were identical for each tissue’s Cmax and AUC, and coefficients, rounded to 2 decimal places, were almost identical. “+” and “-” at the end of the parameter names indicates whether the sensitivity coefficients were positive or negative, respectively.

## Data Availability

Data are available in the supplemental material and published resources.

## Supplementary Materials

The following supporting information can be downloaded at: https://www.mdpi.com/article/10.3390/toxics11020187/s1, Supplementary S1: Complex Mixtures Array Model Code; Supplementary S2: Complex Mixtures Array Model Simulations.

## Disclaimer

The views presented herein are those of the authors and do not reflect the official guidance or position of the United States Government, the Department of Defense (DoD), or of the United States Air Force. Appearance of, or reference to, any commercial products or services does not constitute DoD endorsement of these products or services. The appearance of external hyperlinks does not constitute endorsement by the United States DoD of the linked websites, or the information, products or services contained therein. The DoD does not exercise any editorial, security, or other control over the information you may find at these locations.
