# Supplementary material for: Complex Mixtures: Array PBPK Modeling of Jet Fuel Components
Source: Toxics. 2023 Feb 17;11(2):187. doi: 10.3390/toxics11020187 (PMC9964161; doi:10.3390/toxics11020187)
Supplement: Supplementary file 1 [file toxics-11-00187-s001.zip › S1-Complex Mixtures Array Model Code.pdf]

toxics MDPI

Supplementary Materials S1:

Complex Mixtures Array Model Code

Teresa R. Sterner [1,2,*], Tammie R. Covington [1,2] and David R. Mattie [2]

[1] Henry M. Jackson Foundation for the Advancement of Military Medicine; Wright-Patterson AFB, OH
[2] Air Force Research Laboratory, 711HPW/RHBAF; Wright-Patterson AFB, OH
* Correspondence: tsterner@hjfresearch.org

1. acslX Array Model Code

The code pasted in sections below may have artificial breaks imposed by formatting; these additional line breaks and spaces may need to be removed for the .csl and .m files to run correctly.

2. Array Model .csl File

```
PROGRAM: JFHL_6HC_SA.CSL
! Jet fuel hearing loss model for 6 hydrocarbons -- version "SA" for sensitivity analysis

! Adapted from version of Isopropanol/Acetone model using arrays and from 4 Chem model

! Model currently set up to run for 6 chemicals
! Will run for fewer chemicals without modifications as long as parameters are set accordingly
! Can change the model to run for more or less by only changing the "6" in the line "PARAMETER (NChem=6)"


! *******************  SET INIT.M TO LOAD AT RUN TIME  *****************


INITIAL
  INTEGER NChem
  PARAMETER (NChem=6)

  DIMENSION MW(NChem)
  DIMENSION PB(NChem), PBrnStm(NChem), PBrnTL(NChem), PBrn(NChem), PCoc(NChem), PFat(NChem), PLiv(NChem),
PLng(NChem), PRap(NChem), PSlw(NChem)
  DIMENSION PABrnStm(NChem), PABrnTL(NChem), PABrn(NChem), PACoc(NChem), PAFat(NChem), PALiv(NChem),
PALng(NChem), PARap(NChem), PASlw(NChem)
  DIMENSION VMaxC(NChem), KM(NChem), KFC(NChem), VMaxLngC(NChem), KMLng(NChem)
  DIMENSION ClUrC(NChem), Scrub(NChem)
  DIMENSION Conc(NChem), IVDose(NChem), PDose(NChem), PDrink(NChem)
  DIMENSION kAD(NChem), kAS(NChem), kTD(NChem), kTSD(NChem)
  DIMENSION AI0(NChem), IVR(NChem), Drink(NChem), TotDose(NChem)
  DIMENSION VMax(NChem), KF(NChem), VMaxLng(NChem)
  DIMENSION ClUr(NChem)
  DIMENSION IAArt(NChem), IABrnStm(NChem), IABrnStmBld(NChem), IABrnTL(NChem), IABrnTLBld(NChem),
IABrn(NChem), IABrnBld(NChem)
  DIMENSION IACoC(NChem), IACocBld(NChem), IAFat(NChem), IAFatBld(NChem), IALiv(NChem), IALivBld(NChem),
IALng(NChem), IALngBld(NChem)
  DIMENSION IARap(NChem), IARapBld(NChem), IASlw(NChem), IASlwBld(NChem)
  DIMENSION IAExh(NChem), IAO(NChem), IAExc(NChem), IADu(NChem), IASt(NChem), IAMet1(NChem), IAMet2(NChem),
IAMetLng(NChem)
  DIMENSION IAUrn(NChem), IAUCCArt(NChem), IAUCCBrnStm(NChem), IAUCCBrn(NChem), IAUCCLiv(NChem),
IATotInh(NChem), IATotIV(NChem), IATotDrink(NChem)
  DIMENSION PerEnd(NChem), PerMix(NChem)
  DIMENSION CAlv(NChem), CAlvPPM(NChem), CP(NChem), CEnd(NChem), CEndPPM(NChem), CMix(NChem), CMixPPM(NChem),
CBrnTotBld(NChem), CBrnTot(NChem)
  DIMENSION ACh(NChem), CInh(NChem), RACh(NChem), AArt(NChem), AExh(NChem), CArt(NChem), AUCCArt(NChem)
  DIMENSION CVen(NChem), RAArt(NChem), RAExh(NChem)
  DIMENSION ABrnStm(NChem), ABrnStmBld(NChem), CBrnStm(NChem), AUCCBrnStm(NChem), CBrnStmBld(NChem),
RABrnStm(NChem), RABrnStmBld(NChem)
  DIMENSION ABrnTL(NChem), ABrnTLBld(NChem), CBrnTL(NChem), CBrnTLBld(NChem), RABrnTL(NChem),
RABrnTLBld(NChem)
  DIMENSION ABrn(NChem), ABrnBld(NChem), CBrn(NChem), AUCCBrn(NChem), CBrnBld(NChem), RABrn(NChem),
RABrnBld(NChem)
  DIMENSION ACoc(NChem), ACocBld(NChem), CCoc(NChem), CCocBld(NChem), RACoc(NChem), RACocBld(NChem)
  DIMENSION AFat(NChem), AFatBld(NChem), CFat(NChem), CFatBld(NChem), RAFat(NChem), RAFatBld(NChem)
```

```
  DIMENSION AO(NChem), AExc(NChem), ADu(NChem), ASt(NChem), RAO(NChem), RAExc(NChem), RADu(NChem),
RASt(NChem)
  DIMENSION ALiv(NChem), ALivBld(NChem), AMet1(NChem), AMet2(NChem), CLiv(NChem), CLivBld(NChem),
AUCCLiv(NChem), RAMet1(NChem), RAMet2(NChem), RALiv(NChem), RALivBld(NChem)
  DIMENSION ALng(NChem), ALngBld(NChem), AMetLng(NChem), CLng(NChem), CLngBld(NChem), RAMetLng (NChem),
RALng(NChem), RALngBld(NChem)
  DIMENSION ARap(NChem), ARapBld(NChem), CRap(NChem), CRapBld(NChem), RARap(NChem), RARapBld(NChem)
  DIMENSION ASlw(NChem), ASlwBld(NChem), CSlw(NChem), CSlwBld(NChem), RASlw(NChem), RASlwBld(NChem)
  DIMENSION AUrn(NChem), RAUrn(NChem)
  DIMENSION ATotInh(NChem), ATotIV(NChem), ATotDrink(NChem), RATotInh(NChem), TDose(NChem), AmtBody(NChem),
MassBal(NChem)


  INTEGER i, j1, j2, j3, i1, i2, i3, i4, i5, i6, i7, i8, i9, i10, i11, i12, i13, i14, i15, i16
  LOGICAL CC                        ! To control whether closed or open chamber
  LOGICAL AdjFlows                  ! NEEDED FOR SENSITIVITY ANALYSIS -- to control adjusting tissue blood
flows if fractions don't sum to one (will recalculate QC regardless)
  LOGICAL AdjVols                   ! NEEDED FOR SENSITIVITY ANALYSIS -- to control adjusting tissue vol-
umes if fractions don't sum to set body total


! ---------------- PHYSIOLOGICAL PARAMETERS -----------------------
  CONSTANT        BW = 0.25         ! Body weight (kg)
  CONSTANT       QCC = 14.6         ! Cardiac output (L/hr/kg^0.75)
  CONSTANT       QPC = 24.75        ! Total pulmonary ventilation rate (L/hr/kg^0.75)
  CONSTANT  AdjFlows = .FALSE.      ! Don't adjust tissue blood flows (but does still recalculate QC as
total of tissue flows)
  CONSTANT   AdjVols = .FALSE.      ! Don't adjust tissue blood volumes

! Blood Flows (fraction of cardiac output)
  CONSTANT  QBrnStmC = 0.004        ! Brain stem
  CONSTANT   QBrnTLC = 0.003        ! Temporal lobe of brain
  CONSTANT     QBrnC = 0.013        ! Remainder of brain (0.02 - QBrnStmC - QBrnTLC)
  CONSTANT     QCocC = 0.00004      ! Cochlea (pair)
  CONSTANT     QFatC = 0.07         ! Fat
  CONSTANT     QLivC = 0.183        ! Liver
  CONSTANT     QLngC = 0.021        ! Lung (Brown et al., 1997)
  CONSTANT     QRapC = 0.536        ! Rapidly perfused tissues (0.557 - QLngC)
  CONSTANT     QSlwC = 0.17         ! Slowly perfused tissues (includes cochlea)

! Tissue Volumes (fraction of body weight)
! (VCocMC is based on data for rat cochlea pair mass (kg)
  CONSTANT     VAlvC = 0.007        ! Alveolar blood
  CONSTANT  VBrnStmC = 0.001        ! Brain stem
  CONSTANT   VBrnTLC = 0.001        ! Temporal lobe of brain
  CONSTANT     VBrnC = 0.004        ! Remainder of brain (0.006 - VBrnStmC - VBrnTLC)
  CONSTANT    VCocMC = 0.00007      ! Cochlea (pair) -- THIS ISN'T FRACTIONAL -- is multiplied by
BW^0.2348
  CONSTANT     VFatC = 0.10         ! Fat
  CONSTANT     VLivC = 0.034        ! Liver
  CONSTANT     VLngC = 0.005        ! Lung (Brown et al 1997)
  CONSTANT     VRapC = 0.039        ! Rapidly perfused tisues (0.044 - VLngC)
  CONSTANT     VSlwC = 0.65         ! Slowly perfused tissues (includes cochlea)
  CONSTANT    VBodyC = 0.841        ! Sum of fractional volumes
  CONSTANT        DS = 0.15         ! Dead space volume (fraction)

! Tissue Blood Volumes (fraction of tissue volume)
  CONSTANT VBrnStmBldC = 0.03       ! Brain stem
  CONSTANT  VBrnTLBldC = 0.03       ! Temporal lobe of brain
  CONSTANT    VBrnBldC = 0.03       ! Remainder of brain
  CONSTANT    VCocBldC = 0.0183     ! Cochlea
  CONSTANT    VFatBldC = 0.0154     ! Fat
  CONSTANT    VLivBldC = 0.21       ! Liver
  CONSTANT    VLngBldC = 0.36       ! Lung (Brown et al., 1997)
  CONSTANT    VRapBldC = 0.2075     ! Rapidly perfused tissues
  CONSTANT    VSlwBldC = 0.0333     ! Slowly perfused tissues

! Simulation Control Parameters (hrs)
  CONSTANT     TStop = 24.0
  CINTERVAL     CINT = 0.01


! ---------------- DOSING PARAMETERS ------------------------------
! Dose Timing Parameters
  CONSTANT    DaysWk = 1.0          ! Number of exposure days per week (days)
  CONSTANT   DoseInt = 1000.0       ! Interval to repeat dosing (hrs)
```

```
  CONSTANT      TMax = 24.0            ! Maximum time for exposures (hrs)

! Inhalation Exposure Parameters
  CONSTANT      Conc = NChem*0.0       ! Inhaled concentration (ppm)
  CONSTANT     TChng = 0.0             ! Length of inhalation exposure (hrs)

! Closed Chamber Parameters
  CONSTANT        CC = .FALSE.         ! Default to open chamber
  CONSTANT      kLCC = 0.0             ! Chamber loss (/hr)
  CONSTANT      Rats = 1.0             ! Number of animals in experiment
  CONSTANT      VChC = 9.1             ! Volume of closed chamber (L)

! Intravenous Exposure Parameters
  CONSTANT    IVDose = NChem*0.0       ! IV dose (mg/kg)
  CONSTANT      TInf = 0.20            ! Length of IV injection (hrs)

! Oral Exposure Parameters
  CONSTANT       kAD = NChem*0.0       ! Absorption from duodenum (/hr)
  CONSTANT       kAS = NChem*0.0       ! Absorption from stomach (/hr)
  CONSTANT       kTD = NChem*0.0       ! Excretion (/hr)
  CONSTANT      kTSD = NChem*0.0       ! Transfer - stomach to duodenum (/hr)
  CONSTANT     PDose = NChem*0.0       ! Oral dose (mg/kg)
  CONSTANT    PDrink = NChem*0.0       ! Drinking water dose (mg/kg/day)


! ----------------- CHEMICAL SPECIFIC PARAMETERS --------------------
! Molecular Weights (mg/mmole)
  CONSTANT        MW = NChem*1.0

! Tissue/Blood Partition Coefficients (unitless)
  CONSTANT        PB = NChem*1.0       ! Blood/air
  CONSTANT   PBrnStm = NChem*1.0       ! Brain stem
  CONSTANT    PBrnTL = NChem*1.0       ! Temporal lobe of brain
  CONSTANT      PBrn = NChem*1.0       ! Remainder of brain
  CONSTANT      PCoc = NChem*1.0       ! Cochlea
  CONSTANT      PFat = NChem*1.0       ! Fat
  CONSTANT      PLiv = NChem*1.0       ! Liver
  CONSTANT      PLng = NChem*1.0       ! Lung
  CONSTANT      PRap = NChem*1.0       ! Rapidly perfused tissues
  CONSTANT      PSlw = NChem*1.0       ! Slowly perfused tissues

! Tissue Permeability Coefficients (L/hr) (for diffusion limitation)
  CONSTANT  PABrnStm = NChem*1000.0    ! Brain stem
  CONSTANT   PABrnTL = NChem*1000.0    ! Brain temporal lobe
  CONSTANT     PABrn = NChem*1000.0    ! Brain remainder
  CONSTANT     PACoc = NChem*1000.0    ! Cochlea
  CONSTANT     PAFat = NChem*1000.0    ! Fat
  CONSTANT     PALiv = NChem*1000.0    ! Liver
  CONSTANT     PALng = NChem*1000.0    ! Lung
  CONSTANT     PARap = NChem*1000.0    ! Rapidly perfused tissues
  CONSTANT     PASlw = NChem*1000.0    ! Slowly perfused tissues

! Metabolism Parameters
  CONSTANT     VMaxC = NChem*0.0       ! Michaelis-Menten maximum reaction rat for lung metabolism
(mg/hr/kg^0.75)
  CONSTANT        KM = NChem*1.0       ! Michaelis-Menten affinity coefficient (mg/L)
  CONSTANT       KFC = NChem*0.0       ! First order rate constant (/hr)
  CONSTANT  VMaxLngC = NChem*0.0       ! Michaelis-Menten maximum reaction rat for lung metabolism
(mg/hr/kg^0.75)
  CONSTANT     KMLng = NChem*1.0       ! Michaelis-Menten affinity coefficient (mg/L)

! Uptake and Clearance Parameters (L/hr/kg^0.75)
  CONSTANT     ClUrC = NChem*0.0       ! Urinary clearance
  CONSTANT     Scrub = NChem*0.0       ! Inhaled dose scrubbed (fraction) (not absorbed into blood)


! ----------------- PHYSIOLOGICAL PARAMETER SCALING -----------------
! Alveolar Ventilation Rate (L/hr)
        QAlv = 0.67 * (QPC * (BW**0.75))


! Scaled Blood Flows (L/hr)
      QAdjus = 1.0
  IF (AdjFlows) QAdjus = QBrnStmC + QBrnTLC + QBrnC + QFatC + QLivC + QLngC + QRapC + QSlwC
          QC = QCC * (BW**0.75)
     QBrnStm = (QBrnStmC / QAdjus) * QC                    ! Brain stem
```

```
      QBrnTL = (QBrnTLC / QAdjus) * QC                     ! Temporal lobe of brain
        QBrn = (QBrnC / QAdjus) * QC                       ! Remainder of brain
        QCoc = (QCocC / QAdjus) * QC                       ! Cochlea (pair)
        QFat = (QFatC / QAdjus) * QC                       ! Fat
        QLiv = (QLivC / QAdjus) * QC                       ! Liver
        QLng = (QLngC / QAdjus) * QC                       ! Lung
        QRap = (QRapC / QAdjus) * QC                       ! Rapidly perfused tissues
        QSlw = ((QSlwC / QAdjus) * QC) - QCoc              ! Slowly perfused tissues
          QC = QBrnStm + QBrnTL + QBrn + QCoc + QFat + QLiv + QLng + QRap + QSlw


! Tissue Volumes (L)
      VAdjus = 1.0
       VCocC = (VCocMC * (BW**0.2348)) / BW                          ! Data based calculation of cochlea pair
mass (kg)

  IF (AdjFlows) VAdjus = VBodyC / (VAlvC + VBrnStmC + VBrnTLC + VBrnC + VFatC + VLivC + VLngC + VRapC +
VSlwC)
        VAlv = (VAlvC * VAdjus) * BW                                 ! Alveolar blood
     VBrnStm = ((VBrnStmC * VAdjus) * BW) * (1.0 - VBrnStmBldC)      ! Brain stem
      VBrnTL = ((VBrnTLC * VAdjus) * BW) * (1.0 - VBrnTLBldC)        ! Temporal lobe of brain
        VBrn = ((VBrnC * VAdjus) * BW) * (1.0 - VBrnBldC)            ! Remainder of brain
        VCoc = ((VCocC * VAdjus) * BW) * (1.0 - VCocBldC)            ! Cochlea (pair)
        VFat = ((VFatC * VAdjus) * BW) * (1.0 - VFatBldC)            ! Fat
        VLiv = ((VLivC * VAdjus) * BW) * (1.0 - VLivBldC)            ! Liver
        VLng = ((VLngC * VAdjus) * BW) * (1.0 - VLngBldC)            ! Lung
        VRap = ((VRapC * VAdjus) * BW) * (1.0 - VRapBldC)            ! Rapidly perfused tissues
        VSlw = (((VSlwC - VCocC) * VAdjus) * BW) * (1.0 - VSlwBldC)  ! Slowly perfused tissues


! Tissue Blood Volumes (L)
  VBrnStmBld = ((VBrnStmC * VAdjus) * BW) * VBrnStmBldC              ! Brain stem
   VBrnTLBld = ((VBrnTLC * VAdjus) * BW) * VBrnTLBldC                ! Temporal lobe of brain
     VBrnBld = ((VBrnC * VAdjus) * BW) * VBrnBldC                    ! Remainder of brain
     VCocBld = ((VCocC * VAdjus) * BW) * VCocBldC                    ! Cochlea (pair)
     VFatBld = ((VFatC * VAdjus) * BW) * VFatBldC                    ! Fat
     VLivBld = ((VLivC * VAdjus) * BW) * VLivBldC                    ! Liver
     VLngBld = ((VLngC * VAdjus) * BW) * VLngBldC                    ! Lung
     VRapBld = ((VRapC * VAdjus) * BW) * VRapBldC                    ! Rapidly perfused tissues
     VSlwBld = (((VSlwC - VCocC) * VAdjus) * BW) * VSlwBldC          ! Slowly perfused tissues


! ---------------- DOSING PARAMETER SCALING -----------------------
! Initialize Dosing Parameters
      DayExp = 1.0                                         ! Counter for number of days exposed (days)


! Set Chamber Volume and Loss for Open and Closed Chamber Exposures
  IF (.NOT. CC) THEN
      VCh = 1.0e+20                   ! Volume of closed chamber (L) (large chamber = open chamber)
      kLC = 0.0                       ! Chamber loss (/hr)
  ELSE
      VCh = VChC - (Rats * BW)        ! Calculate net chamber volume (L)
      kLC = kLCC                      ! Chamber loss (/hr)
  ENDIF


DO 1010 i=1, NChem
! Inhalation Exposure Parameters
      AI0(i) = (Conc(i) * VCh * MW(i)) / 24450.0                     ! Initial amount in chamber (mg)
      CIZone = 1.0                                                   ! Switch to turn inhalation on and off
for repeated exposure (unitless)

! Intravenous Exposure Parameters
      IVR(i) = 0.0                                                   ! Rate of intravenous dosing (mg/hr)

! Scaled and Initial Doses
    Drink(i) = (PDrink(i) * BW) / 24.0                               ! Drinking water dose (mg/hr)
  TotDose(i) = 0.0                                                   ! Initial total oral dose (mg)
1010: CONTINUE


! ---------------- CHEMICAL SPECIFIC PARAMETER SCALING -------------
DO 1020 i=1, NChem
! Scaled Metabolism Parameters
     VMax(i) = VMaxC(i) * (BW**0.75)
```

```
     KF(i) = KFC(i) / (BW**0.25)
     VMaxLng(i) = VMaxLngC(i) * (BW**0.75)

! Scaled Clearance Rates
     ClUr(i) = ClUrC(i) * (BW**0.75)

! Initialize Initial Values for Integrating State Variables
     IAArt(i) = 0.0
     IABrnStm(i) = 0.0
     IABrnStmBld(i) = 0.0
     IABrnTL(i) = 0.0
     IABrnTLBld(i) = 0.0
     IABrn(i) = 0.0
     IABrnBld(i) = 0.0
     IACoc(i) = 0.0
     IACocBld(i) = 0.0
     IAFat(i) = 0.0
     IAFatBld(i) = 0.0
     IALiv(i) = 0.0
     IALivBld(i) = 0.0
     IALng(i) = 0.0
     IALngBld(i) = 0.0
     IARap(i) = 0.0
     IARapBld(i) = 0.0
     IASlw(i) = 0.0
     IASlwBld(i) = 0.0

     IAExh(i) = 0.0
     IAO(i) = 0.0
     IAExc(i) = 0.0
     IADu(i) = 0.0
     IASt(i) = 0.0
     IAMet1(i) = 0.0
     IAMet2(i) = 0.0
     IAMetLng(i) = 0.0
     IAUrn(i) = 0.0
     IAUCCArt(i) = 0.0
     IAUCCBrnStm(i) = 0.0
     IAUCCBrn(i) = 0.0
     IAUCCLiv(i) = 0.0
     IATotInh(i) = 0.0
     IATotIV(i) = 0.0
     IATotDrink(i) = 0.0

! Initialize Starting Values
     PerEnd(i) = 0.0
     PerMix(i) = 0.0
1020: CONTINUE

END                ! End of Initial


DYNAMIC
  ALGORITHM  IALG = 2                                              ! Gear stiff method


DISCRETE DoseOn    ! Start dosing
  INTERVAL DoseInt = 24.0                                          ! Interval to repeat dosing (hrs)
  SCHEDULE DoseOffIV .AT. T + TInf
  SCHEDULE DoseOffInh .AT. T + TChng

  IF (((T .LT. TMax) .AND. (DayExp .LE. DaysWk)) .OR. CC) THEN
    CIZone = 1.0
  ENDIF

  DayExp = DayExp + 1.0
  IF (DayExp .GT. 7.0) THEN
      DayExp = 0.5
  ENDIF

DO 2010 j1=1, NChem
     IF (T .LE. TInf) THEN
         IVR(j1) = (IVDose(j1) * BW) / TInf                ! Rate of intravenous dosing (mg/hr)
         TotDose(j1) = TotDose(j1) + (PDose(j1) * BW)
     ENDIF
2010: CONTINUE
```

```
END


DISCRETE DoseOffIV
DO 2020 j2=1, NChem
     IVR(j2) = 0.0
2020: CONTINUE
END


DISCRETE DoseOffInh
  IF (.NOT. CC) THEN
      CIZone = 0.0
  ENDIF
END


!-------------------- Calculate Parameters Used for Plotting ----------------------------------------
       Hours = T
     Minutes = T * 60.0
        Days = T / 24.0


DO 2030 i1=1, NChem
! Concentration in Alveolar Air (mg/L and ppm)
     CAlv(i1) = CArt(i1) / PB(i1)
     CAlvPPM(i1) = (CArt(i1) / PB(i1)) * (24450.0 / MW(i1))


! Concentration in Inhaled Air (ppm)
     CP(i1) = (CInh(i1) * 24450.0) / MW(i1)


! Concentration in End-Exhaled Air (mg/L, ppm and percent)
     CEnd(i1) = RAExh(i1) / QAlv
     CEndPPM(i1) = CEnd(i1) * (24450.0 / MW(i1))
     IF (Conc(i1) .GT. 0.0) THEN
         PerEnd(i1) = (CEnd(i1) / ((Conc(i1) * MW(i1)) / 24450.0)) * 100.0
     ENDIF


! Concentration in Mixed Exhaled Air (mg/L, ppm and percent)
     CMix(i1) = ((1.0 - DS) * CEnd(i1)) + (DS * CInh(i1))
     CMixPPM(i1) = CMix(i1) * (24450.0 / MW(i1))
     IF (Conc(i1) .GT. 0.0) THEN
         PerMix(i1) = (CMix(i1) / ((Conc(i1) * MW(i1)) / 24450.0)) * 100.0
     ENDIF


! Calculate Total Brain Tissue and Tissue Blood Concentrations (mg/kg)
     CBrnTot(i1) = (ABrnStm(i1) + ABrnTL(i1) + ABrn(i1)) / (VBrnStm + VBrnTL + VBrn)
     CBrnTotBld(i1) = (ABrnStmBld(i1) + ABrnTLBld(i1) + ABrnBld(i1)) / (VBrnStmBld + VBrnTLBld + VBrnBld)
2030: CONTINUE



DERIVATIVE
!-------------------------------------------------------------------------------------------------
! Amount in Inhaled Air (mg)
         ACh = INTVC(RACh, AI0)

! Concentration in Inhaled Air (mg/L) and Rate of Change in Chamber Concentration (mg/hr)
  PROCEDURAL (CInh, RACh = QAlv, VCh, kLC, CIZone, CArt)
DO 3010 i2=1, NChem
     CInh(i2) = ((ACh(i2) / VCh) * (1.0 - Scrub(i2))) * CIZone
     RACh(i2) = (Rats * ((QAlv * ((CArt(i2) / PB(i2)) - CInh(i2))))) - (kLC * ACh(i2))
3010: CONTINUE
  END


! Amount in Arterial Blood (mg)
        AArt = INTVC(RAArt, IAArt)
     AUCCArt = INTVC(CArt, IAUCCArt)


! Amount in Exhaled Air (mg)
```

```
        AExh = INTVC(RAExh, IAExh)


  PROCEDURAL (CArt = VAlv)
DO 3020 i3=1, NChem
     CArt(i3) = AArt(i3) / VAlv                              ! Concentration of in arterial blood (mg/L)
3020: CONTINUE
  END


  PROCEDURAL (CVen, RAArt, RAExh = QC, QAlv, QBrnStm, QBrnTL, QBrn, QCoc, QFat, QLiv, QLng, QRap, QSlw, CArt,
&
            & CBrnStmBld, CBrnTLBld, CBrnBld, CCocBld, CFatBld, CLivBld, CLngBld, CRapBld, CSlwBld, IVR,
CInh, RAUrn)
DO 3030 i4=1, NChem
! Concentration in Mixed Venous Blood (mg/L)
     CVen(i4) = (QBrnStm*CBrnStmBld(i4) + QBrnTL*CBrnTLBld(i4) + QBrn*CBrnBld(i4) + QCoc*CCocBld(i4) +
QFat*CFatBld(i4) + QLiv*CLivBld(i4) &
            & + QLng*CLngBld(i4) + QRap*CRapBld(i4) + QSlw*CSlwBld(i4) + IVR(i4)) / QC

! Rate of Change in Arterial Blood (mg/hr)
     RAArt(i4) = (QAlv * (CInh(i4) - (CArt(i4) / PB(i4)))) + (QC * (CVen(i4) - CArt(i4))) - RAUrn(i4)

! Rate of Change in Exhaled Air (mg/hr)
     RAExh(i4) = (QAlv * (CArt(i4) / PB(i4)))

3030: CONTINUE
  END


! Brain Stem Tissue and Tissue Blood
     ABrnStm = INTVC(RABrnStm, IABrnStm)                                  ! Amount in brain stem tissue (mg)
  ABrnStmBld = INTVC(RABrnStmBld, IABrnStmBld)                            ! Amount in brain stem blood (mg)
  AUCCBrnStm = INTVC(CBrnStm, IAUCCBrnStm)

  PROCEDURAL (CBrnStm, CBrnStmBld, RABrnStm, RABrnStmBld = QBrnStm, VBrnStm, VBrnStmBld, CArt)
DO 3040 i5=1, NChem
     CBrnStm(i5) = ABrnStm(i5) / VBrnStm
! Concentration in brain stem tissue (mg/L)
     CBrnStmBld(i5) = ABrnStmBld(i5) / VBrnStmBld
! Concentration in brain stem blood (mg/L)
     RABrnStm(i5) = PABrnStm(i5) * (CBrnStmBld(i5) - (CBrnStm(i5) / PBrnStm(i5)))
! Rate of change in brain stem tissue (mg/hr)
     RABrnStmBld(i5) = (QBrnStm * (CArt(i5) - CBrnStmBld(i5))) + (PABrnStm(i5) * ((CBrnStm(i5) / PBrnStm(i5))
- CBrnStmBld(i5))) ! Rate of change in brain stem blood (mg/hr)
3040: CONTINUE
  END


! Brain Temporal Lobe and Tissue Blood
      ABrnTL = INTVC(RABrnTL, IABrnTL)                                    ! Amount in brain temporal lobe
tissue (mg)
   ABrnTLBld = INTVC(RABrnTLBld, IABrnTLBld)                              ! Amount in brain temporal lobe
blood (mg)

  PROCEDURAL (CBrnTL, CBrnTLBld, RABrnTL, RABrnTLBld = QBrnTL, VBrnTL, VBrnTLBld, CArt)
DO 3050 i6=1, NChem
     CBrnTL(i6) = ABrnTL(i6) / VBrnTL
! Concentration in brain temporal lobe tissue (mg/L)
     CBrnTLBld(i6) = ABrnTLBld(i6) / VBrnTLBld
! Concentration in brain temporal lobe blood (mg/L)
     RABrnTL(i6) = PABrnTL(i6) * (CBrnTLBld(i6) - (CBrnTL(i6) / PBrnTL(i6)))
! Rate of change in brain tissue (mg/hr)
     RABrnTLBld(i6) = (QBrnTL * (CArt(i6) - CBrnTLBld(i6))) + (PABrnTL(i6) * ((CBrnTL(i6) / PBrnTL(i6)) -
CBrnTLBld(i6)))   ! Rate of change in brain temporal lobe blood (mg/hr)
3050: CONTINUE
  END


! Remainder of Brain Tissue and Tissue Blood
        ABrn = INTVC(RABrn, IABrn)                                        ! Amount in remainder of brain tis-
sue (mg)
     ABrnBld = INTVC(RABrnBld, IABrnBld)                                  ! Amount in remainder of brain
blood (mg)
     AUCCBrn = INTVC(CBrn, IAUCCBrn)                                      ! Area under the curve for the con-
centration in remainder of brain tissue (hr*mg/L)
```

```
  PROCEDURAL (CBrn, CBrnBld, RABrn, RABrnBld = QBrn, VBrn, VBrnBld, CArt)
DO 3060 i7=1, NChem
     CBrn(i7) = ABrn(i7) / VBrn
! Concentration in brain remainder tissue (mg/L)
     CBrnBld(i7) = ABrnBld(i7) / VBrnBld
! Concentration in brain remainder blood (mg/L)
     RABrn(i7) = PABrn(i7) * (CBrnBld(i7) - (CBrn(i7) / PBrn(i7)))
! Rate of change in brain remainder tissue (mg/hr)
     RABrnBld(i7) = (QBrn * (CArt(i7) - CBrnBld(i7))) + (PABrn(i7) * ((CBrn(i7) / PBrn(i7)) - CBrnBld(i7)))
! Rate of change in brain remainder blood (mg/hr)
3060: CONTINUE
  END


! Cochlea Tissue and Tissue Blood
        ACoc = INTVC(RACoc, IACoc)                                        ! Amount in cochlea tissue (mg)
     ACocBld = INTVC(RACocBld, IACocBld)                                  ! Amount in cochlea blood (mg)

  PROCEDURAL (CCoC, CCoCBld, RACoC, RACoCBld = QCoC, VCoC, VCoCBld, CArt)
DO 3070 i8=1, NChem
     CCoc(i8) = ACoc(i8) / VCoc
! Concentration in cochlea (mg/L)
     CCocBld(i8) = ACocBld(i8) / VCocBld
! Concentration in cochlea blood (mg/L)
     RACoc(i8) = PACoc(i8) * (CCocBld(i8) - (CCoc(i8) / PCoc(i8)))
! Rate of change in cochlea (mg/hr)
     RACocBld(i8) = (QCoc * (CArt(i8) - CCocBld(i8))) + (PACoc(i8) * ((CCoc(i8) / PCoc(i8)) - CCocBld(i8)))
! Rate of change in cochlea blood (mg/hr)
3070: CONTINUE
  END


! Fat Tissue and Tissue Blood
        AFat = INTVC(RAFat, IAFat)                                    ! Amount in fat tissue (mg)
     AFatBld = INTVC(RAFatBld, IAFatBld)                              ! Amount in fat blood (mg)

  PROCEDURAL (CFat, CFatBld, RAFat, RAFatBld = QFat, VFat, VFatBld, CArt)
DO 3080 i9=1, NChem
     CFat(i9) = AFat(i9) / VFat
! Concentration in fat tissue (mg/L)
     CFatBld(i9) = AFatBld(i9) / VFatBld
! Concentration in fat blood (mg/L)
     RAFat(i9) = PAFat(i9) * (CFatBld(i9) - (CFat(i9) / PFat(i9)))
! Rate of change in fat tissue (mg/hr)
     RAFatBld(i9) = (QFat * (CArt(i9) - CFatBld(i9))) + (PAFat(i9) * ((CFat(i9) / PFat(i9)) - CFatBld(i9)))
! Rate of change in fat blood (mg/hr)
3080: CONTINUE
  END


! Digestive Tract
!  Amount Absorbed (mg)
          AO = INTVC(RAO, IAO)

!  Amount Excreted (mg)
        AExc = INTVC(RAExc, IAExc)

!  Amount Duodenum (mg)
         ADu = INTVC(RADu, IADu)

!  Amount Leaving the Stomach (mg)
!  (ASt changed to (INTVC(RASt, IASt)) and is replaced in other equations with (TotDose(i) - ASt(i)) since
ASt
!   could not be defined as (TotDose - INTVC(RASt, 0.0)))
         ASt = INTVC(RASt, IASt)

! Rate of Change in Amount Absorbed, Amount Excreted, Amount in Duodenum and Amount Leaving Stomach (mg/hr)
  PROCEDURAL (RAO, RAExc, RADu, RASt = TotDose)
DO 3090 i10=1, NChem
     RAO(i10) = (kAS(i10) * (TotDose(i10) - ASt(i10))) + (kAD(i10) * ADu(i10))
     RAExc(i10) = kTD(i10) * ADu(i10)
     RADu(i10) = (kTSD(i10) * (TotDose(i10) - ASt(i10))) - (kAD(i10) * ADu(i10)) - (kTD(i10) * ADu(i10))
     RASt(i10) = (kAS(i10) * (TotDose(i10) - ASt(i10))) + (kTSD(i10) * (TotDose(i10) - ASt(i10)))
3090: CONTINUE
  END
```

```
! Liver Tissue and Tissue Blood
        ALiv = INTVC(RALiv, IALiv)                                        ! Amount in liver tissue (mg)
     ALivBld = INTVC(RALivBld, IALivBld)                                  ! Amount in liver blood (mg)
     AUCCLiv = INTVC(CLiv, IAUCCLiv)

! Amount Metabolized in Liver -- Saturable (mg) and 1st Order (mg)
       AMet1 = INTVC(RAMet1, IAMet1)                                      ! Amount metabolized, saturable
(mg)
       AMet2 = INTVC(RAMet2, IAMet2)                                      ! Amount metabolized, first order
(mg)

  PROCEDURAL (CLiv, CLivBld, RALiv, RALivBld, RAMet1, RAMet2 = QLiv, VLiv, VLivBld, VMax, KF, CArt, RAO,
Drink)
DO 3100 i11=1, NChem
     CLiv(i11) = ALiv(i11) / VLiv
! Concentration in liver tissue (mg/L)
     CLivBld(i11) = ALivBld(i11) / VLivBld
! Concentration in liver blood (mg/L)
     RAMet1(i11) = (VMax(i11) * (CLiv(i11) / PLiv(i11))) / (KM(i11) + (CLiv(i11) / PLiv(i11)))
! Saturable rate of metabolism in liver (mg/hr)
     RAMet2(i11) = KF(i11) * (CLiv(i11) / PLiv(i11)) * VLiv
! First-order rate of metabolism in liver (mg/hr)
     RALiv(i11) = (PALiv(i11) * (CLivBld(i11) - (CLiv(i11) / PLiv(i11)))) - RAMet1(i11) - RAMet2(i11) +
RAO(i11) + Drink(i11)    ! Rate of change in liver tissue (mg/hr)
     RALivBld(i11) = (QLiv * (CArt(i11) - CLivBld(i11))) + (PALiv(i11) * ((CLiv(i11) / PLiv(i11)) -
CLivBld(i11)))               ! Rate of change in liver blood (mg/hr)
3100: CONTINUE
  END


! Lung Tissue and Tissue Blood
        ALng = INTVC(RALng, IALng)                                   ! Amount in lung tissue (mg)
     ALngBld = INTVC(RALngBld, IALngBld)                             ! Amount in lung blood (mg)

! Amount Metabolized in Lung (mg)
       AMetLng = INTVC(RAMetLng, IAMetLng)                           ! Amount metabolized (mg)

  PROCEDURAL (CLng, CLngBld, RALng, RALngBld, RAMetLng = QLng, VLng, VLngBld, VMaxLng, CArt)
DO 3110 i12=1, NChem
     CLng(i12) = ALng(i12) / VLng
! Concentration in lung tissue (mg/L)
     CLngBld(i12) = ALngBld(i12) / VLngBld
! Concentration in lung blood (mg/L)
     RAMetLng(i12) = (VMaxLng(i12) * (CLng(i12) / PLng(i12))) / (KMLng(i12) + (CLng(i12) / PLng(i12)))
! Saturable rate of metabolism in lung (mg/hr)
     RALng(i12) = (PALng(i12) * (CLngBld(i12) - (CLng(i12) / PLng(i12)))) - RAMetLng(i12)
! Rate of change in lung tissue (mg/hr)
     RALngBld(i12) = (QLng * (CArt(i12) - CLngBld(i12))) + (PALng(i12) * ((CLng(i12) / PLng(i12)) -
CLngBld(i12)))     ! Rate of change in lung blood (mg/hr)
3110: CONTINUE
  END


! Rapidly Perfused Tissue and Tissue Blood
        ARap = INTVC(RARap, IARap)                                        ! Amount in rapidly perfused tissue
(mg)
     ARapBld = INTVC(RARapBld, IARapBld)                                  ! Amount in rapidly perfused tissue
blood (mg)

  PROCEDURAL (CRap, CRapBld, RARap, RARapBld = QRap, VRap, VRapBld, CArt)
DO 3120 i13=1, NChem
     CRap(i13) = ARap(i13) / VRap
! Concentration in rapidly perfused tissue (mg/L)
     CRapBld(i13) = ARapBld(i13) / VRapBld
! Concentration in rapidly perfused tissue blood (mg/L)
     RARap(i13) = PARap(i13) * (CRapBld(i13) - (CRap(i13) / PRap(i13)))
! Rate of change in rapidly perfused tissue (mg/hr)
     RARapBld(i13) = (QRap *(CArt(i13) - CRapBld(i13)))+(PARap(i13) * ((CRap(i13) / PRap(i13)) - CRap-
Bld(i13)))   ! Rate of change in rapidly perfused tissue blood (mg/hr)
3120: CONTINUE
  END


! Slowly Perfused Tissue and Tissue Blood
```

```
        ASlw = INTVC(RASlw, IASlw)                                        ! Amount in slowly perfused tissue
(mg)
     ASlwBld = INTVC(RASlwBld, IASlwBld)                                  ! Amount in slowly perfused tissue
blood (mg)

  PROCEDURAL (CSlw, CSlwBld, RASlw, RASlwBld = QSlw, VSlw, VSlwBld, CArt)
DO 3130 i14=1, NChem
     CSlw(i14) = ASlw(i14) / VSlw
! Concentration in slowly perfused tissue (mg/L)
     CSlwBld(i14) = ASlwBld(i14) / VSlwBld
! Concentration in slowly perfused tissue blood (mg/L)
     RASlw(i14) = PASlw(i14) * (CSlwBld(i14) - (CSlw(i14) / PSlw(i14)))
! Rate of change in slowly perfused tissue (mg/hr)
     RASlwBld(i14) = (QSlw * (CArt(i14) - CSlwBld(i14))) + (PASlw(i14) * ((CSlw(i14) / PSlw(i14)) -
CSlwBld(i14)))     ! Rate of change in slowly perfused tissue blood (mg/hr)
3130: CONTINUE
  END


! Amount in Urine (mg)
        AUrn = INTVC(RAUrn, IAUrn)

! Rate of Excretion (mg/hr)
  PROCEDURAL (RAUrn = ClUr, CArt)
DO 3140 i15=1, NChem
     RAUrn(i15) = ClUr(i15) * CArt(i15)
3140: CONTINUE
  END


!---------------- CHECK MASS BALANCE ----------------------------------------------------------------------
! Total Amount Inhaled (mg)
     ATotInh = INTVC(RATotInh, IATotInh)


! Total Amount Injected (mg)
      ATotIV = INTVC(IVR, IATotIV)


! Total Amount Drunk (mg)
   ATotDrink = INTVC(Drink, IATotDrink)


! Calculating Total Brain Concentration (mg/kg), Total Dose (mg), Total Amount in Body (mg) and Mass Balance
(mg)
  PROCEDURAL (RATotInh, TDose, AmtBody, MassBal = QAlv, TotDose, CInh)
DO 3150 i16=1, NChem
     RATotInh(i16) = QAlv * CInh(i16)
     TDose(i16) = ATotInh(i16) + ATotIV(i16) + TotDose(i16) + ATotDrink(i16)
     AmtBody(i16) = AArt(i16) + ABrnStm(i16) + ABrnStmBld(i16) + ABrnTL(i16) + ABrnTLBld(i16) + ABrn(i16) +
ABrnBld(i16) &
                & + ACoc(i16) + ACocBld(i16) + AFat(i16) + AFatBld(i16) + ALiv(i16) + ALivBld(i16) +
ALng(i16) + ALngBld(i16) &
                & + ARap(i16) + ARapBld(i16) + ASlw(i16) + ASlwBld(i16) + AExh(i16) + AUrn(i16) + AMet1(i16)
+ AMet2(i16) &
                & + AMetLng(i16) + AExc(i16) + ADu(i16) + ASt(i16)
     MassBal(i16) = TDose(i16) - AmtBody(i16)
3150: CONTINUE
  END

TERMT(T.GT.TStop, 'Simulation Finished')

END                ! End of Derivative
END                ! End of Dynamic
END                ! End of Program
```

3. Utility .m Files

init.m

This file should be set to execute at load-time.

```
prepare T HOURS MINUTES DAYS CART AUCCART CBRNSTM CBRNSTMBLD AUCCBRNSTM CBRNTL CBRNTLBLD
prepare CBRN CBRNBLD AUCCBRN CBRNTOT CBRNTOTBLD CCOC CCOCBLD CFAT CFATBLD CLIV AUCCLIV CLIVBLD
prepare CLNG CLNGBLD CRAP CRAPBLD CSLW CSLWBLD CVEN AEXH CINH CEND AURN TDOSE AMTBODY MASSBAL
```

```
HVDPRN=0;
WESITG=0;
WEDITG=0;
```

ResetDoses.m

```
  ADJFLOWS=1;  ADJVOLS=1;

  for iter = [1 : 6]
      CONC(iter)=0.0;
      IVDOSE(iter)=0.0;
      PDOSE(iter)=0.0;
      PDRINK(iter)=0.0;
  end

  TCHNG=0.0; DAYSWK=1.0; TMAX=24.0; DOSEINT=1000.0;
  TINF=0.2;
  RATS=1.0;
  CC=0; VCHC=9.1; KLCC=0.0;
  CINT=0.01;
```

Rattus.m

```
% Generic rat parameters

% REF1 -- Clewell HJ, III, Gentry PR, Gearhart JM, Covington TR, Banton MI, Andersen ME: Development of a
physiologically
%    based pharmacokinetic model of isopropanol and its metabolite acetone. Toxicol Sci 2001, 63(2):160-172.
% REF2 -- Gjedde et al 1980 Rapid simultan determinat of regional blood flow_blood-brain glucose transfer in
brain of rats
% REF3 -- Robinson et al. 2013 TR
% REF4 -- Sterner et al. 2014 TMB-4 model
% REF5 -- Delp et al. 1991
% REF6 -- AALAS Learning Library, 2005
% REF7 -- Morizuno et al 1968 Cochlear blood vol in Gpig measured w Cr51 label RBC (Hearing Loss reference
file)
% REF8 -- Merrill et al 2008 Improved predictive model 4 decane kinetics across species.pdf
% REF9 -- Robinson and Merrill 2008 Harmonized PBPK mdl 4 nonane as component of jet fuel.pdf
% REF10 -- Brown et al. 1997

% All cochlea parameters are for paired cochlea.


% Values from REF1, unless otherwise stated
  BW=0.25;               % 0.25 from EPA, REF1 = 0.22, REF8&9 = 0.3
  QCC=14.6;
  QPC=24.75;

  QBRNSTMC=0.004;
  QBRNTLC=0.003;        % Calculated using brain volumes & REF2
  QBRNC=0.013;          % Remainder only; Adds up to 0.02 (REF10)
  QCOCC=0.00004;        % REF3
  QFATC=0.07;
  QLIVC=0.183;
  QLNGC=0.021;
  QRAPC=0.536;          % 0.557-Lung
  QSLWC=0.17;           % Includes cochlea

  VALVC=0.007;
  VBRNSTMC=0.001;       % REF5 Table 3
  VBRNTLC=0.001;        % in-house research
  VBRNC=0.004;          % Remainder only; 0.006 - VBRNSTMC - VBRNTLC; 0.006 from REF5 Table 3 (0.0057 in
REF10)
  VCOCMC=0.00007;       % Cochlea (pair) -- THIS ISN'T FRACTIONAL -- is multiplied by BW^0.2348 in model code
  VFATC=0.10;           % 0.0625 in REF10
  VLIVC=0.034;          % 0.0366 in REF10
  VLNGC=0.005;          % REF10
  VRAPC=0.039;          % 0.044-Lung
  VSLWC=0.65;
  VBODYC=0.841;
```

```
  DS=0.15;              % Per REF1: acetone = 0.25, isopropanol = 0.15, typical lipophilics = 0.3, csl de-
fault=0.15

% Blood content values for most tissues from REF5
  VBRNSTMBLDC=0.03;     % Assumed same across the brain
  VBRNTLBLDC=0.03;
  VBRNBLDC=0.03;        % REF4 and REF10
  VCOCBLDC=0.0183;      % Calculated from data in REF7
  VFATBLDC=0.0154;      % Not sure of source but close enough to REF10 (0.02)
  VLIVBLDC=0.21;        % REF4, REF10
  VLNGBLDC=0.36;        % REF10
  VRAPBLDC=0.2075;
  VSLWBLDC=0.0333;
```

Rat_6HCs.m

```
% Sets parameters for rat for all 6 hydrocarbons (HCs)

% Chemical 1 = Toluene
% Chemical 2 = Ethylbenzene
% Chemical 3 = Xylenes
% Chemical 4 = Nonane
% Chemical 5 = Decane
% Chemical 6 = Naphthalene

% REF1 -- Haddad et al 1999 Physiological modeling of the toxicokinetic interactions in a quaternary mixture
of aromatic
%    hydrocarbons.  Toxicol Appl Physiol. 161:249-57.
% REF2 -- Sterner et al 2004 AFRL-HE-WP-TR-2004-0032 Analysis of algorithms predicting blood-air_tissue-blood
PCs fr solvent PCs.pdf
% REF3 -- Schlosser PM, Asgharian BA, Medinsky M. 2010 Chapter 1.04 Inhalation Exposure and Absorption of
Toxicants.
%    In: McQueen CA (ed).  Comprehensive Toxicology. Volume 14.  Elsevier e-book
% REF4 -- Hack Toluene model, Rat_Toluene.M
% REF5 -- Clewell HJ, III, Gentry PR, Gearhart JM, Covington TR, Banton MI, Andersen ME:  Development of a
physiologically
%    based pharmacokinetic model of isopropanol and its metabolite acetone. Toxicol Sci 2001, 63(2):160-172.
% REF6 -- Robinson, P.J. and Merrill, E.A. 2008. A harmonized physiologically based pharmacokinetic model for
nonane as a
%    AFRL-RH-WP-TR-2008-0067, ADA502610.
% REF7 -- Sterner et al 2006 Analysis of algorithms...
% REF8 -- Joshi et al. 2010 PCs 4 nonane_isomers in rat.pdf
% REF9 -- Robinson and Merrill 2008 Harmonized PBPK mdl 4 nonane as component of jet fuel.pdf
% REF10 -- Merrill et al 2008 Improved predictive model 4 decane kinetics across species.pdf
% REF11 -- Buckpitt A, Morin D, Murphy S, Edwards P, Van Winkle L. Kinetics of naphthalene metabolism in tar-
get
%    and non-target tissues of rodents and in nasal and airway microsomes from the Rhesus monkey. Toxicol
Appl
%    Pharmacol. 2013 Jul 15;270(2):97-105.
% REF12 -- Campbell JL, Andersen ME, Clewell HJ. A hybrid CFD-PBPK model for naphthalene in rat and human
with
%    IVIVE for nasal tissue metabolism and cross-species dosimetry. Inhal Toxicol. 2014 May;26(6):333-44.
% REF13 -- Cho TM, Rose RL, Hodgson E. In vitro metabolism of naphthalene by human liver microsomal cyto-
chrome
%    P450 enzymes. Drug Metab Dispos. 2006 Jan;34(1):176-83.
% REF14 -- NIST Chemistry Webbook (https://webbook.nist.gov/cgi/cbook.cgi?Name=naphthalene&Units=SI)
% REF15 -- Schwarz FP, Wasik SP. 1977. A fluorescence method for the measurement of the partition coeffi-
cients of
%    naphthalene, 1-methylnaphthalene, and 1-ethylnaphthalene in water. J. Chem. Eng. Data 22(3):270-273.
% REF16 -- Ruark CD, Hack CE, Robinson PJ, Mahle DA, Gearhart JM. Predicting passive and active tissue:plasma
partition
%    coefficients: interindividual and interspecies variability. J Pharm Sci. 2014 Jul;103(7):2189-2198.
% REF17 -- Sterner 2017 Technical report on JFHL


  MWList=[92.14 106.17 106.17 128.0 142.3 128.1705];
  PBList=[18.0 42.7 46.0 5.2 5.0 571.0];
  PBrnStmList=[2.87 1.93 2.29 0.0 0.0 3.5];
  PBrnTLList=[2.13 1.44 1.61 0.0 0.0 3.5];
  PBrnList=[2.0 1.22 1.38 5.0 10.0 3.5];
  PCoCList=[0.54 0.44 0.47 1.45 2.15 0.47];
  PFatList=[56.7 36.4 40.4 282.0 328.0 49.0];
  PLivList=[4.64 1.96 1.98 8.0 3.0 1.6];
  PLngList=[4.64 1.41 1.98 2.0 3.0 3.5];
  PRapList=[4.64 1.41 1.98 2.0 3.0 3.5];
```

```
  PSlwList=[1.54 0.61 0.91 4.0 0.85 3.5];
  VMaxCList=[3.44 6.39 6.49 0.1 0.005 -1.0];
  KMList=[0.13 1.04 0.45 0.1 0.1 2.18];
  ClUrCList=[0.004 0.04 0.004 0.4 0.004 0.0];

  for iter = [1 : 6]
      MW(iter) = MWList(iter);

      PB(iter) = PBList(iter);
      PBRNSTM(iter) = PBrnStmList(iter);
      PBRNTL(iter) = PBrnTLList(iter);
      PBRN(iter) = PBrnList(iter);
      PCOC(iter) = PCoCList(iter);
      PFAT(iter) = PFatList(iter);
      PLIV(iter) = PLivList(iter);
      PLNG(iter) = PLngList(iter);
      PRAP(iter) = PRapList(iter);
      PSLW(iter) = PSlwList(iter);

% If PA = 10000.0, essentially equivalent to flow limited
      PABRNSTM(iter) = 10000.0;
      PABRNTL(iter) = 10000.0;
      PABRN(iter) = 10000.0;
      PACOC(iter) = 10000.0;
      PAFAT(iter) = 10000.0;
      PALIV(iter) = 10000.0;
      PALNG(iter) = 10000.0;
      PARAP(iter) = 10000.0;
      PASLW(iter) = 10000.0;

      VMAXC(iter) = VMaxCList(iter);
      KFC(iter) = 0.0;                % Not expecting first order metabolism for these compounds
      KM(iter) = KMList(iter);
      VMAXLNGC(iter) = 0.0;           % Assumed all metabolism in liver except for Naphthalene (VMAXLNGC in
NaDORT model was not allometrically scaled -- REF11, added 2 enzyme values)
      KMLNG(iter) = 1.0;              % KMLNG values don't matter when VMAXLNGC values were zero so set to
1.0 to avoid division by zero

      CLURC(iter) = ClUrCList(iter);
      SCRUB(iter) = 0.0;              % Fraction of concentration in air that is scrubbed out (not ab-
sorbed), 0=no scrubbing

      KAD(iter) = 0.0;
      KAS(iter) = 0.0;
      KTD(iter) = 0.0;
      KTSD(iter) = 0.0;
  end

  PBRNSTM(4) = (2.9/1.7)*PBRN(4);
  PBRNSTM(5) = (1.56/1.75)*PBRN(5);
  PBRNTL(4) = (2.6/1.7)*PBRN(4);
  PBRNTL(5) = (1.16/1.75)*PBRN(5);


% If PA = 10000.0, essentially equivalent to flow limited
  PABRNSTM(4)=0.5;  PABRNSTM(5)=0.005;
  PABRNTL(4)=0.5;   PABRNTL(5)=0.005;
  PABRN(4)=0.5;     PABRN(5)=0.005;
  PACOC(4)=1.0;     PACOC(5)=1.0;
  PAFAT(4)=0.5;     PAFAT(5)=0.07;      PAFAT(6)=0.2*(BW^0.75);
  PALIV(4)=0.07;    PALIV(5)=0.15;
  PALNG(4)=1.0;     PALNG(5)=0.005;
  PARAP(4)=1.0;     PARAP(5)=0.005;
  PASLW(4)=0.5;     PASLW(5)=0.14;      PASLW(6) = 2.0*(BW^0.75);


% Assumed all metabolism in liver except for Naphthalene (VMAXLNGC in NaDORT model was not allometrically
scaled -- REF11, added 2 enzyme values)
  VMAXC(6) = (8.28*BW)/(BW^0.75);
  VMAXLNGC(6) = (0.45*BW)/(BW^0.75);


% KMLNG values don't matter when VMAXLNGC values were zero so set to 1.0 to avoid division by zero
  KMLNG(6) = 2.18;
```

```
% Fraction of concentration in air that is scrubbed out (not absorbed), 0=no scrubbing
  SCRUB(4)=0.4;  SCRUB(5)=0.7;
```

4. Validation Figures .m Files

These .m files were used to produce Figures 2-4 in the main article and all figures in Appendix A.

C1_Lam.m

```
% Lam, C.W., Galen, T.J., Boyd, J.F. and Pierson, D.L. 1990. Mechanism of transport and distribution
%    of organic solvents in blood. Toxicol.Appl.Pharmacol. 104(1): 117-129.

% Male Sprague-Dawley rats, BW about 300 g.
% 2-hour whole-body inhalation of 488 +/- 24 ppm toluene.
% n = 5 rats, put in 10 minutes apart.
% 30 L closed chamber with 5 rats, but CONC is maintained at 488 ppm, rather than depleted, so run as open
chamber.
% Data from Plasma+RBC column, Table 1, page 121


% T (hr), CVEN (mg/L)
Lam = [
  2.0   10.90
  2.0   15.03
  2.0   14.98
  2.0   15.02
  2.0   15.09];


  ResetDoses
  Rattus
  Rat_6HCs

% Study specific values as found in Hack tolune model, Lam.M
  BW=0.3; CONC(1)=488.0; TCHNG=2.0; TSTOP=4.0;
%  VMAXC(1)=3.44; KM(1)=0.13;
  start @nocallback
  plotcven = plot (0, Lam(:,1), Lam(:,2), '+b', _t, _cven(:,1), '-b');


set @preference=BackslashEscapes
    pltscript(plotcven, "Chart.Header.Text = \"Lam et al. (1990) Toluene Inhalation Study\";")
    pltscript(plotcven, "Chart.SubHeader.Text = \"488 ppm Inhalation for 2 Hours\";")
    pltscript(plotcven, "Chart.SubHeader.Visible=true;")
    pltscript(plotcven, "Chart.Axes.Left.Title.Text=\"Toluene in Venous Blood [mg/L]\";")
    pltscript(plotcven, "Chart.Axes.Bottom.Title.Text=\"Hours\";")
    pltscript(plotcven, "Chart.Series[0].Title=\"488 ppm\";")
    pltscript(plotcven, "Chart.Series[1].Title=\"Simulation 488 ppm\";")
set @preference=NoBackslashEscapes
```

C1_Romer.m

```
% Romer, K.G., Federsel, R.J. and Freundt, K.J. 1986. Rise of inhaled toluene, ethyl benzene,
%    m-xylene, or mesitylene in rat blood after treatment with ethanol. Bull Environ Contam
%    Toxicol 37(6): 874-876.

% Adult female SPF Sprague-Dawley rats (200 - 220 g BW)
% Groups of 3 rats exposed in a 20 L glass chamber, air flow 1.25 L/min, for 2 hr.  20 L closed chamber with
3 rats,
%    but CONC is maintained at 220 ppm, rather than depleted, so run as open chamber.
% Aromatic conc varied < 5%, delivered via evaporator.  Sham-treated groups given 5 mL/kg physiological sa-
line.
% Exposure conc monitored via GC using 100 uL air samples.
% Blood (0.02 mL) collected from retro-orbital plexus.  Blood conc of aromatics determined by GC.


% Table 1
% meanblood = (56.8e-6 * 92.14 * 1000)=5.23, mol/L conversion to mg/L, MW=92.14
% semblood = (2.1e-6*92.14*1000)=0.19, mol/L conversion to mg/L, MW=92.14
% sem range = 5.04 - 5.43
```

```
% T(hr), CVEN(mg/L)
Romer = [
  2.0   5.23
  2.0   5.04
  2.0   5.43];


  ResetDoses
  Rattus
  Rat_6HCs

  BW=0.210; CONC(1)=220.0; TCHNG=2.0; TSTOP=3.0;
%  VMAXC(1)=3.44; KM(1)=0.13;
  start @nocallback
  plotcven = plot (0, Romer(:,1), Romer(:,2), '+b', _t, _cven(:,1), '-b');

set @preference=BackslashEscapes
    pltscript(plotcven, "Chart.Header.Text = \"Romer et al. (1986) Toluene Inhalation Study\";")
    pltscript(plotcven, "Chart.SubHeader.Text = \"220 ppm Inhalation for 2 Hours\";")
    pltscript(plotcven, "Chart.SubHeader.Visible=true;")
    pltscript(plotcven, "Chart.Axes.Left.Title.Text=\"Toluene in Venous Blood [mg/L]\";")
    pltscript(plotcven, "Chart.Axes.Bottom.Title.Text=\"Hours\";")
    pltscript(plotcven, "Chart.Series[0].Title=\"220 ppm mean value\";")
    pltscript(plotcven, "Chart.Series[1].Title=\"Simulation 220 ppm\";")
set @preference=NoBackslashEscapes
```

C1to3_Haddad.m

```
% Combines C1_Haddad.m, C2_Haddad.m and C3_Haddad.m

% Haddad, S., Tardif, R., Charest-Tardif, G. and Krishnan, K. 1999. Physiological modeling
%    of the toxicokinetic interactions in a quaternary mixture of aromatic hydrocarbons.
%    Toxicol.Appl.Pharmacol. 161(3): 249-257.


% T, CVEN
Had50Toluene = [
  4.0   0.472
  4.0   0.546
  4.5   0.210
  4.5   0.281
  4.5   0.354
  5.0   0.093
  5.0   0.136
  5.0   0.182
  5.5   0.083
  5.5   0.133
  5.5   0.108
  6.0   0.045
  6.0   0.059
  6.0   0.072];


% T, CVEN (mean, +SD, -SD)
Had50Ethylben = [
  4.1   0.66
  4.5   0.46
  5.0   0.28
  5.5   0.17
  6.0   0.13
  4.1   0.78
  4.5   0.53
  5.0   0.32
  5.5   0.20
  6.0   0.17
  4.1   0.59
  4.5   0.33
  5.0   0.22
  5.5   0.15
  6.0   0.09];


% T, CVEN (mean, +SD, -SD)
Had50Xylene = [
```

```
  4.1   0.49
  4.5   0.32
  5.0   0.24
  5.5   0.17
  6.0   0.12
  4.1   0.55
  4.5   0.40
  5.0   0.29
  5.5   0.21
  6.0   0.15
  4.1   0.43
  4.5   0.23
  5.0   0.20
  5.5   0.14
  6.0   0.09];

% T, CVEN
Had100Toluene = [
  4.0   0.751
  4.0   1.033
  4.0   1.464
  4.5   0.547
  4.5   0.632
  4.5   0.798
  5.0   0.281
  5.0   0.345
  5.0   0.435
  5.5   0.199
  5.5   0.290
  5.5   0.230
  6.0   0.112
  6.0   0.133
  6.0   0.173];

% T, CVEN
Had100Ethylben = [
  4.1   1.52
  4.5   1.02
  5.0   0.70
  5.5   0.45
  6.0   0.31
  4.1   1.68
  4.5   1.15
  5.0   0.89
  5.5   0.58
  6.0   0.36
  4.1   1.36
  4.5   0.94
  5.0   0.57
  5.5   0.38
  6.0   0.28];

% T, CVEN
Had100Xylene = [
  4.1   1.20
  4.5   0.86
  5.0   0.50
  5.5   0.37
  6.0   0.24
  4.1   1.46
  4.5   1.02
  5.0   0.62
  5.5   0.45
  6.0   0.33
  4.1   1.10
  4.5   0.73
  5.0   0.44
  5.5   0.33
  6.0   0.17];

% T, CVEN
Had200Toluene = [
```

```
  4.0   3.205
  4.0   4.042
  4.0   5.099
  4.5   2.334
  4.5   3.120
  4.5   3.714
  5.0   1.559
  5.0   1.856
  5.0   2.274
  5.5   0.826
  5.5   1.352
  5.5   1.072
  6.0   0.602
  6.0   0.716
  6.0   0.804];


% T, CVEN
Had200Ethylben = [
  4.1   5.73
  4.5   3.79
  5.0   2.70
  5.5   1.92
  6.0   1.36
  4.1   6.57
  4.5   4.24
  5.0   3.02
  5.5   2.21
  6.0   1.66
  4.1   5.05
  4.5   3.42
  5.0   2.44
  5.5   1.72
  6.0   1.12];


% T, CVEN
Had200Xylene = [
  4.1   6.26
  4.5   4.13
  5.0   2.83
  5.5   1.97
  6.0   1.33
  4.1   7.60
  4.5   5.68
  5.0   3.93
  5.5   2.83
  6.0   1.97
  4.1   4.96
  4.5   2.80
  5.0   1.77
  5.5   1.12
  6.0   0.71];


  ResetDoses
  Rattus
  Rat_6HCs

% Toluene study specific values as found in Hack tolune model, Haddad.M
  BW=0.235; TCHNG=4.0; TSTOP=8.0;
  CONC(1)=50.0;  CONC(2)=50.0;  CONC(3)=50.0;
  VMAXC(1)=3.44; KM(1)=0.13;
  start @nocallback
  plotcvenT = plot (0, Had50Toluene(:,1), Had50Toluene(:,2), '+b', _t, _cven(:,1), '-b');
  plotcvenE = plot (0, Had50Ethylben(:,1), Had50Ethylben(:,2), '+b', _t, _cven(:,2), '-b');
  plotcvenX = plot (0, Had50Xylene(:,1), Had50Xylene(:,2), '+b', _t, _cven(:,3), '-b');

  CONC(1)=100.0;  CONC(2)=100.0;  CONC(3)=100.0;
  start @nocallback
  plot (plotcvenT, 1, Had100Toluene(:,1), Had100Toluene(:,2), '+k', _t, _cven(:,1), '-k');
  plot (plotcvenE, 1, Had100Ethylben(:,1), Had100Ethylben(:,2), '+k', _t, _cven(:,2), '-k');
  plot (plotcvenX, 1, Had100Xylene(:,1), Had100Xylene(:,2), '+k', _t, _cven(:,3), '-k');

  CONC(1)=200.0;  CONC(2)=200.0;  CONC(3)=200.0;
  start @nocallback
```

```
  plot (plotcvenT, 1, Had200Toluene(:,1), Had200Toluene(:,2), '+g', _t, _cven(:,1), '-g');
  plot (plotcvenE, 1, Had200Ethylben(:,1), Had200Ethylben(:,2), '+g', _t, _cven(:,2), '-g');
  plot (plotcvenX, 1, Had200Xylene(:,1), Had200Xylene(:,2), '+g', _t, _cven(:,3), '-g');


set @preference=BackslashEscapes
    pltscript(plotcvenT, "Chart.Header.Text = \"Haddad et al. (1999) Toluene Inhalation Study\";")
    pltscript(plotcvenT, "Chart.SubHeader.Text = \"50, 100, 200 ppm Inhalation for 4 Hours\";")
    pltscript(plotcvenT, "Chart.SubHeader.Visible=true;")
    pltscript(plotcvenT, "Chart.Axes.Left.Title.Text=\"Toluene in Venous Blood [mg/L]\";")
    pltscript(plotcvenT, "Chart.Axes.Bottom.Title.Text=\"Hours\";")
    pltscript(plotcvenT, "Chart.Series[0].Title=\"50 ppm\";")
    pltscript(plotcvenT, "Chart.Series[1].Title=\"Simulation 50 ppm\";")
    pltscript(plotcvenT, "Chart.Series[2].Title=\"100 ppm\";")
    pltscript(plotcvenT, "Chart.Series[3].Title=\"Simulation 100 ppm\";")
    pltscript(plotcvenT, "Chart.Series[4].Title=\"200 ppm\";")
    pltscript(plotcvenT, "Chart.Series[5].Title=\"Simulation 200 ppm\";")

    pltscript(plotcvenE, "Chart.Header.Text = \"Haddad et al. (1999) Ethylbenzene Inhalation Study\";")
    pltscript(plotcvenE, "Chart.SubHeader.Text = \"50, 100, 200 ppm Inhalation for 4 Hours\";")
    pltscript(plotcvenE, "Chart.SubHeader.Visible=true;")
    pltscript(plotcvenE, "Chart.Axes.Left.Title.Text=\"Ethylbenzene in Venous Blood [mg/L]\";")
    pltscript(plotcvenE, "Chart.Axes.Bottom.Title.Text=\"Hours\";")
    pltscript(plotcvenE, "Chart.Series[0].Title=\"50 ppm\";")
    pltscript(plotcvenE, "Chart.Series[1].Title=\"Simulation 50 ppm\";")
    pltscript(plotcvenE, "Chart.Series[2].Title=\"100 ppm\";")
    pltscript(plotcvenE, "Chart.Series[3].Title=\"Simulation 100 ppm\";")
    pltscript(plotcvenE, "Chart.Series[4].Title=\"200 ppm\";")
    pltscript(plotcvenE, "Chart.Series[5].Title=\"Simulation 200 ppm\";")

    pltscript(plotcvenX, "Chart.Header.Text = \"Haddad et al. (1999) m-Xylene Inhalation Study\";")
    pltscript(plotcvenX, "Chart.SubHeader.Text = \"50, 100, 200 ppm Inhalation for 4 Hours\";")
    pltscript(plotcvenX, "Chart.SubHeader.Visible=true;")
    pltscript(plotcvenX, "Chart.Axes.Left.Title.Text=\"m-Xylene in Venous Blood [mg/L]\";")
    pltscript(plotcvenX, "Chart.Axes.Bottom.Title.Text=\"Hours\";")
    pltscript(plotcvenX, "Chart.Series[0].Title=\"50 ppm\";")
    pltscript(plotcvenX, "Chart.Series[1].Title=\"Simulation 50 ppm\";")
    pltscript(plotcvenX, "Chart.Series[2].Title=\"100 ppm\";")
    pltscript(plotcvenX, "Chart.Series[3].Title=\"Simulation 100 ppm\";")
    pltscript(plotcvenX, "Chart.Series[4].Title=\"200 ppm\";")
    pltscript(plotcvenX, "Chart.Series[5].Title=\"Simulation 200 ppm\";")
set @preference=NoBackslashEscapes
```

C1to3_Tardif.m

```
% Tardif, R., Charest-Tardif, G. and Brodeur, J. 1996. Comparison of the influence
%    of binary mixtures versus a ternary mixture of inhaled aromatic hydrocarbons
%    on their blood kinetics in the rat. Arch Toxicol 70(7): 405-413.

% Adult male SD rats (235-245 g)


% T, CVEN from Figure 1, mean followed by +SD then -SD
Tar100Toluene = [
  4.1   1.14
  4.5   0.69
  5.0   0.36
  5.5   0.23
  6.0   0.13
  4.1   1.53
  4.5   0.82
  5.0   0.44
  5.5   0.29
  6.0   0.19
  4.1   0.76
  4.5   0.55
  5.0   0.27
  5.5   0.17
  6.0   0.08];


% T, CVEN from Figure 1, mean followed by +SD then -SD
Tar100Ethylben = [
  4.1   1.92
```

```
  4.5   1.27
  5.0   0.86
  5.5   0.53
  6.0   0.35
  4.1   2.11
  4.5   1.42
  5.0   1.08
  5.5   0.66
  6.0   0.41
  4.1   1.79
  4.5   1.14
  5.0   0.63
  5.5   0.43
  6.0   0.29];

% T, CVEN from Figure 1, mean followed by +SD then -SD
Tar100Xylene = [
  4.1   1.26
  4.5   0.86
  5.0   0.51
  5.5   0.37
  6.0   0.24
  4.1   1.44
  4.5   1.01
  5.0   0.62
  5.5   0.43
  6.0   0.30
  4.1   1.09
  4.5   0.71
  5.0   0.43
  5.5   0.31
  6.0   0.15];

% T, CVEN from Figure 2, mean followed by +SD then -SD
Tar200Toluene = [
  4.1   4.34
  4.5   3.17
  5.0   1.96
  5.5   1.19
  6.0   0.79
  4.1   5.29
  4.5   3.94
  5.0   2.31
  5.5   1.42
  6.0   0.89
  4.1   3.37
  4.5   2.47
  5.0   1.66
  5.5   0.93
  6.0   0.64];

% T, CVEN from Figure 4, mean followed by +SD then -SD
Tar200Ethylben = [
  4.1   7.67
  4.5   4.96
  5.0   3.47
  5.5   2.50
  6.0   1.71
  4.1   8.22
  4.5   5.51
  5.0   3.82
  5.5   2.78
  6.0   2.01
  4.1   6.92
  4.5   4.42
  5.0   3.25
  5.5   2.20
  6.0   1.39];

% T, CVEN from Figure 3, mean followed by +SD then -SD
Tar200Xylene = [
  4.1   6.32
```

```
  4.5   4.26
  5.0   2.83
  5.5   1.99
  6.0   1.37
  4.1   7.80
  4.5   5.74
  5.0   3.95
  5.5   2.83
  6.0   2.04
  4.1   4.89
  4.5   2.83
  5.0   1.75
  5.5   1.12
  6.0   0.74];


  ResetDoses
  Rattus
  Rat_6HCs

  BW=0.240; TCHNG=4.0; TSTOP=6.0;
  CONC(1)=100.0;  CONC(2)=100.0;  CONC(3)=100.0;
  start @nocallback
  plotcvenT = plot (0, Tar100Toluene(:,1), Tar100Toluene(:,2), '+b', _t, _cven(:,1), '-b');
  plotcvenE = plot (0, Tar100Ethylben(:,1), Tar100Ethylben(:,2), '+b', _t, _cven(:,2), '-b');
  plotcvenX = plot (0, Tar100Xylene(:,1), Tar100Xylene(:,2), '+b', _t, _cven(:,3), '-b');

  CONC(1)=200.0;  CONC(2)=200.0;  CONC(3)=200.0;
  start @nocallback
  plotcvenT = plot (plotcvenT, 1, Tar200Toluene(:,1), Tar200Toluene(:,2), '+k', _t, _cven(:,1), '-k');
  plotcvenE = plot (plotcvenE, 1, Tar200Ethylben(:,1), Tar200Ethylben(:,2), '+k', _t, _cven(:,2), '-k');
  plotcvenX = plot (plotcvenX, 1, Tar200Xylene(:,1), Tar200Xylene(:,2), '+k', _t, _cven(:,3), '-k');

set @preference=BackslashEscapes
    pltscript(plotcvenT, "Chart.Header.Text = \"Tardif et al. (1996) Toluene Inhalation Study\";")
    pltscript(plotcvenT, "Chart.SubHeader.Text = \"100, 200 ppm Inhalation for 4 Hours\";")
    pltscript(plotcvenT, "Chart.SubHeader.Visible=true;")
%    pltscript(plotcvenT, "Chart.Axes.Left.Automatic=false;")
%    pltscript(plotcvenT, "Chart.Axes.Left.Minimum=0.0;")
%    pltscript(plotcvenT, "Chart.Axes.Left.Maximum=2.0;")
    pltscript(plotcvenT, "Chart.Axes.Left.Title.Text=\"Toluene in Venous Blood [mg/L]\";")
%    pltscript(plotcvenT, "Chart.Axes.Bottom.Automatic=false;")
%    pltscript(plotcvenT, "Chart.Axes.Bottom.Minimum=0.0;")
%    pltscript(plotcvenT, "Chart.Axes.Bottom.Maximum=60.0;")
    pltscript(plotcvenT, "Chart.Axes.Bottom.Title.Text=\"Hours\";")
    pltscript(plotcvenT, "Chart.Series[0].Title=\"100 ppm\";")
    pltscript(plotcvenT, "Chart.Series[1].Title=\"Simulation 100 ppm\";")
    pltscript(plotcvenT, "Chart.Series[2].Title=\"200 ppm\";")
    pltscript(plotcvenT, "Chart.Series[3].Title=\"Simulation 200 ppm\";")

    pltscript(plotcvenE, "Chart.Header.Text = \"Tardif et al. (1996) Ethylbenzene Inhalation Study\";")
    pltscript(plotcvenE, "Chart.SubHeader.Text = \"100, 200 ppm Inhalation for 4 Hours\";")
    pltscript(plotcvenE, "Chart.SubHeader.Visible=true;")
%    pltscript(plotcvenE, "Chart.Axes.Left.Automatic=false;")
%    pltscript(plotcvenE, "Chart.Axes.Left.Minimum=0.0;")
%    pltscript(plotcvenE, "Chart.Axes.Left.Maximum=2.0;")
    pltscript(plotcvenE, "Chart.Axes.Left.Title.Text=\"Ethylbenzene in Venous Blood [mg/L]\";")
%    pltscript(plotcvenE, "Chart.Axes.Bottom.Automatic=false;")
%    pltscript(plotcvenE, "Chart.Axes.Bottom.Minimum=0.0;")
%    pltscript(plotcvenE, "Chart.Axes.Bottom.Maximum=60.0;")
    pltscript(plotcvenE, "Chart.Axes.Bottom.Title.Text=\"Hours\";")
    pltscript(plotcvenE, "Chart.Series[0].Title=\"100 ppm\";")
    pltscript(plotcvenE, "Chart.Series[1].Title=\"Simulation 100 ppm\";")
    pltscript(plotcvenE, "Chart.Series[2].Title=\"200 ppm\";")
    pltscript(plotcvenE, "Chart.Series[3].Title=\"Simulation 200 ppm\";")

    pltscript(plotcvenX, "Chart.Header.Text = \"Tardif et al. (1996) m-Xylene Inhalation Study\";")
    pltscript(plotcvenX, "Chart.SubHeader.Text = \"100, 200 ppm Inhalation for 4 Hours\";")
    pltscript(plotcvenX, "Chart.SubHeader.Visible=true;")
%    pltscript(plotcvenX, "Chart.Axes.Left.Automatic=false;")
%    pltscript(plotcvenX, "Chart.Axes.Left.Minimum=0.0;")
%    pltscript(plotcvenX, "Chart.Axes.Left.Maximum=2.0;")
    pltscript(plotcvenX, "Chart.Axes.Left.Title.Text=\"Xylene in Venous Blood [mg/L]\";")
%    pltscript(plotcvenX, "Chart.Axes.Bottom.Automatic=false;")
%    pltscript(plotcvenX, "Chart.Axes.Bottom.Minimum=0.0;")
%    pltscript(plotcvenX, "Chart.Axes.Bottom.Maximum=60.0;")
```

```
    pltscript(plotcvenX, "Chart.Axes.Bottom.Title.Text=\"Hours\";")
    pltscript(plotcvenX, "Chart.Series[0].Title=\"100 ppm\";")
    pltscript(plotcvenX, "Chart.Series[1].Title=\"Simulation 100 ppm\";")
    pltscript(plotcvenX, "Chart.Series[2].Title=\"200 ppm\";")
    pltscript(plotcvenX, "Chart.Series[3].Title=\"Simulation 200 ppm\";")
set @preference=NoBackslashEscapes
```

C4_Robinson.m

```
% Robinson, P.J. and Merrill, E.A. 2008. A harmonized physiologically based pharmacokinetic model
%     for nonane as a component of jet fuel. Wright-Patterson AFB, OH: Air Force Research
%     Laboratory, Applied Biotechnology Branch. AFRL-RH-WP-TR-2008-0067, ADA502610.


% (t, cven, cfat, cliv, cslw)
Inh90d100 = [
  0.5    0.372     NaN    NaN    NaN
  0.5    0.427     NaN    NaN    NaN
  0.5    0.482     NaN    NaN    NaN
  1.0    0.370     NaN    NaN    NaN
  1.0    0.523     NaN    NaN    NaN
  1.0    0.676     NaN    NaN    NaN
  2.0    0.306     NaN    NaN    NaN
  2.0    0.516     NaN    NaN    NaN
  2.0    0.726     NaN    NaN    NaN
  3.0    0.496     NaN    NaN    NaN
  3.0    0.676     NaN    NaN    NaN
  3.0    0.856     NaN    NaN    NaN
  4.0    0.808    13.93   1.44   1.0
  4.0    0.891    27.24   3.83   2.76
  4.0    0.974    40.55   6.22   4.52
  4.08   0.292     NaN    NaN    NaN
  4.08   0.43      NaN    NaN    NaN
  4.08   0.568     NaN    NaN    NaN
  4.16   0.2251    NaN    NaN    NaN
  4.16   0.362     NaN    NaN    NaN
  4.16   0.498     NaN    NaN    NaN
  4.25   0.191     NaN    NaN    NaN
  4.25   0.304     NaN    NaN    NaN
  4.25   0.417     NaN    NaN    NaN
  4.5    0.097     NaN    NaN    NaN
  4.5    0.205     NaN    NaN    NaN
  4.5    0.313     NaN    NaN    NaN
  5.0    0.053     NaN    NaN    NaN
  5.0    0.114     NaN    NaN    NaN
  5.0    0.175     NaN    NaN    NaN
  6.0    0.004     NaN    NaN    NaN
  6.0    0.037     NaN    NaN    NaN
  6.0    0.07      NaN    NaN    NaN
  7.0    0.003     NaN    NaN    NaN
  7.0    0.03      NaN    NaN    NaN
  7.0    0.057     NaN    NaN    NaN
  8.0    0.0       8.29   0.0    0.0
  8.0    0.019    18.65   0.05   0.7
  8.0    0.042    29.01   0.22   1.65];


% (t, CV1, CF1, CL1, CS1)
Inh90d500 = [
  0.6    1.311     NaN     NaN     NaN
  0.6    2.051     NaN     NaN     NaN
  0.6    2.791     NaN     NaN     NaN
  1.1    1.406     NaN     NaN     NaN
  1.1    2.263     NaN     NaN     NaN
  1.1    3.12      NaN     NaN     NaN
  2.1    2.934     NaN     NaN     NaN
  2.1    3.736     NaN     NaN     NaN
  2.1    4.538     NaN     NaN     NaN
  3.1    2.201     NaN     NaN     NaN
  3.1    3.635     NaN     NaN     NaN
  3.1    5.069     NaN     NaN     NaN
  4.1    3.504    66.01   25.03   11.71
  4.1    4.43    131.11   39.63   17.33
  4.1    5.356   196.21   54.23   22.95
```

```
  4.18   3.0       NaN     NaN     NaN
  4.18   3.75      NaN     NaN     NaN
  4.18   4.5       NaN     NaN     NaN
  4.26   1.902     NaN     NaN     NaN
  4.26   2.785     NaN     NaN     NaN
  4.26   3.668     NaN     NaN     NaN
  4.35   1.322     NaN     NaN     NaN
  4.35   1.953     NaN     NaN     NaN
  4.35   2.584     NaN     NaN     NaN
  4.6    1.34      NaN     NaN     NaN
  4.6    1.768     NaN     NaN     NaN
  4.6    2.196     NaN     NaN     NaN
  5.1    0.811     NaN     NaN     NaN
  5.1    1.184     NaN     NaN     NaN
  5.1    1.557     NaN     NaN     NaN
  6.1    0.218     NaN     NaN     NaN
  6.1    0.41      NaN     NaN     NaN
  6.1    0.602     NaN     NaN     NaN
  7.1    0.195     NaN     NaN     NaN
  7.1    0.365     NaN     NaN     NaN
  7.1    0.535     NaN     NaN     NaN
  8.1    0.152    52.64    0.34    0.0
  8.1    0.251    82.2     0.48    4.96
  8.1    0.35    111.76    0.62   10.08];

% (t, CV1, CF1, CL1, CS1)
Inh90d1000 = [
  0.7     1.682     NaN      NaN     NaN
  0.7     6.933     NaN      NaN     NaN
  0.7    12.184     NaN      NaN     NaN
  1.2     3.385     NaN      NaN     NaN
  1.2     6.95      NaN      NaN     NaN
  1.2    10.515     NaN      NaN     NaN
  2.2     9.405     NaN      NaN     NaN
  2.2    15.211     NaN      NaN     NaN
  2.2    21.017     NaN      NaN     NaN
  3.2     7.789     NaN      NaN     NaN
  3.2    16.928     NaN      NaN     NaN
  3.2    26.067     NaN      NaN     NaN
  4.2     4.724    66.86    47.91   23.61
  4.2    16.467   360.28    99.9    45.0
  4.2    28.21    653.7    151.91   66.39
  4.28    6.904     NaN      NaN     NaN
  4.28   13.625     NaN      NaN     NaN
  4.28   20.346     NaN      NaN     NaN
  4.36    2.902     NaN      NaN     NaN
  4.36   10.88      NaN      NaN     NaN
  4.36   18.858     NaN      NaN     NaN
  4.45    3.02      NaN      NaN     NaN
  4.45    9.281     NaN      NaN     NaN
  4.45   15.542     NaN      NaN     NaN
  4.7     1.649     NaN      NaN     NaN
  4.7     5.073     NaN      NaN     NaN
  4.7     8.497     NaN      NaN     NaN
  5.2     0.888     NaN      NaN     NaN
  5.2     4.403     NaN      NaN     NaN
  5.2     7.918     NaN      NaN     NaN
  6.2     0.638     NaN      NaN     NaN
  6.2     2.378     NaN      NaN     NaN
  6.2     4.118     NaN      NaN     NaN
  7.2     0.514     NaN      NaN     NaN
  7.2     1.483     NaN      NaN     NaN
  7.2     2.452     NaN      NaN     NaN
  8.2     0.247   155.42     2.2     7.07
  8.2     0.687   316.26     7.15   21.11
  8.2     1.127   477.1     12.1    35.15];


  ResetDoses
  Rattus
  Rat_6HCs

% Study specific setting found in C1_In_House_Inhal.M from 4Chem9r5 model
  BW=0.3; CONC(4)=100.0; TCHNG=4.0; TSTOP=10;
  start @nocallback
  plotcven = plot (0, Inh90d100(:,1), Inh90d100(:,2), '+b', _t, _cven(:,4), '-b');
```

```
  plotcfat = plot (0, Inh90d100(:,1), Inh90d100(:,3), '+b', _t, _cfat(:,4), '-b');
  plotcliv = plot (0, Inh90d100(:,1), Inh90d100(:,4), '+b', _t, _cliv(:,4), '-b');
  plotcslw = plot (0, Inh90d100(:,1), Inh90d100(:,5), '+b', _t, _cslw(:,4), '-b');

  CONC(4)=500.0; TCHNG(4)=4.1;
  start @nocallback
  plot (plotcven, 1, Inh90d500(:,1), Inh90d500(:,2), '+k', _t, _cven(:,4), '-k');
  plot (plotcfat, 1, Inh90d500(:,1), Inh90d500(:,3), '+k', _t, _cfat(:,4), '-k');
  plot (plotcliv, 1, Inh90d500(:,1), Inh90d500(:,4), '+k', _t, _cliv(:,4), '-k');
  plot (plotcslw, 1, Inh90d500(:,1), Inh90d500(:,5), '+k', _t, _cslw(:,4), '-k');

  CONC(4)=1000.0; TCHNG(4)=4.2;
  start @nocallback
  plot (plotcven, 1, Inh90d500(:,1), Inh90d1000(:,2), '+g', _t, _cven(:,4), '-g');
  plot (plotcfat, 1, Inh90d500(:,1), Inh90d1000(:,3), '+g', _t, _cfat(:,4), '-g');
  plot (plotcliv, 1, Inh90d500(:,1), Inh90d1000(:,4), '+g', _t, _cliv(:,4), '-g');
  plot (plotcslw, 1, Inh90d500(:,1), Inh90d1000(:,5), '+g', _t, _cslw(:,4), '-g');


set @preference=BackslashEscapes
  pltscript(plotcven, "Chart.Header.Text = \"In-House Nonane Inhalation Study\";")
  pltscript(plotcven, "Chart.SubHeader.Text = \"100, 500, 1000 ppm Inhalation for 4 Hours\";")
  pltscript(plotcven, "Chart.SubHeader.Visible=true;")
  pltscript(plotcven, "Chart.Axes.Left.Title.Text=\"Nonane in Venous Blood [mg/L]\";")
  pltscript(plotcven, "Chart.Axes.Bottom.Title.Text=\"Hours\";")
  pltscript(plotcven, "Chart.Series[0].Title=\"100 ppm\";")
  pltscript(plotcven, "Chart.Series[1].Title=\"Simulation 100 ppm\";")
  pltscript(plotcven, "Chart.Series[2].Title=\"500 ppm\";")
  pltscript(plotcven, "Chart.Series[3].Title=\"Simulation 500 ppm\";")
  pltscript(plotcven, "Chart.Series[4].Title=\"1000 ppm\";")
  pltscript(plotcven, "Chart.Series[5].Title=\"Simulation 1000 ppm\";")

  pltscript(plotcfat, "Chart.Header.Text = \"In-House Nonane Inhalation Study\";")
  pltscript(plotcfat, "Chart.SubHeader.Text = \"100, 500, 1000 ppm Inhalation for 4 Hours\";")
  pltscript(plotcfat, "Chart.SubHeader.Visible=true;")
  pltscript(plotcfat, "Chart.Axes.Left.Title.Text=\"Nonane in Fat [mg/L]\";")
  pltscript(plotcfat, "Chart.Axes.Bottom.Title.Text=\"Hours\";")
  pltscript(plotcfat, "Chart.Series[0].Title=\"100 ppm\";")
  pltscript(plotcfat, "Chart.Series[1].Title=\"Simulation 100 ppm\";")
  pltscript(plotcfat, "Chart.Series[2].Title=\"500 ppm\";")
  pltscript(plotcfat, "Chart.Series[3].Title=\"Simulation 500 ppm\";")
  pltscript(plotcfat, "Chart.Series[4].Title=\"1000 ppm\";")
  pltscript(plotcfat, "Chart.Series[5].Title=\"Simulation 1000 ppm\";")

  pltscript(plotcliv, "Chart.Header.Text = \"In-House Nonane Inhalation Study\";")
  pltscript(plotcliv, "Chart.SubHeader.Text = \"100, 500, 1000 ppm Inhalation for 4 Hours\";")
  pltscript(plotcliv, "Chart.SubHeader.Visible=true;")
  pltscript(plotcliv, "Chart.Axes.Left.Title.Text=\"Nonane in Liver [mg/L]\";")
  pltscript(plotcliv, "Chart.Axes.Bottom.Title.Text=\"Hours\";")
  pltscript(plotcliv, "Chart.Series[0].Title=\"100 ppm\";")
  pltscript(plotcliv, "Chart.Series[1].Title=\"Simulation 100 ppm\";")
  pltscript(plotcliv, "Chart.Series[2].Title=\"500 ppm\";")
  pltscript(plotcliv, "Chart.Series[3].Title=\"Simulation 500 ppm\";")
  pltscript(plotcliv, "Chart.Series[4].Title=\"1000 ppm\";")
  pltscript(plotcliv, "Chart.Series[5].Title=\"Simulation 1000 ppm\";")

  pltscript(plotcslw, "Chart.Header.Text = \"In-House Nonane Inhalation Study\";")
  pltscript(plotcslw, "Chart.SubHeader.Text = \"100, 500, 1000 ppm Inhalation for 4 Hours\";")
  pltscript(plotcslw, "Chart.SubHeader.Visible=true;")
  pltscript(plotcslw, "Chart.Axes.Left.Title.Text=\"Nonane in Slowly Perfused Tissues [mg/L]\";")
  pltscript(plotcslw, "Chart.Axes.Bottom.Title.Text=\"Hours\";")
  pltscript(plotcslw, "Chart.Series[0].Title=\"100 ppm\";")
  pltscript(plotcslw, "Chart.Series[1].Title=\"Simulation 100 ppm\";")
  pltscript(plotcslw, "Chart.Series[2].Title=\"500 ppm\";")
  pltscript(plotcslw, "Chart.Series[3].Title=\"Simulation 500 ppm\";")
  pltscript(plotcslw, "Chart.Series[4].Title=\"1000 ppm\";")
  pltscript(plotcslw, "Chart.Series[5].Title=\"Simulation 1000 ppm\";")
set @preference=NoBackslashEscapes
```

C4to5_Loff99.m

```
% Lof, A., Lam, H.R., Gullstrand, E., Ostergaard, G. and Ladefoged, O. 1999. Distribution of
%    dearomatised white spirit in brain, blood, and fat tissue after repeated exposure of
%    rats. Pharmacol.Toxicol. 85(2): 92-97.
```

```
% Rats dosed with 400 and 800 ppm white spirits
% 3.6% n-nonane = 14.4 and 28.8 ppm nonane, respectively
% 16.6% n-decane = 106.4 and 212 ppm decane, respectively


% (T, CVen, CBrnTot, CFat)
Lof99LoNonane = [
  102.0   0.1    0.73   27.26
  102.0   0.14   1.17   32.0
  102.0   0.06   0.29   22.52
  270.0   0.09   0.65   32.5
  270.0   0.15   0.85   39.7
  270.0   0.03   0.45   25.3
  438.0   0.1    0.54   29.06
  438.0   0.13   0.62   34.41
  438.0   0.07   0.46   23.71
  440.0   0.03   0.24   33.85
  440.0   0.04   0.27   41.22
  440.0   0.02   0.21   26.48
  442.0   0.02   0.19   29.75
  442.0   0.03   0.24   35.48
  442.0   0.01   0.14   24.02
  444.0   0.02   0.14   28.54
  444.0   0.03   0.18   32.46
  444.0   0.01   0.1    24.62
  462.0   0.01   0.18   23.75
  462.0   0.01   0.24   24.58
  462.0   0.01   0.12   22.92];


% (T, CVen, CBrnTot, CFat)
% Additional data are present in the original cmd file, but commented out
Lof99LoDecane = [
  102.0   0.7    3.35   150.3
  102.0   0.86   4.99   178.94
  102.0   0.54   1.71   121.66
  270.0   0.88   2.64   182.8
  270.0   1.1    3.27   222.1
  270.0   0.66   2.01   143.5
  438.0   0.7    2.34   172.1
  438.0   0.81   2.89   198.4
  438.0   0.59   1.79   145.8
  440.0   0.3    1.24   202.8
  440.0   0.35   1.34   256.19
  440.0   0.25   1.14   149.41
  442.0   0.24   0.78   190.8
  442.0   0.29   0.93   218.12
  442.0   0.19   0.63   163.48
  444.0   0.21   0.54   191.7
  444.0   0.25   0.72   228.18
  444.0   0.17   0.36   155.22
  462.0   0.13   0.45   166.5
  462.0   0.16   0.88   187.19
  462.0   0.1    0.02   145.81];


% (T, CVen, CBrnTot, CFat)
Lof99HiNonane = [
  102.0   0.26   1.2     69.82
  102.0   0.41   1.58    81.94
  102.0   0.11   0.82    57.7
  270.0   0.21   1.31    90.39
  270.0   0.31   1.54   111.72
  270.0   0.11   1.08    69.06
  438.0   0.32   1.27    99.51
  438.0   0.44   1.4    127.56
  438.0   0.2    1.14    71.46
  440.0   0.06   0.32    88.96
  440.0   0.08   0.4     101.56
  440.0   0.04   0.24    76.36
  442.0   0.03   0.34    74.76
  442.0   0.04   0.51    82.0
  442.0   0.02   0.17    67.52
  444.0   0.02   0.2     69.3
```

```
  444.0   0.03   0.23    75.36
  444.0   0.01   0.17    63.24
  462.0   0.02   0.18    50.07
  462.0   0.02   0.25    58.5
  462.0   0.02   0.11    41.64];


% (T, CVen, CBrnTot, CFat)
Lof99HiDecane = [
  102.0   2.09   6.08   440.2
  102.0   2.78   7.62   493.34
  102.0   1.4    4.54   387.06
  270.0   1.73   6.24   537.7
  270.0   2.12   6.96   642.8
  270.0   1.34   5.52   432.6
  438.0   2.21   5.95   590.5
  438.0   2.75   7.19   770.5
  438.0   1.67   4.71   410.5
  440.0   0.57   2.91   547.3
  440.0   0.64   3.24   616.49
  440.0   0.5    2.58   478.11
  442.0   0.44   1.96   511.9
  442.0   0.48   2.8    571.78
  442.0   0.4    1.12   452.02
  444.0   0.36   1.14   489.6
  444.0   0.42   1.37   534.2
  444.0   0.3    0.91   445.0
  462.0   0.16   0.57   426.0
  462.0   0.18   0.97   462.76
  462.0   0.14   0.17   389.24];


  ResetDoses
  Rattus
  Rat_6HCs

% Default BW from 4Chem9r5 model; No study specific given
  BW=0.3; TCHNG=6.0; DOSEINT=24.0; DAYSWK=5.0; TMAX=504.0; CINT=0.1; TSTOP=525.0;
  CONC(4)=14.4;  CONC(5)=106.4;
  start @nocallback
  plotcvenN = plot (0, Lof99LoNonane(:,1), Lof99LoNonane(:,2), '+b', _t, _cven(:,4), '-b');
  plotcvenD = plot (0, Lof99LoDecane(:,1), Lof99LoDecane(:,2), '+b', _t, _cven(:,5), '-b');
  plotcbrntotN = plot (0, Lof99LoNonane(:,1), Lof99LoNonane(:,3), '+b', _t, _cbrntot(:,4), '-b');
  plotcbrntotD = plot (0, Lof99LoDecane(:,1), Lof99LoDecane(:,3), '+b', _t, _cbrntot(:,5), '-b');
  plotcfatN = plot (0, Lof99LoNonane(:,1), Lof99LoNonane(:,4), '+b', _t, _cfat(:,4), '-b');
  plotcfatD = plot (0, Lof99LoDecane(:,1), Lof99LoDecane(:,4), '+b', _t, _cfat(:,5), '-b');


  CONC(4)=28.8;  CONC(5)=212.0;
  start @nocallback
  plot (plotcvenN, 1, Lof99HiNonane(:,1), Lof99HiNonane(:,2), '+k', _t, _cven(:,4), '-k');
  plot (plotcvenD, 1, Lof99HiDecane(:,1), Lof99HiDecane(:,2), '+k', _t, _cven(:,5), '-k');
  plot (plotcbrntotN, 1, Lof99HiNonane(:,1), Lof99HiNonane(:,3), '+k', _t, _cbrntot(:,4), '-k');
  plot (plotcbrntotD, 1, Lof99HiDecane(:,1), Lof99HiDecane(:,3), '+k', _t, _cbrntot(:,5), '-k');
  plot (plotcfatN, 1, Lof99HiNonane(:,1), Lof99HiNonane(:,4), '+k', _t, _cfat(:,4), '-k');
  plot (plotcfatD, 1, Lof99HiDecane(:,1), Lof99HiDecane(:,4), '+k', _t, _cfat(:,5), '-k');


set @preference=BackslashEscapes
  pltscript(plotcvenN, "Chart.Header.Text = \"Lof et al. (1999) White Spirits Inhalation Study\";")
  pltscript(plotcvenN, "Chart.SubHeader.Text = \"14.4 and 28.8 ppm for 6 Hours\";")
  pltscript(plotcvenN, "Chart.SubHeader.Visible=true;")
  pltscript(plotcvenN, "Chart.Axes.Left.Title.Text=\"Nonane in Venous Blood [mg/L]\";")
  pltscript(plotcvenN, "Chart.Axes.Bottom.Title.Text=\"Hours\";")
  pltscript(plotcvenN, "Chart.Series[0].Title=\"14.4 ppm\";")
  pltscript(plotcvenN, "Chart.Series[1].Title=\"Simulation 14.4 ppm\";")
  pltscript(plotcvenN, "Chart.Series[2].Title=\"28.8 ppm\";")
  pltscript(plotcvenN, "Chart.Series[3].Title=\"Simulation 28.8 ppm\";")

  pltscript(plotcvenD, "Chart.Header.Text = \"Lof et al. (1999) White Spirits Inhalation Study\";")
  pltscript(plotcvenD, "Chart.SubHeader.Text = \"106.4 and 212 ppm for 6 Hours\";")
  pltscript(plotcvenD, "Chart.SubHeader.Visible=true;")
  pltscript(plotcvenD, "Chart.Axes.Left.Title.Text=\"Decane in Venous Blood [mg/L]\";")
  pltscript(plotcvenD, "Chart.Axes.Bottom.Title.Text=\"Hours\";")
  pltscript(plotcvenD, "Chart.Series[0].Title=\"106.4 ppm\";")
  pltscript(plotcvenD, "Chart.Series[1].Title=\"Simulation 106.4 ppm\";")
```

```
  pltscript(plotcvenD, "Chart.Series[2].Title=\"212 ppm\";")
  pltscript(plotcvenD, "Chart.Series[3].Title=\"Simulation 212 ppm\";")

  pltscript(plotcbrntotN, "Chart.Header.Text = \"Lof et al. (1999) White Spirits Inhalation Study\";")
  pltscript(plotcbrntotN, "Chart.SubHeader.Text = \"14.4 and 28.8 ppm for 6 Hours\";")
  pltscript(plotcbrntotN, "Chart.SubHeader.Visible=true;")
  pltscript(plotcbrntotN, "Chart.Axes.Left.Title.Text=\"Nonane in Total Brain [mg/L]\";")
  pltscript(plotcbrntotN, "Chart.Axes.Bottom.Title.Text=\"Hours\";")
  pltscript(plotcbrntotN, "Chart.Series[0].Title=\"14.4 ppm\";")
  pltscript(plotcbrntotN, "Chart.Series[1].Title=\"Simulation 14.4 ppm\";")
  pltscript(plotcbrntotN, "Chart.Series[2].Title=\"28.8 ppm\";")
  pltscript(plotcbrntotN, "Chart.Series[3].Title=\"Simulation 28.8 ppm\";")

  pltscript(plotcbrntotD, "Chart.Header.Text = \"Lof et al. (1999) White Spirits Inhalation Study\";")
  pltscript(plotcbrntotD, "Chart.SubHeader.Text = \"106.4 and 212 ppm for 6 Hours\";")
  pltscript(plotcbrntotD, "Chart.SubHeader.Visible=true;")
  pltscript(plotcbrntotD, "Chart.Axes.Left.Title.Text=\"Decane in Total Brain [mg/L]\";")
  pltscript(plotcbrntotD, "Chart.Axes.Bottom.Title.Text=\"Hours\";")
  pltscript(plotcbrntotD, "Chart.Series[0].Title=\"106.4 ppm\";")
  pltscript(plotcbrntotD, "Chart.Series[1].Title=\"Simulation 106.4 ppm\";")
  pltscript(plotcbrntotD, "Chart.Series[2].Title=\"212 ppm\";")
  pltscript(plotcbrntotD, "Chart.Series[3].Title=\"Simulation 212 ppm\";")

  pltscript(plotcfatN, "Chart.Header.Text = \"Lof et al. (1999) White Spirits Inhalation Study\";")
  pltscript(plotcfatN, "Chart.SubHeader.Text = \"14.4 and 28.8 ppm for 6 Hours\";")
  pltscript(plotcfatN, "Chart.SubHeader.Visible=true;")
  pltscript(plotcfatN, "Chart.Axes.Left.Title.Text=\"Nonane in Fat [mg/L]\";")
  pltscript(plotcfatN, "Chart.Axes.Bottom.Title.Text=\"Hours\";")
  pltscript(plotcfatN, "Chart.Series[0].Title=\"14.4 ppm\";")
  pltscript(plotcfatN, "Chart.Series[1].Title=\"Simulation 14.4 ppm\";")
  pltscript(plotcfatN, "Chart.Series[2].Title=\"28.8 ppm\";")
  pltscript(plotcfatN, "Chart.Series[3].Title=\"Simulation 28.8 ppm\";")

  pltscript(plotcfatD, "Chart.Header.Text = \"Lof et al. (1999) White Spirits Inhalation Study\";")
  pltscript(plotcfatD, "Chart.SubHeader.Text = \"106.4 and 212 ppm for 6 Hours\";")
  pltscript(plotcfatD, "Chart.SubHeader.Visible=true;")
  pltscript(plotcfatD, "Chart.Axes.Left.Title.Text=\"Decane in Fat [mg/L]\";")
  pltscript(plotcfatD, "Chart.Axes.Bottom.Title.Text=\"Hours\";")
  pltscript(plotcfatD, "Chart.Series[0].Title=\"106.4 ppm\";")
  pltscript(plotcfatD, "Chart.Series[1].Title=\"Simulation 106.4 ppm\";")
  pltscript(plotcfatD, "Chart.Series[2].Title=\"212 ppm\";")
  pltscript(plotcfatD, "Chart.Series[3].Title=\"Simulation 212 ppm\";")
set @preference=NoBackslashEscapes
```

C5_Perleberg04.m

```
% Perleberg, U.R., Keys, D.A. and Fisher, J.W. 2004. Development of a physiologically
%     based pharmacokinetic model for decane, a constituent of Jet Propellent-8.
%     Inhal.Toxicol. 16(11-12): 771-783.


% (1-T, 2-CArt, 3-CLiv, 4-CBrnTot, 5-CRap, 6-CFat, 7-conc-bone-marrow, 8-conc-skin)
Perl1200 = [
   4.08   4.34   33.74   120.38   11.09   104.91   260.89     8.18
   4.08   4.79   37.96   131.21   13.08   122.68   315.37     9.83
   4.08   3.89   29.52   109.55    9.10    87.15   206.42     6.53
   4.50   0.63   17.48   94.85    11.02    60.51   195.57     8.72
   4.50   0.82   23.02   108.64   13.91    81.42   272.81    10.82
   4.50   0.44   11.94    81.07    8.12    39.60   118.33     6.622
   5.00   0.58    5.17    73.29    5.84   112.89     NaN     24.12
   5.00   0.72    6.15    75.55    7.44   142.72     NaN     37.15
   5.00   0.45    4.18    71.04    4.23    83.05     NaN     11.09
   6.00   0.26    1.16    45.37    4.97    88.52   158.30    10.43
   6.00   0.32    1.64    48.94    6.32   107.13   200.46    13.81
   6.00   0.20    0.69    41.80    3.62    69.91   116.14     7.050
   8.00   0.15    0.55    14.76    3.23    94.43     NaN      8.363
   8.00   0.19    0.74    15.76    3.76   118.91     NaN     13.45
   8.00   0.10    0.37    13.76    2.71    69.94     NaN      3.277
  10.00   NaN     NaN      6.90    3.82   101.23    57.139   20.98
  10.00   NaN     NaN      7.77    5.48   123.56    66.751   29.04
  10.00   NaN     NaN      6.02    2.17    78.89    47.528   12.93
  16.00   NaN     NaN      2.09    2.85    74.96     6.140    0.861
  16.00   NaN     NaN      2.28    4.15    83.96     6.911    1.463
  16.00   NaN     NaN      1.90    1.55    65.96     5.370    0.259
```

```
  28.00   NaN     NaN      0.36    2.45    55.34     1.348    1.004
  28.00   NaN     NaN      0.45    3.66    64.11     1.672    1.572
  28.00   NaN     NaN      0.27    1.24    46.57     1.025    0.435];


% (1-T, 2-CArt, 3-CLiv, 4-CBrnTot, 5-CRap, 6-CFat, 7-conc-bone-marrow, 8-conc-skin)
Perl781 = [
  4.00   2.68   34.8   61.1   12.1    51.1   109.64    NaN
  4.00   3.58   45.1   65.9   16.7    72.3   163.83    NaN
  4.00   1.78   24.5   56.2    7.42   29.7    55.469   NaN];


% (1-T, 2-CArt, 3-CLiv, 4-CBrnTot, 5-CRap, 6-CFat, 7-conc-bone-marrow, 8-conc-skin)
Perl273 = [
  4.00   0.49   4.39   18.3   2.14   14.3    52.479   1.45
  4.00   0.65   5.30   21.1   2.76   23.3    66.393   1.45
  4.00   0.34   3.49   16.1   1.53    5.34   38.565   0.79];


  ResetDoses
  Rattus
  Rat_6HCs

% Note: the above M file indicates that Dr. Merrill may have used a PAFAT=0.06 for this study only, but the M
file used 0.7 anyway (DecaneRat)
  BW=0.211; CONC(5)=1200.0; TCHNG=4.0; DOSEINT=24.0; DAYSWK=5.0; TMAX=24.0; CINT=0.1; TSTOP=30.0;
%  PFAT=0.06;
  start @nocallback
  plotcart = plot (0, Perl1200(:,1), Perl1200(:,2), '+b', _t, _cart(:,5), '-b');
  plotcliv = plot (0, Perl1200(:,1), Perl1200(:,3), '+b', _t, _cliv(:,5), '-b');
  plotcbrntot = plot (0, Perl1200(:,1), Perl1200(:,4), '+b', _t, _cbrntot(:,5), '-b');
  plotcrap = plot (0, Perl1200(:,1), Perl1200(:,5), '+b', _t, _crap(:,5), '-b');
  plotcfat = plot (0, Perl1200(:,1), Perl1200(:,6), '+b', _t, _cfat(:,5), '-b');

  CONC(5)=781.0; TSTOP=5.0;
  start @nocallback
  plot (plotcart, 1, Perl781(:,1), Perl781(:,2), '+k', _t, _cart(:,5), '-k');
  plot (plotcliv, 1, Perl781(:,1), Perl781(:,3), '+k', _t, _cliv(:,5), '-k');
  plot (plotcbrntot, 1, Perl781(:,1), Perl781(:,4), '+k', _t, _cbrntot(:,5), '-k');
  plot (plotcrap, 1, Perl781(:,1), Perl781(:,5), '+k', _t, _crap(:,5), '-k');
  plot (plotcfat, 1, Perl781(:,1), Perl781(:,6), '+k', _t, _cfat(:,5), '-k');

  CONC(5)=273.0;
  start @nocallback
  plot (plotcart, 1, Perl273(:,1), Perl273(:,2), '+g', _t, _cart(:,5), '-g');
  plot (plotcliv, 1, Perl273(:,1), Perl273(:,3), '+g', _t, _cliv(:,5), '-g');
  plot (plotcbrntot, 1, Perl273(:,1), Perl273(:,4), '+g', _t, _cbrntot(:,5), '-g');
  plot (plotcrap, 1, Perl273(:,1), Perl273(:,5), '+g', _t, _crap(:,5), '-g');
  plot (plotcfat, 1, Perl273(:,1), Perl273(:,6), '+g', _t, _cfat(:,5), '-g');


set @preference=BackslashEscapes
  pltscript(plotcart, "Chart.Header.Text = \"Perleberg et al. (2004) n-Decane Inhalation Study\";")
  pltscript(plotcart, "Chart.SubHeader.Text = \"273, 781 or 1200 ppm for 4 Hours\";")
  pltscript(plotcart, "Chart.SubHeader.Visible=true;")
  pltscript(plotcart, "Chart.Axes.Left.Automatic=false;")
  pltscript(plotcart, "Chart.Axes.Left.Minimum=0.01;")
  pltscript(plotcart, "Chart.Axes.Left.Maximum=100.0;")
  pltscript(plotcart, "Chart.Axes.Left.Logarithmic = true;")
  pltscript(plotcart, "Chart.Axes.Left.Title.Text=\"Decane in Arterial Blood [mg/L]\";")
  pltscript(plotcart, "Chart.Axes.Bottom.Title.Text=\"Hours\";")
  pltscript(plotcart, "Chart.Series[0].Title=\"1200 ppm\";")
  pltscript(plotcart, "Chart.Series[1].Title=\"Simulation 1200 ppm\";")
  pltscript(plotcart, "Chart.Series[2].Title=\"781 ppm\";")
  pltscript(plotcart, "Chart.Series[3].Title=\"Simulation 781 ppm\";")
  pltscript(plotcart, "Chart.Series[4].Title=\"273 ppm\";")
  pltscript(plotcart, "Chart.Series[5].Title=\"Simulation 273 ppm\";")

  pltscript(plotcliv, "Chart.Header.Text = \"Perleberg et al. (2004) n-Decane Inhalation Study\";")
  pltscript(plotcliv, "Chart.SubHeader.Text = \"273, 781 or 1200 ppm for 4 Hours\";")
  pltscript(plotcliv, "Chart.SubHeader.Visible=true;")
  pltscript(plotcliv, "Chart.Axes.Left.Automatic=false;")
  pltscript(plotcliv, "Chart.Axes.Left.Minimum=0.01;")
  pltscript(plotcliv, "Chart.Axes.Left.Maximum=100.0;")
  pltscript(plotcliv, "Chart.Axes.Left.Logarithmic = true;")
  pltscript(plotcliv, "Chart.Axes.Left.Title.Text=\"Decane in Liver [mg/L]\";")
```

```
  pltscript(plotcliv, "Chart.Axes.Bottom.Title.Text=\"Hours\";")
  pltscript(plotcliv, "Chart.Series[0].Title=\"1200 ppm\";")
  pltscript(plotcliv, "Chart.Series[1].Title=\"Simulation 1200 ppm\";")
  pltscript(plotcliv, "Chart.Series[2].Title=\"781 ppm\";")
  pltscript(plotcliv, "Chart.Series[3].Title=\"Simulation 781 ppm\";")
  pltscript(plotcliv, "Chart.Series[4].Title=\"273 ppm\";")
  pltscript(plotcliv, "Chart.Series[5].Title=\"Simulation 273 ppm\";")

  pltscript(plotcbrntot, "Chart.Header.Text = \"Perleberg et al. (2004) n-Decane Inhalation Study\";")
  pltscript(plotcbrntot, "Chart.SubHeader.Text = \"273, 781 or 1200 ppm for 4 Hours\";")
  pltscript(plotcbrntot, "Chart.SubHeader.Visible=true;")
  pltscript(plotcbrntot, "Chart.Axes.Left.Automatic=false;")
  pltscript(plotcbrntot, "Chart.Axes.Left.Minimum=0.01;")
  pltscript(plotcbrntot, "Chart.Axes.Left.Maximum=200.0;")
  pltscript(plotcbrntot, "Chart.Axes.Left.Logarithmic = true;")
  pltscript(plotcbrntot, "Chart.Axes.Left.Title.Text=\"Decane in Total Brain [mg/L]\";")
  pltscript(plotcbrntot, "Chart.Axes.Bottom.Title.Text=\"Hours\";")
  pltscript(plotcbrntot, "Chart.Series[0].Title=\"1200 ppm\";")
  pltscript(plotcbrntot, "Chart.Series[1].Title=\"Simulation 1200 ppm\";")
  pltscript(plotcbrntot, "Chart.Series[2].Title=\"781 ppm\";")
  pltscript(plotcbrntot, "Chart.Series[3].Title=\"Simulation 781 ppm\";")
  pltscript(plotcbrntot, "Chart.Series[4].Title=\"273 ppm\";")
  pltscript(plotcbrntot, "Chart.Series[5].Title=\"Simulation 273 ppm\";")

  pltscript(plotcrap, "Chart.Header.Text = \"Perleberg et al. (2004) n-Decane Inhalation Study\";")
  pltscript(plotcrap, "Chart.SubHeader.Text = \"273, 781 or 1200 ppm for 4 Hours\";")
  pltscript(plotcrap, "Chart.SubHeader.Visible=true;")
  pltscript(plotcrap, "Chart.Axes.Left.Automatic=false;")
  pltscript(plotcrap, "Chart.Axes.Left.Minimum=0.01;")
  pltscript(plotcrap, "Chart.Axes.Left.Maximum=100.0;")
  pltscript(plotcrap, "Chart.Axes.Left.Logarithmic = true;")
  pltscript(plotcrap, "Chart.Axes.Left.Title.Text=\"Decane in Rapidly Perfused Tissues [mg/L]\";")
  pltscript(plotcrap, "Chart.Axes.Bottom.Title.Text=\"Hours\";")
  pltscript(plotcrap, "Chart.Series[0].Title=\"1200 ppm\";")
  pltscript(plotcrap, "Chart.Series[1].Title=\"Simulation 1200 ppm\";")
  pltscript(plotcrap, "Chart.Series[2].Title=\"781 ppm\";")
  pltscript(plotcrap, "Chart.Series[3].Title=\"Simulation 781 ppm\";")
  pltscript(plotcrap, "Chart.Series[4].Title=\"273 ppm\";")
  pltscript(plotcrap, "Chart.Series[5].Title=\"Simulation 273 ppm\";")

  pltscript(plotcfat, "Chart.Header.Text = \"Perleberg et al. (2004) n-Decane Inhalation Study\";")
  pltscript(plotcfat, "Chart.SubHeader.Text = \"273, 781 or 1200 ppm for 4 Hours\";")
  pltscript(plotcfat, "Chart.SubHeader.Visible=true;")
  pltscript(plotcfat, "Chart.Axes.Left.Logarithmic = true;")
  pltscript(plotcfat, "Chart.Axes.Left.Title.Text=\"Decane in Fat [mg/L]\";")
  pltscript(plotcfat, "Chart.Axes.Bottom.Title.Text=\"Hours\";")
  pltscript(plotcfat, "Chart.Series[0].Title=\"1200 ppm\";")
  pltscript(plotcfat, "Chart.Series[1].Title=\"Simulation 1200 ppm\";")
  pltscript(plotcfat, "Chart.Series[2].Title=\"781 ppm\";")
  pltscript(plotcfat, "Chart.Series[3].Title=\"Simulation 781 ppm\";")
  pltscript(plotcfat, "Chart.Series[4].Title=\"273 ppm\";")
  pltscript(plotcfat, "Chart.Series[5].Title=\"Simulation 273 ppm\";")
set @preference=NoBackslashEscapes
```

C6_NTP00_Rat_RepeatInh_2wk.m

```
% NTP. 2000. NTP Technical Report on the Toxicology and Carcinogenesis Studies of Naphthalene
%  (CAS NO. 91-20-3) in F344/N Rats. National Toxicology Program, Research Triangle Park NC. NTP TR 500.

% Sprague-Dawley rats, n = 3 male or female (if n=1, no SE info)
% 10, 30, or 60 ppm
%    6 hr/d, 5 d/wk, 2 week timepoint
%    Chambers were allowed to empty for 12 min to reduce the concentration enough to remove the rats
%    Therefore TChng = 6.1
%      (adding 6 min of full exposure to average out 12 minutes of exposure decrease from full to 10%)
% BW data from 1 & 4 weeks (Table 4&5) used to calculate rough 2 wk BW of 0.169 kg male & 0.121 kg female

% Time, CVen for 10, 30, 60 ppm after day 12 exposures ended
% Values, top to bottom, are series of average, -SE, +SE in mg/L (females flip to +SE, -SE)


MBlood = [
  270.10   0.331   1.540   3.730
  270.10   0.299   1.473   3.525
```

```
  270.10   0.363   1.607   3.935
  270.60   0.192   0.765   1.640
  270.60   0.177   0.724   1.590
  270.60   0.207   0.806   1.690
  271.10   0.118   NaN     NaN
  271.10   0.111   NaN     NaN
  271.10   0.125   NaN     NaN
  271.60   NaN     0.210   0.544
  271.60   NaN     0.190   0.488
  271.60   NaN     0.230   0.600
  272.10   0.045   NaN     NaN
  275.10   0.015   0.047   NaN
  275.10   0.011   0.043   NaN
  275.10   0.019   0.051   NaN
  276.10   NaN     NaN     0.069
  276.10   NaN     NaN     0.066
  276.10   NaN     NaN     0.072
  278.10   NaN     0.020   NaN
  278.10   NaN     0.016   NaN
  278.10   NaN     0.024   NaN
  282.10   NaN     0.007   0.022
  282.10   NaN     0.007   0.019
  282.10   NaN     0.007   0.025
  286.10   NaN     NaN     0.008
  286.10   NaN     NaN     0.006
  286.10   NaN     NaN     0.010];

FBlood = [
  270.1    0.241 1.137 2.910
  270.1    0.254 1.159 2.950
  270.1    0.228 1.115 2.870
  270.6    0.130 0.606 1.193
  270.6    0.154 0.617 1.320
  270.6    0.106 0.595 1.066
  271.1    0.102 NaN   NaN
  271.1    0.104 NaN   NaN
  271.1    0.100 NaN   NaN
  271.6    NaN   0.200   0.515
  271.6    NaN   0.227   0.525
  271.6    NaN   0.173   0.505
  272.1    0.043 NaN   NaN
  272.1    0.045 NaN   NaN
  272.1    0.041 NaN   NaN
  275.1    0.010 0.049 NaN
  275.1    0.011 0.052 NaN
  275.1    0.009 0.046 NaN
  276.1    NaN   NaN   0.087
  270.1    NaN   NaN   0.104
  270.1    NaN   NaN   0.070
  278.1    0.026 0.016 NaN
  278.1    NaN   0.018   NaN
  278.1    NaN   0.014   NaN
  282.1    NaN   0.008   0.006
  282.1    NaN   NaN   0.007
  282.1    NaN   NaN   0.005
  286.1    NaN   NaN   0.011];

  ResetDoses
  Rattus
  Rat_6HCs

% Study specific male rat
  BW=0.169; CONC(6)=10.0; TCHNG=6.0; DOSEINT=24.0; DAYSWK=5.0; TMAX=270.0; TSTART=0.0; TSTOP=290.0;

% These do not equal the upregulated VMax values in Merrill et al. 2019 but are similar
  VMAXC(6)=VMAXC(6)*1.4;
  VMAXLNGC(6)=VMAXLNGC(6)*1.12;

% Increased VMaxC values due to metabolic upregulation
%  KM = KMLng = 2.18

  start @NoCallback
  plotcvenm = plot (0, MBlood(:,1), MBlood(:,2), '+b', _t, _cven(:,6), '-b');
  plotcvenmlo = plot (0, MBlood(:,1), MBlood(:,2), '+b', _t, _cven(:,6), '-b');

  CONC(6)=30.0;
```

```
  start @NoCallBack
  plot (plotcvenm, 1, MBlood(:,1), MBlood(:,3), '+g', _t, _cven(:,6), '-g');

  CONC(6)=60.0;
  start @NoCallBack
  plot (plotcvenm, 1, MBlood(:,1), MBlood(:,4), '+m', _t, _cven(:,6), '-m');

% Study specific female rat
  BW=0.121; CONC(6)=10.0;
  VMAXC(6)=((8.28/(BW^0.75))*BW)*1.4;
  VMAXLNGC(6)=((0.45/(BW^0.75))*BW)*1.12;

  start @NoCallback
  plotcvenf = plot (0, FBlood(:,1), FBlood(:,2), '+b', _t, _cven(:,6), '-b');
  plotcvenflo = plot (0, FBlood(:,1), FBlood(:,2), '+b', _t, _cven(:,6), '-b');

  CONC(6)=30.0;
  start @NoCallBack
  plot (plotcvenf, 1, FBlood(:,1), FBlood(:,3), '+g', _t, _cven(:,6), '-g');

  CONC(6)=60.0;
  start @NoCallBack
  plot (plotcvenf, 1, FBlood(:,1), FBlood(:,4), '+m', _t, _cven(:,6), '-m');

set @preference=BackslashEscapes
  pltscript(plotcvenm, "Chart.Header.Text = \"NTP (2000) Naphthalene Inhalation Exposure in Male Rats at 2
Wks\";")
  pltscript(plotcvenm, "Chart.Axes.Left.Title.Text=\"Venous Concentration (mg/L)\";")
  pltscript(plotcvenm, "Chart.Axes.Bottom.Title.Text=\"Hours\";")
  pltscript(plotcvenm, "Chart.Series[0].Title=\"10 ppm\";")
  pltscript(plotcvenm, "Chart.Series[1].Title=\"10 ppm Prediction\";")
  pltscript(plotcvenm, "Chart.Series[2].Title=\"30 ppm\";")
  pltscript(plotcvenm, "Chart.Series[3].Title=\"30 ppm Prediction\";")
  pltscript(plotcvenm, "Chart.Series[4].Title=\"60 ppm\";")
  pltscript(plotcvenm, "Chart.Series[5].Title=\"60 ppm Prediction\";")

  pltscript(plotcvenmlo, "Chart.Header.Text = \"NTP (2000) Naphthalene Inhalation Exposure in MaleRats at 2
Wks\";")
  pltscript(plotcvenmlo, "Chart.Axes.Left.Title.Text=\"Venous Concentration (mg/L)\";")
  pltscript(plotcvenmlo, "Chart.Axes.Bottom.Title.Text=\"Hours\";")
  pltscript(plotcvenmlo, "Chart.Series[0].Title=\"10 ppm\";")
  pltscript(plotcvenmlo, "Chart.Series[1].Title=\"10 ppm Prediction\";")

  pltscript(plotcvenf, "Chart.Header.Text = \"NTP (2000) Naphthalene Inhalation Exposure in Male Rats at 2
Wks\";")
  pltscript(plotcvenf, "Chart.Axes.Left.Title.Text=\"Venous Concentration (mg/L)\";")
  pltscript(plotcvenf, "Chart.Axes.Bottom.Title.Text=\"Hours\";")
  pltscript(plotcvenf, "Chart.Series[0].Title=\"10 ppm\";")
  pltscript(plotcvenf, "Chart.Series[1].Title=\"10 ppm Prediction\";")
  pltscript(plotcvenf, "Chart.Series[2].Title=\"30 ppm\";")
  pltscript(plotcvenf, "Chart.Series[3].Title=\"30 ppm Prediction\";")
  pltscript(plotcvenf, "Chart.Series[4].Title=\"60 ppm\";")
  pltscript(plotcvenf, "Chart.Series[5].Title=\"60 ppm Prediction\";")

  pltscript(plotcvenflo, "Chart.Header.Text = \"NTP (2000) Naphthalene Inhalation Exposure in MaleRats at 2
Wks\";")
  pltscript(plotcvenflo, "Chart.Axes.Left.Title.Text=\"Venous Concentration (mg/L)\";")
  pltscript(plotcvenflo, "Chart.Axes.Bottom.Title.Text=\"Hours\";")
  pltscript(plotcvenflo, "Chart.Series[0].Title=\"10 ppm\";")
  pltscript(plotcvenflo, "Chart.Series[1].Title=\"10 ppm Prediction\";")
set @preference=NoBackslashEscapes
```

C6_NTP00_MFRat_SingleInh.m

```
% NTP. 2000. NTP Technical Report on the Toxicology and Carcinogenesis Studies of Naphthalene
%  (CAS NO. 91-20-3) in F344/N Rats. National Toxicology Program, Research Triangle Park NC. NTP TR 500.

% Sprague-Dawley rats, n = 3 males or females
% 10, 30, or 60 ppm
%    6 hr single exposure
%    Chambers were allowed to empty for 12 min to reduce the concentration enough to remove the rats
%    Therefore TChng = 6.1
%      (adding 6 min of full exposure to average out 12 minutes of exposure decrease from full to 10%)
% BW data from Table D5: Male = 125 g, Female = 100 g
```

```
% Time, CVen for 10, 30, 60 ppm single dose after 6.1 hours of exposure
% Values, top to bottom, are series of average, -SE, +SE in mg/L
MBlood = [
   6.1   0.463   1.387   5.360
   6.1   0.497   1.439   6.026
   6.1   0.429   1.335   4.694
   6.6   0.308   0.911   3.193
   6.6   0.317   0.962   3.529
   6.6   0.299   0.860   2.857
   7.1   0.171   0.661   2.227
   7.1   0.179   0.720   2.615
   7.1   0.163   0.602   1.839
   7.6   0.094   0.476   1.143
   7.6   0.103   0.494   1.399
   7.6   0.085   0.458   0.887
   8.1   0.100   0.239   0.838
   8.1   0.111   0.267   0.987
   8.1   0.089   0.211   0.689
  10.1   0.051   0.138   0.380
  10.1   0.053   0.145   0.422
  10.1   0.049   0.131   0.338
  12.1   0.029   0.071   0.252
  12.1   0.030   0.072   0.261
  12.1   0.028   0.070   0.243
  14.1   0.014   0.060   0.174
  14.1   0.017   0.063   0.197
  14.1   0.011   0.057   0.151];

FBlood = [
  6.1 0.442 1.667 4.850
  6.1 0.471 1.824 4.962
  6.1 0.413 1.510 4.738
  6.6 0.243 0.841 2.483
  6.6 0.250 0.927 2.625
  6.6 0.236 0.755 2.341
  7.1 0.135 0.632 1.610
  7.1 0.143 0.697 1.896
  7.1 0.127 0.567 1.324
  7.6 0.087 0.397 0.870
  7.6 0.101 0.443 1.028
  7.6 0.073 0.351 0.712
  8.1 0.101 0.408 0.868
  8.1 0.115 0.439 0.940
  8.1 0.087 0.377 0.796
 10.1 0.050 0.182 0.428
 10.1 0.057 0.198 0.506
 10.1 0.043 0.166 0.350
 12.1 0.034 0.100 0.312
 12.1 0.039 0.119 0.332
 12.1 0.029 0.081 0.292
 14.1 0.016 0.069 0.192
 14.1 0.022 0.078 0.219
 14.1 0.010 0.060 0.165];

  ResetDoses
  Rattus
  Rat_6HCs

% Study specific male rat
  BW=0.125; CONC(6)=10.0; TCHNG=6.0; DOSEINT=24.0; DAYSWK=5.0; TMAX=24.0; TSTART=0.0; TSTOP=24.0;
  start @NoCallback
  plotcvenm = plot (0, MBlood(:,1), MBlood(:,2), '+b', _t, _cven(:,6), '-b');
  plotcvenmlo = plot (0, MBlood(:,1), MBlood(:,2), '+b', _t, _cven(:,6), '-b');

  CONC(6)=30.0;
  start @NoCallBack
  plot (plotcvenm, 1, MBlood(:,1), MBlood(:,3), '+g', _t, _cven(:,6), '-g');

  CONC(6)=60.0;
  start @NoCallBack
  plot (plotcvenm, 1, MBlood(:,1), MBlood(:,4), '+m', _t, _cven(:,6), '-m');

% Study specific female rat
  BW=0.1; CONC(6)=10.0;
```

```
  VMAXC(6)=((8.28/(BW^0.75))*BW);
  VMAXLNGC(6)=((0.45/(BW^0.75))*BW);
  start @NoCallback
  plotcvenf = plot (0, FBlood(:,1), FBlood(:,2), '+b', _t, _cven(:,6), '-b');
  plotcvenflo = plot (0, FBlood(:,1), FBlood(:,2), '+b', _t, _cven(:,6), '-b');

  CONC(6)=30.0;
  start @NoCallBack
  plot (plotcvenf, 1, FBlood(:,1), FBlood(:,3), '+g', _t, _cven(:,6), '-g');

  CONC(6)=60.0;
  start @NoCallBack
  plot (plotcvenf, 1, FBlood(:,1), FBlood(:,4), '+m', _t, _cven(:,6), '-m');

set @preference=BackslashEscapes
  pltscript(plotcvenm, "Chart.Header.Text = \"NTP (2000) Naphthalene Single Inhalation Exposure in Male
Rats\";")
  pltscript(plotcvenm, "Chart.Axes.Left.Title.Text=\"Venous Concentration (mg/L)\";")
  pltscript(plotcvenm, "Chart.Axes.Bottom.Title.Text=\"Hours\";")
  pltscript(plotcvenm, "Chart.Series[0].Title=\"10 ppm\";")
  pltscript(plotcvenm, "Chart.Series[1].Title=\"10 ppm Prediction\";")
  pltscript(plotcvenm, "Chart.Series[2].Title=\"30 ppm\";")
  pltscript(plotcvenm, "Chart.Series[3].Title=\"30 ppm Prediction\";")
  pltscript(plotcvenm, "Chart.Series[4].Title=\"60 ppm\";")
  pltscript(plotcvenm, "Chart.Series[5].Title=\"60 ppm Prediction\";")

  pltscript(plotcvenmlo, "Chart.Header.Text = \"NTP (2000) Naphthalene Single Inhalation Exposure in Male
Rats\";")
  pltscript(plotcvenmlo, "Chart.Axes.Left.Title.Text=\"Venous Concentration (mg/L)\";")
  pltscript(plotcvenmlo, "Chart.Axes.Bottom.Title.Text=\"Hours\";")
  pltscript(plotcvenmlo, "Chart.Series[0].Title=\"10 ppm\";")
  pltscript(plotcvenmlo, "Chart.Series[1].Title=\"10 ppm Prediction\";")

    pltscript(plotcvenf, "Chart.Header.Text = \"NTP (2000) Naphthalene Single Inhalation Exposure in Female
Rats\";")
  pltscript(plotcvenf, "Chart.Axes.Left.Title.Text=\"Venous Concentration (mg/L)\";")
  pltscript(plotcvenf, "Chart.Axes.Bottom.Title.Text=\"Hours\";")
  pltscript(plotcvenf, "Chart.Series[0].Title=\"10 ppm\";")
  pltscript(plotcvenf, "Chart.Series[1].Title=\"10 ppm Prediction\";")
  pltscript(plotcvenf, "Chart.Series[2].Title=\"30 ppm\";")
  pltscript(plotcvenf, "Chart.Series[3].Title=\"30 ppm Prediction\";")
  pltscript(plotcvenf, "Chart.Series[4].Title=\"60 ppm\";")
  pltscript(plotcvenf, "Chart.Series[5].Title=\"60 ppm Prediction\";")

  pltscript(plotcvenflo, "Chart.Header.Text = \"NTP (2000) Naphthalene Single Inhalation Exposure in Female
Rats\";")
  pltscript(plotcvenflo, "Chart.Axes.Left.Title.Text=\"Venous Concentration (mg/L)\";")
  pltscript(plotcvenflo, "Chart.Axes.Bottom.Title.Text=\"Hours\";")
  pltscript(plotcvenflo, "Chart.Series[0].Title=\"10 ppm\";")
  pltscript(plotcvenflo, "Chart.Series[1].Title=\"10 ppm Prediction\";")
set @preference=NoBackslashEscapes
```

C6_RTI96_Rat_IV.m

```
% Simulates data from RTI 1996
%   SE and individual data are available
% Data used in publications by Quick and Shuler (1999),
%   Willems et al. (2001), Campbell et al. (2014).


% male rats, 1, 3 and 10 mg/kg, time and mean cvenbldna
Male=[
  0.03   1.980   3.580    9.330
  0.03   3.080   8.280   11.030
  0.03   0.880   0.000    7.630
  0.08   0.668   2.310    5.050
  0.08   0.707   4.610    6.150
  0.08   0.629   0.010    3.950
  0.17   0.322   2.120    2.700
  0.17   0.337   3.920    2.830
  0.17   0.307   0.320    2.570
  0.33   0.106   0.720    0.787
  0.33   0.128   1.250    0.897
  0.33   0.084   0.190    0.677
```

```
  0.67   0.039   0.245    0.447
  0.67   0.059   0.312    0.487
  0.67   0.019   0.178    0.407
  1.00   0.338   0.158    0.186
  1.00   0.341   0.180    0.210
  1.00   0.335   0.136    0.162
  2.00   0.017   0.123    0.086
  2.00   NaN     0.220    0.093
  2.00   NaN     0.026    0.078
  4.00   0.014   0.053    0.048
  4.00   NaN     0.101    0.058
  4.00   NaN     0.005    0.038
  6.00   0.011   0.091    0.036
  6.00   NaN     NaN      0.039
  6.00   NaN     NaN      0.032
  8.00   0.007   0.021    0.014
  8.00   NaN     NaN      0.018
  8.00   NaN     NaN      0.010];


% female rats, 1, 3 or 10 mg/kg, time and mean cvenbldna
Fem=[
  0.03   1.030   4.140   10.200
  0.03   1.930   6.440   12.300
  0.03   0.130   1.840    8.100
  0.08   0.457   2.100    6.130
  0.08   0.827   2.850    9.530
  0.08   0.087   1.350    2.730
  0.17   0.209   1.780    2.220
  0.17   0.409   2.880    2.640
  0.17   0.009   0.680    1.800
  0.33   0.057   0.457    0.860
  0.33   0.112   0.535    1.090
  0.33   0.002   0.379    0.630
  0.67   0.029   0.179    0.333
  0.67   0.046   0.251    0.414
  0.67   0.012   0.107    0.252
  1.00   0.018   0.108    0.211
  1.00   0.022   0.139    0.234
  1.00   0.013   0.077    0.188
  2.00   0.013   0.047    0.092
  2.00   0.020   0.058    0.104
  2.00   0.005   0.036    0.080
  4.00   0.014   0.022    0.061
  4.00   0.021   0.032    0.064
  4.00   0.007   0.012    0.058
  6.00   0.016   0.026    0.050
  6.00   0.027   0.028    0.056
  6.00   0.005   0.024    0.045
  8.00   0.011   0.024    0.029
  8.00   NaN     NaN      0.031
  8.00   NaN     NaN      0.027];


  ResetDoses
  Rattus
  Rat_6HCs

% MALE SIMULATIONS
% 1 mg/kg
  BW=0.255; IVDOSE(6)=1.0; TINF=0.008, TSTOP=8.0;
  start @nocallback
  plotcven = plot (0, Male(:,1), Male(:,2), '+b', _t, _cven(:,6), '-b');
  plotcvenlo = plot (0, Male(:,1), Male(:,2), '+b', _t, _cven(:,6), '-b');

% 3 mg/kg
  IVDOSE(6)=3.0;
  start @nocallback
  plot (plotcven, 1,  Male(:,1), Male(:,3), '+g', _t, _cven(:,6), '-g');

% 10 mg/kg
  IVDOSE(6)=10.0;
  start @nocallback
  plot (plotcven, 1,  Male(:,1), Male(:,4), '+m', _t, _cven(:,6), '-m');
```

```
% FEMALE SIMULATIONS
% 1 mg/kg
  BW=0.156; IVDOSE(6)=1.0; TINF=0.008, TSTOP=8.0;
  VMAXC(6)=((8.28/(BW^0.75))*BW);
  VMAXLNGC(6)=((0.45/(BW^0.75))*BW);
  start @nocallback
  plotcvenf = plot (0, Fem(:,1), Fem(:,2), '+b', _t, _cven(:,6), '-b');
  plotcvenflo = plot (0, Fem(:,1), Fem(:,2), '+b', _t, _cven(:,6), '-b');

% 3 mg/kg
  IVDOSE(6)=3.0;
  start @nocallback
  plot (plotcvenf, 1,  Fem(:,1), Fem(:,3), '+g', _t, _cven(:,6), '-g');

% 10 mg/kg
  IVDOSE(6)=10.0;
  start @nocallback
  plot (plotcvenf, 1,  Fem(:,1), Fem(:,4), '+m', _t, _cven(:,6), '-m');


set @preference=BackslashEscapes
  pltscript(plotcven, "Chart.Header.Text = \"RTI 1996 IV Study delivered to NTP - Male Rats\";")
  pltscript(plotcven, "Chart.Axes.Left.LogarithmicBase=10;")
  pltscript(plotcven, "Chart.Axes.Left.Logarithmic=true;")
  pltscript(plotcven, "Chart.Axes.Left.Automatic=false;")
  pltscript(plotcven, "Chart.Axes.Left.Minimum=0.001;")
  pltscript(plotcven, "Chart.Axes.Left.Maximum=100.0;")
  pltscript(plotcven, "Chart.Axes.Left.Title.Text=\"Venous Concentration (mg/L)\";")
  pltscript(plotcven, "Chart.Axes.Bottom.Title.Text=\"Hours\";")
  pltscript(plotcven, "Chart.Series[0].Title=\"1 mg/kg Simulation\";")
  pltscript(plotcven, "Chart.Series[1].Title=\"1 mg/kg Data\";")
  pltscript(plotcven, "Chart.Series[2].Title=\"3 mg/kg Simulation\";")
  pltscript(plotcven, "Chart.Series[3].Title=\"3 mg/kg Data\";")
  pltscript(plotcven, "Chart.Series[4].Title=\"10 mg/kg Simulation\";")
  pltscript(plotcven, "Chart.Series[5].Title=\"10 mg/kg Data\";")

  pltscript(plotcvenlo, "Chart.Header.Text = \"RTI 1996 IV Study delivered to NTP - Male Rats\";")
  pltscript(plotcvenlo, "Chart.Axes.Left.LogarithmicBase=10;")
  pltscript(plotcvenlo, "Chart.Axes.Left.Logarithmic=true;")
  pltscript(plotcvenlo, "Chart.Axes.Left.Automatic=false;")
  pltscript(plotcvenlo, "Chart.Axes.Left.Minimum=0.001;")
  pltscript(plotcvenlo, "Chart.Axes.Left.Maximum=100.0;")
  pltscript(plotcvenlo, "Chart.Axes.Left.Title.Text=\"Venous Concentration (mg/L)\";")
  pltscript(plotcvenlo, "Chart.Axes.Bottom.Title.Text=\"Hours\";")
  pltscript(plotcvenlo, "Chart.Series[0].Title=\"1 mg/kg Simulation\";")
  pltscript(plotcvenlo, "Chart.Series[1].Title=\"1 mg/kg Data\";")

  pltscript(plotcvenf, "Chart.Header.Text = \"RTI 1996 IV Study delivered to NTP - Female Rats\";")
  pltscript(plotcvenf, "Chart.Axes.Left.LogarithmicBase=10;")
  pltscript(plotcvenf, "Chart.Axes.Left.Logarithmic=true;")
  pltscript(plotcvenf, "Chart.Axes.Left.Automatic=false;")
  pltscript(plotcvenf, "Chart.Axes.Left.Minimum=0.001;")
  pltscript(plotcvenf, "Chart.Axes.Left.Maximum=100.0;")
  pltscript(plotcvenf, "Chart.Axes.Left.Title.Text=\"Venous Concentration (mg/L)\";")
  pltscript(plotcvenf, "Chart.Axes.Bottom.Title.Text=\"Hours\";")
  pltscript(plotcvenf, "Chart.Series[0].Title=\"1 mg/kg Simulation\";")
  pltscript(plotcvenf, "Chart.Series[1].Title=\"1 mg/kg Data\";")
  pltscript(plotcvenf, "Chart.Series[2].Title=\"3 mg/kg Simulation\";")
  pltscript(plotcvenf, "Chart.Series[3].Title=\"3 mg/kg Data\";")
  pltscript(plotcvenf, "Chart.Series[4].Title=\"10 mg/kg Simulation\";")
  pltscript(plotcvenf, "Chart.Series[5].Title=\"10 mg/kg Data\";")

  pltscript(plotcvenflo, "Chart.Header.Text = \"RTI 1996 IV Study delivered to NTP - Female Rats\";")
  pltscript(plotcvenflo, "Chart.Axes.Left.LogarithmicBase=10;")
  pltscript(plotcvenflo, "Chart.Axes.Left.Logarithmic=true;")
  pltscript(plotcvenflo, "Chart.Axes.Left.Automatic=false;")
  pltscript(plotcvenflo, "Chart.Axes.Left.Minimum=0.001;")
  pltscript(plotcvenflo, "Chart.Axes.Left.Maximum=100.0;")
  pltscript(plotcvenflo, "Chart.Axes.Left.Title.Text=\"Venous Concentration (mg/L)\";")
  pltscript(plotcvenflo, "Chart.Axes.Bottom.Title.Text=\"Hours\";")
  pltscript(plotcvenflo, "Chart.Series[0].Title=\"1 mg/kg Simulation\";")
  pltscript(plotcvenflo, "Chart.Series[1].Title=\"1 mg/kg Data\";")
set @preference=NoBackslashEscapes
```

5. Guthrie *et al.* Prediction .m File

This .m file was used to produce Figure 5 in the main article.

Predict_Guthrie14_scaled.m

```
% Simulates 6 key hydrocarbons for Guthrie et al. 2014 exposure to predict rat target tissue concentrations

% Guthrie, O.W., Xu, H., Wong, B.A., McInturf, S.M., Reboulet, J.E., Ortiz, P.A. and Mattie, D.R. 2014.
%     Exposure to low levels of jet-propulsion fuel impairs brainstem encoding of stimulus intensity.
%     J Toxicol Environ Health A 77(5): 261-280.

  ResetDoses
  Rattus
  Rat_6HCs

% Calculates concentration of each component in POSF 4658, converted from 1000 mg/m3 to ppm, assuming STP
% Weight percent values from tandem gas chromatography analysis by the 2006 Shafer et al. method
% POSF 4658 contains 0.16 percent toluene, 0.11 percent ethylbenzene,
%     0.67 percent xylenes, 1.14 percent nonane, 2.55 percent decane, 0.12% naphthalene
%     File name: JPC5 Fuels Comparison-Reanalysis Version.xlsx
% Average starting BW in Guthrie et al. 2014
% Simulates 1 day of 4 week study
  BW=0.105; TCHNG=6.0; TSTOP=12.0;
  CONC(1)=((0.0016*1000)*24.45)/MW(1);
  CONC(2)=((0.0011*1000)*24.45)/MW(2);
  CONC(3)=((0.0067*1000)*24.45)/MW(3);
  CONC(4)=((0.0114*1000)*24.45)/MW(4);
  CONC(5)=((0.0255*1000)*24.45)/MW(5);
  CONC(6)=((0.0012*1000)*24.45)/MW(6);
  start @nocallback
  plotcbrnstm = plot (0, _t, _cbrnstm(:,1), '-b', _t, _cbrnstm(:,2),'-k', _t, _cbrnstm(:,3),'-r', _t,
_cbrnstm(:,4),'-g', _t, _cbrnstm(:,5),'-m', _t, _cbrnstm(:,6),'-c');
  plotcbrntl = plot (0, _t, _cbrntl(:,1), '-b', _t, _cbrntl(:,2),'-k', _t, _cbrntl(:,3),'-r', _t,
_cbrntl(:,4),'-g', _t, _cbrntl(:,5),'-m', _t, _cbrntl(:,6),'-c');
  plotcbrn = plot (0, _t, _cbrn(:,1), '-b', _t, _cbrn(:,2),'-k', _t, _cbrn(:,3),'-r', _t, _cbrn(:,4),'-g',
_t, _cbrn(:,5),'-m', _t, _cbrn(:,6),'-c');
  plotccoc = plot (0, _t, _ccoc(:,1), '-b', _t, _ccoc(:,2),'-k', _t, _ccoc(:,3),'-r', _t, _ccoc(:,4),'-g',
_t, _ccoc(:,5),'-m', _t, _ccoc(:,6));
  plotcliv = plot (0, _t, _cliv(:,1), '-b', _t, _cliv(:,2),'-k', _t, _cliv(:,3),'-r', _t, _cliv(:,4),'-g',
_t, _cliv(:,5),'-m', _t, _cliv(:,6),'-c');
  plotclng = plot (0, _t, _clng(:,1), '-b', _t, _clng(:,2),'-k', _t, _clng(:,3),'-r', _t, _clng(:,4),'-g',
_t, _clng(:,5),'-m', _t, _clng(:,6),'-c');
  plotcven = plot (0, _t, _cven(:,1), '-b', _t, _cven(:,2),'-k', _t, _cven(:,3),'-r', _t, _cven(:,4),'-g',
_t, _cven(:,5),'-m', _t, _cven(:,6),'-c');


set @preference=BackslashEscapes
  pltscript(plotcbrnstm, "Chart.Header.Text = \"Predictions for Guthrie et al. (2014) Exposure\";")
  pltscript(plotcbrnstm, "Chart.SubHeader.Text = \"6 Key Hydrocarbons in JP-8\";")
  pltscript(plotcbrnstm, "Chart.SubHeader.Visible=true;")
  pltscript(plotcbrnstm, "Chart.Axes.Left.Automatic=false;")
  pltscript(plotcbrnstm, "Chart.Axes.Left.Minimum=0.0;")
  pltscript(plotcbrnstm, "Chart.Axes.Left.Maximum=0.4;")
  pltscript(plotcbrnstm, "Chart.Axes.Left.Title.Text=\"Brain Stem [mg/L]\";")
  pltscript(plotcbrnstm, "Chart.Axes.Bottom.Title.Text=\"Hours\";")
  pltscript(plotcbrnstm, "Chart.Series[0].Title=\"Toluene\";")
  pltscript(plotcbrnstm, "Chart.Series[1].Title=\"Ethylbenzene\";")
  pltscript(plotcbrnstm, "Chart.Series[2].Title=\"Xylene\";")
  pltscript(plotcbrnstm, "Chart.Series[3].Title=\"Nonane\";")
  pltscript(plotcbrnstm, "Chart.Series[4].Title=\"Decane\";")
  pltscript(plotcbrnstm, "Chart.Series[5].Title=\"Naphthalene\";")

  pltscript(plotcbrntl, "Chart.Header.Text = \"Predictions for Guthrie et al. (2014) Exposure\";")
  pltscript(plotcbrntl, "Chart.SubHeader.Text = \"6 Key Hydrocarbons in JP-8\";")
  pltscript(plotcbrntl, "Chart.SubHeader.Visible=true;")
  pltscript(plotcbrntl, "Chart.Axes.Left.Automatic=false;")
  pltscript(plotcbrntl, "Chart.Axes.Left.Minimum=0.0;")
  pltscript(plotcbrntl, "Chart.Axes.Left.Maximum=0.4;")
  pltscript(plotcbrntl, "Chart.Axes.Left.Title.Text=\"Temporal Lobe [mg/L]\";")
  pltscript(plotcbrntl, "Chart.Axes.Bottom.Title.Text=\"Hours\";")
  pltscript(plotcbrntl, "Chart.Series[0].Title=\"Toluene\";")
  pltscript(plotcbrntl, "Chart.Series[1].Title=\"Ethylbenzene\";")
  pltscript(plotcbrntl, "Chart.Series[2].Title=\"Xylene\";")
  pltscript(plotcbrntl, "Chart.Series[3].Title=\"Nonane\";")
```

```
pltscript(plotcbrntl, "Chart.Series[4].Title=\"Decane\";")
pltscript(plotcbrntl, "Chart.Series[5].Title=\"Naphthalene\";")

pltscript(plotcbrn, "Chart.Header.Text = \"Predictions for Guthrie et al. (2014) Exposure\";")
pltscript(plotcbrn, "Chart.SubHeader.Text = \"6 Key Hydrocarbons in JP-8\";")
pltscript(plotcbrn, "Chart.SubHeader.Visible=true;")
pltscript(plotcbrn, "Chart.Axes.Left.Automatic=false;")
pltscript(plotcbrn, "Chart.Axes.Left.Minimum=0.0;")
pltscript(plotcbrn, "Chart.Axes.Left.Maximum=0.4;")
pltscript(plotcbrn, "Chart.Axes.Left.Title.Text=\"Remainder of the Brain [mg/L]\";")
pltscript(plotcbrn, "Chart.Axes.Bottom.Title.Text=\"Hours\";")
pltscript(plotcbrn, "Chart.Series[0].Title=\"Toluene\";")
pltscript(plotcbrn, "Chart.Series[1].Title=\"Ethylbenzene\";")
pltscript(plotcbrn, "Chart.Series[2].Title=\"Xylene\";")
pltscript(plotcbrn, "Chart.Series[3].Title=\"Nonane\";")
pltscript(plotcbrn, "Chart.Series[4].Title=\"Decane\";")
pltscript(plotcbrn, "Chart.Series[5].Title=\"Naphthalene\";")

pltscript(plotccoc, "Chart.Header.Text = \"Predictions for Guthrie et al. (2014) Exposure\";")
pltscript(plotccoc, "Chart.SubHeader.Text = \"6 Key Hydrocarbons in JP-8\";")
pltscript(plotccoc, "Chart.SubHeader.Visible=true;")
pltscript(plotccoc, "Chart.Axes.Left.Automatic=false;")
pltscript(plotccoc, "Chart.Axes.Left.Minimum=0.0;")
pltscript(plotccoc, "Chart.Axes.Left.Maximum=0.4;")
pltscript(plotccoc, "Chart.Axes.Left.Title.Text=\"Cochlea [mg/L]\";")
pltscript(plotccoc, "Chart.Axes.Bottom.Title.Text=\"Hours\";")
pltscript(plotccoc, "Chart.Series[0].Title=\"Toluene\";")
pltscript(plotccoc, "Chart.Series[1].Title=\"Ethylbenzene\";")
pltscript(plotccoc, "Chart.Series[2].Title=\"Xylene\";")
pltscript(plotccoc, "Chart.Series[3].Title=\"Nonane\";")
pltscript(plotccoc, "Chart.Series[4].Title=\"Decane\";")
pltscript(plotccoc, "Chart.Series[5].Title=\"Naphthalene\";")

pltscript(plotcliv, "Chart.Header.Text = \"Predictions for Guthrie et al. (2014) Exposure\";")
pltscript(plotcliv, "Chart.SubHeader.Text = \"6 Key Hydrocarbons in JP-8\";")
pltscript(plotcliv, "Chart.SubHeader.Visible=true;")
pltscript(plotcliv, "Chart.Axes.Left.Automatic=false;")
pltscript(plotcliv, "Chart.Axes.Left.Minimum=0.0;")
pltscript(plotcliv, "Chart.Axes.Left.Maximum=0.4;")
pltscript(plotcliv, "Chart.Axes.Left.Title.Text=\"Liver [mg/L]\";")
pltscript(plotcliv, "Chart.Axes.Bottom.Title.Text=\"Hours\";")
pltscript(plotcliv, "Chart.Series[0].Title=\"Toluene\";")
pltscript(plotcliv, "Chart.Series[1].Title=\"Ethylbenzene\";")
pltscript(plotcliv, "Chart.Series[2].Title=\"Xylene\";")
pltscript(plotcliv, "Chart.Series[3].Title=\"Nonane\";")
pltscript(plotcliv, "Chart.Series[4].Title=\"Decane\";")
pltscript(plotcliv, "Chart.Series[5].Title=\"Naphthalene\";")

pltscript(plotclng, "Chart.Header.Text = \"Predictions for Guthrie et al. (2014) Exposure\";")
pltscript(plotclng, "Chart.SubHeader.Text = \"5 Key Hydrocarbons in JP-8\";")
pltscript(plotclng, "Chart.SubHeader.Visible=true;")
pltscript(plotclng, "Chart.Axes.Left.Automatic=false;")
pltscript(plotclng, "Chart.Axes.Left.Minimum=0.0;")
pltscript(plotclng, "Chart.Axes.Left.Maximum=0.4;")
pltscript(plotclng, "Chart.Axes.Left.Title.Text=\"Lung [mg/L]\";")
pltscript(plotclng, "Chart.Axes.Bottom.Title.Text=\"Hours\";")
pltscript(plotclng, "Chart.Series[0].Title=\"Toluene\";")
pltscript(plotclng, "Chart.Series[1].Title=\"Ethylbenzene\";")
pltscript(plotclng, "Chart.Series[2].Title=\"Xylene\";")
pltscript(plotclng, "Chart.Series[3].Title=\"Nonane\";")
pltscript(plotclng, "Chart.Series[4].Title=\"Decane\";")
pltscript(plotclng, "Chart.Series[5].Title=\"Naphthalene\";")

pltscript(plotcven, "Chart.Header.Text = \"Predictions for Guthrie et al. (2014) Exposure\";")
pltscript(plotcven, "Chart.SubHeader.Text = \"5 Key Hydrocarbons in JP-8\";")
pltscript(plotcven, "Chart.SubHeader.Visible=true;")
pltscript(plotcven, "Chart.Axes.Left.Automatic=false;")
pltscript(plotcven, "Chart.Axes.Left.Minimum=0.0;")
pltscript(plotcven, "Chart.Axes.Left.Maximum=0.4;")
pltscript(plotcven, "Chart.Axes.Left.Title.Text=\"Venous Blood [mg/L]\";")
pltscript(plotcven, "Chart.Axes.Bottom.Title.Text=\"Hours\";")
pltscript(plotcven, "Chart.Series[0].Title=\"Toluene\";")
pltscript(plotcven, "Chart.Series[1].Title=\"Ethylbenzene\";")
pltscript(plotcven, "Chart.Series[2].Title=\"Xylene\";")
pltscript(plotcven, "Chart.Series[3].Title=\"Nonane\";")
pltscript(plotcven, "Chart.Series[4].Title=\"Decane\";")
```

```
  pltscript(plotcven, "Chart.Series[5].Title=\"Naphthalene\";")
set @preference=NoBackslashEscapes
```

6. Fuels Comparison Prediction .m File

These .m files were used to produce Figures 6-7 in the main article.

Predict_RatOEL_scaled.m

```
% Simulates 6 key hydrocarbons at OEL concentration for jet fuels

  ResetDoses
  Rattus
  Rat_6HCs


% Calculates concentration of each component in POSF 4658, converted from 200 mg/m3 to ppm, assuming STP
% Weight percent values from tandem gas chromatography analysis by the 2006 Shafer et al. method
% POSF 4658 contains 0.16 percent toluene, 0.11 percent ethylbenzene,
%     0.67 percent xylenes, 1.14 percent nonane, 2.55 percent decane, 0.12% naphthalene
%     File name: JPC5 Fuels Comparison-Reanalysis Version.xlsx
% Simulates 8 hour work day
  BW=0.25; TCHNG=8.0; TSTOP=12.0;
  CONC(1)=((0.0016*200.0)*24.45)/MW(1);
  CONC(2)=((0.0011*200.0)*24.45)/MW(2);
  CONC(3)=((0.0067*200.0)*24.45)/MW(3);
  CONC(4)=((0.0114*200.0)*24.45)/MW(4);
  CONC(5)=((0.0255*200.0)*24.45)/MW(5);
  CONC(6)=((0.0012*200.0)*24.45)/MW(6);
  start @nocallback
  plotcbrnstm = plot (0, _t, _cbrnstm(:,1), '-b', _t, _cbrnstm(:,2),'-k', _t, _cbrnstm(:,3),'-r', _t,
_cbrnstm(:,4),'-g', _t, _cbrnstm(:,5),'-m', _t, _cbrnstm(:,6),'-c');
  plotcbrntl = plot (0, _t, _cbrntl(:,1), '-b', _t, _cbrntl(:,2),'-k', _t, _cbrntl(:,3),'-r', _t,
_cbrntl(:,4),'-g', _t, _cbrntl(:,5),'-m', _t, _cbrntl(:,6),'-c');
  plotcbrn = plot (0, _t, _cbrn(:,1), '-b', _t, _cbrn(:,2),'-k', _t, _cbrn(:,3),'-r', _t, _cbrn(:,4),'-g', _t,
_cbrn(:,5),'-m', _t, _cbrn(:,6),'-c');
  plotccoc = plot (0, _t, _ccoc(:,1), '-b', _t, _ccoc(:,2),'-k', _t, _ccoc(:,3),'-r', _t, _ccoc(:,4),'-g', _t,
_ccoc(:,5),'-m', _t, _ccoc(:,6),'-c');
  plotcliv = plot (0, _t, _cliv(:,1), '-b', _t, _cliv(:,2),'-k', _t, _cliv(:,3),'-r', _t, _cliv(:,4),'-g', _t,
_cliv(:,5),'-m', _t, _cliv(:,6),'-c');
  plotclng = plot (0, _t, _clng(:,1), '-b', _t, _clng(:,2),'-k', _t, _clng(:,3),'-r', _t, _clng(:,4),'-g', _t,
_clng(:,5),'-m', _t, _clng(:,6),'-c');
  plotcven = plot (0, _t, _cven(:,1), '-b', _t, _cven(:,2),'-k', _t, _cven(:,3),'-r', _t, _cven(:,4),'-g', _t,
_cven(:,5),'-m', _t, _cven(:,6),'-c');

set @preference=BackslashEscapes
    pltscript(plotcbrnstm, "Chart.Header.Text = \"Predictions for OEL Exposure - scaled\";")
    pltscript(plotcbrnstm, "Chart.SubHeader.Text = \"6 Key Hydrocarbons in JP-8\";")
    pltscript(plotcbrnstm, "Chart.SubHeader.Visible=true;")
    pltscript(plotcbrnstm, "Chart.Axes.Left.Automatic=false;")
    pltscript(plotcbrnstm, "Chart.Axes.Left.Minimum=0.0;")
    pltscript(plotcbrnstm, "Chart.Axes.Left.Maximum=0.07;")
    pltscript(plotcbrnstm, "Chart.Axes.Left.Title.Text=\"Brain Stem [mg/L]\";")
    pltscript(plotcbrnstm, "Chart.Axes.Bottom.Title.Text=\"Hours\";")
    pltscript(plotcbrnstm, "Chart.Series[0].Title=\"Toluene\";")
    pltscript(plotcbrnstm, "Chart.Series[1].Title=\"Ethylbenzene\";")
    pltscript(plotcbrnstm, "Chart.Series[2].Title=\"Xylene\";")
    pltscript(plotcbrnstm, "Chart.Series[3].Title=\"Nonane\";")
    pltscript(plotcbrnstm, "Chart.Series[4].Title=\"Decane\";")
    pltscript(plotcbrnstm, "Chart.Series[5].Title=\"Naphthalene\";")

    pltscript(plotcbrntl, "Chart.Header.Text = \"Predictions for OEL Exposure - scaled\";")
    pltscript(plotcbrntl, "Chart.SubHeader.Text = \"6 Key Hydrocarbons in JP-8\";")
    pltscript(plotcbrntl, "Chart.SubHeader.Visible=true;")
    pltscript(plotcbrntl, "Chart.Axes.Left.Automatic=false;")
    pltscript(plotcbrntl, "Chart.Axes.Left.Minimum=0.0;")
    pltscript(plotcbrntl, "Chart.Axes.Left.Maximum=0.07;")
    pltscript(plotcbrntl, "Chart.Axes.Left.Title.Text=\"Temporal Lobe [mg/L]\";")
    pltscript(plotcbrntl, "Chart.Axes.Bottom.Title.Text=\"Hours\";")
    pltscript(plotcbrntl, "Chart.Series[0].Title=\"Toluene\";")
    pltscript(plotcbrntl, "Chart.Series[1].Title=\"Ethylbenzene\";")
    pltscript(plotcbrntl, "Chart.Series[2].Title=\"Xylene\";")
    pltscript(plotcbrntl, "Chart.Series[3].Title=\"Nonane\";")
```

```
pltscript(plotcbrntl, "Chart.Series[4].Title=\"Decane\";")
pltscript(plotcbrntl, "Chart.Series[5].Title=\"Naphthalene\";")

pltscript(plotcbrn, "Chart.Header.Text = \"Predictions for OEL Exposure - scaled\";")
pltscript(plotcbrn, "Chart.SubHeader.Text = \"6 Key Hydrocarbons in JP-8\";")
pltscript(plotcbrn, "Chart.SubHeader.Visible=true;")
pltscript(plotcbrn, "Chart.Axes.Left.Automatic=false;")
pltscript(plotcbrn, "Chart.Axes.Left.Minimum=0.0;")
pltscript(plotcbrn, "Chart.Axes.Left.Maximum=0.07;")
pltscript(plotcbrn, "Chart.Axes.Left.Title.Text=\"Remainder of the Brain [mg/L]\";")
pltscript(plotcbrn, "Chart.Axes.Bottom.Title.Text=\"Hours\";")
pltscript(plotcbrn, "Chart.Series[0].Title=\"Toluene\";")
pltscript(plotcbrn, "Chart.Series[1].Title=\"Ethylbenzene\";")
pltscript(plotcbrn, "Chart.Series[2].Title=\"Xylene\";")
pltscript(plotcbrn, "Chart.Series[3].Title=\"Nonane\";")
pltscript(plotcbrn, "Chart.Series[4].Title=\"Decane\";")
pltscript(plotcbrn, "Chart.Series[5].Title=\"Naphthalene\";")

pltscript(plotccoc, "Chart.Header.Text = \"Predictions for OEL Exposure - scaled\";")
pltscript(plotccoc, "Chart.SubHeader.Text = \"6 Key Hydrocarbons in JP-8\";")
pltscript(plotccoc, "Chart.SubHeader.Visible=true;")
pltscript(plotccoc, "Chart.Axes.Left.Automatic=false;")
pltscript(plotccoc, "Chart.Axes.Left.Minimum=0.0;")
pltscript(plotccoc, "Chart.Axes.Left.Maximum=0.07;")
pltscript(plotccoc, "Chart.Axes.Left.Title.Text=\"Cochlea [mg/L]\";")
pltscript(plotccoc, "Chart.Axes.Bottom.Title.Text=\"Hours\";")
pltscript(plotccoc, "Chart.Series[0].Title=\"Toluene\";")
pltscript(plotccoc, "Chart.Series[1].Title=\"Ethylbenzene\";")
pltscript(plotccoc, "Chart.Series[2].Title=\"Xylene\";")
pltscript(plotccoc, "Chart.Series[3].Title=\"Nonane\";")
pltscript(plotccoc, "Chart.Series[4].Title=\"Decane\";")
pltscript(plotccoc, "Chart.Series[5].Title=\"Naphthalene\";")

pltscript(plotcliv, "Chart.Header.Text = \"Predictions for OEL Exposure - scaled\";")
pltscript(plotcliv, "Chart.SubHeader.Text = \"6 Key Hydrocarbons in JP-8\";")
pltscript(plotcliv, "Chart.SubHeader.Visible=true;")
pltscript(plotcliv, "Chart.Axes.Left.Automatic=false;")
pltscript(plotcliv, "Chart.Axes.Left.Minimum=0.0;")
pltscript(plotcliv, "Chart.Axes.Left.Maximum=0.025;")
pltscript(plotcliv, "Chart.Axes.Left.Title.Text=\"Liver [mg/L]\";")
pltscript(plotcliv, "Chart.Axes.Bottom.Title.Text=\"Hours\";")
pltscript(plotcliv, "Chart.Series[0].Title=\"Toluene\";")
pltscript(plotcliv, "Chart.Series[1].Title=\"Ethylbenzene\";")
pltscript(plotcliv, "Chart.Series[2].Title=\"Xylene\";")
pltscript(plotcliv, "Chart.Series[3].Title=\"Nonane\";")
pltscript(plotcliv, "Chart.Series[4].Title=\"Decane\";")
pltscript(plotcliv, "Chart.Series[5].Title=\"Naphthalene\";")

pltscript(plotclng, "Chart.Header.Text = \"Predictions for OEL Exposure - scaled\";")
pltscript(plotclng, "Chart.SubHeader.Text = \"6 Key Hydrocarbons in JP-8\";")
pltscript(plotclng, "Chart.SubHeader.Visible=true;")
pltscript(plotclng, "Chart.Axes.Left.Automatic=false;")
pltscript(plotclng, "Chart.Axes.Left.Minimum=0.0;")
pltscript(plotclng, "Chart.Axes.Left.Maximum=0.025;")
pltscript(plotclng, "Chart.Axes.Left.Title.Text=\"Lung [mg/L]\";")
pltscript(plotclng, "Chart.Axes.Bottom.Title.Text=\"Hours\";")
pltscript(plotclng, "Chart.Series[0].Title=\"Toluene\";")
pltscript(plotclng, "Chart.Series[1].Title=\"Ethylbenzene\";")
pltscript(plotclng, "Chart.Series[2].Title=\"Xylene\";")
pltscript(plotclng, "Chart.Series[3].Title=\"Nonane\";")
pltscript(plotclng, "Chart.Series[4].Title=\"Decane\";")
pltscript(plotclng, "Chart.Series[5].Title=\"Naphthalene\";")

pltscript(plotcven, "Chart.Header.Text = \"Predictions for OEL Exposure - scaled\";")
pltscript(plotcven, "Chart.SubHeader.Text = \"6 Key Hydrocarbons in JP-8\";")
pltscript(plotcven, "Chart.SubHeader.Visible=true;")
pltscript(plotcven, "Chart.Axes.Left.Automatic=false;")
pltscript(plotcven, "Chart.Axes.Left.Minimum=0.0;")
pltscript(plotcven, "Chart.Axes.Left.Maximum=0.025;")
pltscript(plotcven, "Chart.Axes.Left.Title.Text=\"Venous Blood [mg/L]\";")
pltscript(plotcven, "Chart.Axes.Bottom.Title.Text=\"Hours\";")
pltscript(plotcven, "Chart.Series[0].Title=\"Toluene\";")
pltscript(plotcven, "Chart.Series[1].Title=\"Ethylbenzene\";")
pltscript(plotcven, "Chart.Series[2].Title=\"Xylene\";")
pltscript(plotcven, "Chart.Series[3].Title=\"Nonane\";")
pltscript(plotcven, "Chart.Series[4].Title=\"Decane\";")
```

```
    pltscript(plotcven, "Chart.Series[5].Title=\"Naphthalene\";")
set @preference=NoBackslashEscapes
```

Predict_RatOEL_Virent_50_50.m

```
% Simulates 6 key hydrocarbons at OEL concentration for JP-8 and Virent SAK 50:50 blend

  ResetDoses
  Rattus
  Rat_6HCs

% Calculates concentration of each component in POSF 4658 and 10326, converted from 200 mg/m3 to ppm, assum-
ing STP
% Weight percent values from tandem gas chromatography analysis by the 2006 Shafer et al. method
% POSF 4658 contains 0.16 percent toluene, 0.11 percent ethylbenzene,
%     0.67 percent xylenes, 1.14 percent nonane, 2.55 percent decane, 0.12% naphthalene
%     File name: JPC5 Fuels Comparison-Reanalysis Version.xlsx
% POSF 10326 contains 0.3 % toluene, 0.09% nonane, 0.13% decane, 0.02% naphthalene
%     10326 contains 0.67% C2-benzenes - divided by 4 then assigned 1 part to each component
%          ethylbenzene (0.17%), m-, o-, p-xylene (total = 0.05%)
%     File name: 10326-Virent SAK -GCxGC w C3-Alkylbenzene speciation.xlsx
% The 50-50 blend averages the concentrations of each compounent
% Simulates 8 hour work day
  BW=0.25; TCHNG=8.0; TSTOP=12.0;
  CONC(1)=((0.0023*200.0)*24.45)/MW(1);
  CONC(2)=((0.0014*200.0)*24.45)/MW(2);
  CONC(3)=((0.0059*200.0)*24.45)/MW(3);
  CONC(4)=((0.0062*200.0)*24.45)/MW(4);
  CONC(5)=((0.0134*200.0)*24.45)/MW(5);
  CONC(6)=((0.0007*200.0)*24.45)/MW(6);
  start @nocallback
  plotcbrnstm = plot (0, _t, _cbrnstm(:,1), '-b', _t, _cbrnstm(:,2),'-k', _t, _cbrnstm(:,3),'-r', _t,
_cbrnstm(:,4),'-g', _t, _cbrnstm(:,5),'-m', _t, _cbrnstm(:,6),'-c');
  plotcbrntl = plot (0, _t, _cbrntl(:,1), '-b', _t, _cbrntl(:,2),'-k', _t, _cbrntl(:,3),'-r', _t,
_cbrntl(:,4),'-g', _t, _cbrntl(:,5),'-m', _t, _cbrntl(:,6),'-c');
  plotcbrn = plot (0, _t, _cbrn(:,1), '-b', _t, _cbrn(:,2),'-k', _t, _cbrn(:,3),'-r', _t, _cbrn(:,4),'-g',
_t, _cbrn(:,5),'-m', _t, _cbrn(:,6),'-c');
  plotccoc = plot (0, _t, _ccoc(:,1), '-b', _t, _ccoc(:,2),'-k', _t, _ccoc(:,3),'-r', _t, _ccoc(:,4),'-g',
_t, _ccoc(:,5),'-m', _t, _ccoc(:,6),'-c');
  plotcliv = plot (0, _t, _cliv(:,1), '-b', _t, _cliv(:,2),'-k', _t, _cliv(:,3),'-r', _t, _cliv(:,4),'-g',
_t, _cliv(:,5),'-m', _t, _cliv(:,6),'-c');
  plotclng = plot (0, _t, _clng(:,1), '-b', _t, _clng(:,2),'-k', _t, _clng(:,3),'-r', _t, _clng(:,4),'-g',
_t, _clng(:,5),'-m', _t, _clng(:,6),'-c');
  plotcven = plot (0, _t, _cven(:,1), '-b', _t, _cven(:,2),'-k', _t, _cven(:,3),'-r', _t, _cven(:,4),'-g',
_t, _cven(:,5),'-m', _t, _cven(:,6),'-c');


set @preference=BackslashEscapes
  pltscript(plotcbrnstm, "Chart.Header.Text = \"Predictions for OEL Exposure - 50-50\";")
  pltscript(plotcbrnstm, "Chart.SubHeader.Text = \"6 Key Hydrocarbons in Virent SAK\";")
  pltscript(plotcbrnstm, "Chart.SubHeader.Visible=true;")
  pltscript(plotcbrnstm, "Chart.Axes.Left.Automatic=false;")
  pltscript(plotcbrnstm, "Chart.Axes.Left.Minimum=0.0;")
  pltscript(plotcbrnstm, "Chart.Axes.Left.Maximum=0.07;")
  pltscript(plotcbrnstm, "Chart.Axes.Left.Title.Text=\"Brain Stem [mg/L]\";")
  pltscript(plotcbrnstm, "Chart.Axes.Bottom.Title.Text=\"Hours\";")
  pltscript(plotcbrnstm, "Chart.Series[0].Title=\"Toluene\";")
  pltscript(plotcbrnstm, "Chart.Series[1].Title=\"Ethylbenzene\";")
  pltscript(plotcbrnstm, "Chart.Series[2].Title=\"Xylene\";")
  pltscript(plotcbrnstm, "Chart.Series[3].Title=\"Nonane\";")
  pltscript(plotcbrnstm, "Chart.Series[4].Title=\"Decane\";")
  pltscript(plotcbrnstm, "Chart.Series[5].Title=\"Naphthalene\";")

  pltscript(plotcbrntl, "Chart.Header.Text = \"Predictions for OEL Exposure - 50-50\";")
  pltscript(plotcbrntl, "Chart.SubHeader.Text = \"6 Key Hydrocarbons in Virent SAK\";")
  pltscript(plotcbrntl, "Chart.SubHeader.Visible=true;")
  pltscript(plotcbrntl, "Chart.Axes.Left.Automatic=false;")
  pltscript(plotcbrntl, "Chart.Axes.Left.Minimum=0.0;")
  pltscript(plotcbrntl, "Chart.Axes.Left.Maximum=0.07;")
  pltscript(plotcbrntl, "Chart.Axes.Left.Title.Text=\"Temporal Lobe [mg/L]\";")
  pltscript(plotcbrntl, "Chart.Axes.Bottom.Title.Text=\"Hours\";")
  pltscript(plotcbrntl, "Chart.Series[0].Title=\"Toluene\";")
  pltscript(plotcbrntl, "Chart.Series[1].Title=\"Ethylbenzene\";")
  pltscript(plotcbrntl, "Chart.Series[2].Title=\"Xylene\";")
```

```
pltscript(plotcbrntl, "Chart.Series[3].Title=\"Nonane\";")
pltscript(plotcbrntl, "Chart.Series[4].Title=\"Decane\";")
pltscript(plotcbrntl, "Chart.Series[5].Title=\"Naphthalene\";")

pltscript(plotcbrn, "Chart.Header.Text = \"Predictions for OEL Exposure - 50-50\";")
pltscript(plotcbrn, "Chart.SubHeader.Text = \"6 Key Hydrocarbons in Virent SAK\";")
pltscript(plotcbrn, "Chart.SubHeader.Visible=true;")
pltscript(plotcbrn, "Chart.Axes.Left.Automatic=false;")
pltscript(plotcbrn, "Chart.Axes.Left.Minimum=0.0;")
pltscript(plotcbrn, "Chart.Axes.Left.Maximum=0.07;")
pltscript(plotcbrn, "Chart.Axes.Left.Title.Text=\"Remainder of the Brain [mg/L]\";")
pltscript(plotcbrn, "Chart.Axes.Bottom.Title.Text=\"Hours\";")
pltscript(plotcbrn, "Chart.Series[0].Title=\"Toluene\";")
pltscript(plotcbrn, "Chart.Series[1].Title=\"Ethylbenzene\";")
pltscript(plotcbrn, "Chart.Series[2].Title=\"Xylene\";")
pltscript(plotcbrn, "Chart.Series[3].Title=\"Nonane\";")
pltscript(plotcbrn, "Chart.Series[4].Title=\"Decane\";")
pltscript(plotcbrn, "Chart.Series[5].Title=\"Naphthalene\";")

pltscript(plotccoc, "Chart.Header.Text = \"Predictions for OEL Exposure - 50-50\";")
pltscript(plotccoc, "Chart.SubHeader.Text = \"6 Key Hydrocarbons in Virent SAK\";")
pltscript(plotccoc, "Chart.SubHeader.Visible=true;")
pltscript(plotccoc, "Chart.Axes.Left.Automatic=false;")
pltscript(plotccoc, "Chart.Axes.Left.Minimum=0.0;")
pltscript(plotccoc, "Chart.Axes.Left.Maximum=0.07;")
pltscript(plotccoc, "Chart.Axes.Left.Title.Text=\"Cochlea [mg/L]\";")
pltscript(plotccoc, "Chart.Axes.Bottom.Title.Text=\"Hours\";")
pltscript(plotccoc, "Chart.Series[0].Title=\"Toluene\";")
pltscript(plotccoc, "Chart.Series[1].Title=\"Ethylbenzene\";")
pltscript(plotccoc, "Chart.Series[2].Title=\"Xylene\";")
pltscript(plotccoc, "Chart.Series[3].Title=\"Nonane\";")
pltscript(plotccoc, "Chart.Series[4].Title=\"Decane\";")
pltscript(plotccoc, "Chart.Series[5].Title=\"Naphthalene\";")

pltscript(plotcliv, "Chart.Header.Text = \"Predictions for OEL Exposure - 50-50\";")
pltscript(plotcliv, "Chart.SubHeader.Text = \"6 Key Hydrocarbons in Virent SAK\";")
pltscript(plotcliv, "Chart.SubHeader.Visible=true;")
pltscript(plotcliv, "Chart.Axes.Left.Automatic=false;")
pltscript(plotcliv, "Chart.Axes.Left.Minimum=0.0;")
pltscript(plotcliv, "Chart.Axes.Left.Maximum=0.025;")
pltscript(plotcliv, "Chart.Axes.Left.Title.Text=\"Liver [mg/L]\";")
pltscript(plotcliv, "Chart.Axes.Bottom.Title.Text=\"Hours\";")
pltscript(plotcliv, "Chart.Series[0].Title=\"Toluene\";")
pltscript(plotcliv, "Chart.Series[1].Title=\"Ethylbenzene\";")
pltscript(plotcliv, "Chart.Series[2].Title=\"Xylene\";")
pltscript(plotcliv, "Chart.Series[3].Title=\"Nonane\";")
pltscript(plotcliv, "Chart.Series[4].Title=\"Decane\";")
pltscript(plotcliv, "Chart.Series[5].Title=\"Naphthalene\";")

pltscript(plotclng, "Chart.Header.Text = \"Predictions for OEL Exposure - 50-50\";")
pltscript(plotclng, "Chart.SubHeader.Text = \"6 Key Hydrocarbons in Virent SAK\";")
pltscript(plotclng, "Chart.SubHeader.Visible=true;")
pltscript(plotclng, "Chart.Axes.Left.Automatic=false;")
pltscript(plotclng, "Chart.Axes.Left.Minimum=0.0;")
pltscript(plotclng, "Chart.Axes.Left.Maximum=0.025;")
pltscript(plotclng, "Chart.Axes.Left.Title.Text=\"Lung [mg/L]\";")
pltscript(plotclng, "Chart.Axes.Bottom.Title.Text=\"Hours\";")
pltscript(plotclng, "Chart.Series[0].Title=\"Toluene\";")
pltscript(plotclng, "Chart.Series[1].Title=\"Ethylbenzene\";")
pltscript(plotclng, "Chart.Series[2].Title=\"Xylene\";")
pltscript(plotclng, "Chart.Series[3].Title=\"Nonane\";")
pltscript(plotclng, "Chart.Series[4].Title=\"Decane\";")
pltscript(plotclng, "Chart.Series[5].Title=\"Naphthalene\";")

pltscript(plotcven, "Chart.Header.Text = \"Predictions for OEL Exposure - 50-50\";")
pltscript(plotcven, "Chart.SubHeader.Text = \"6 Key Hydrocarbons in Virent SAK\";")
pltscript(plotcven, "Chart.SubHeader.Visible=true;")
pltscript(plotcven, "Chart.Axes.Left.Automatic=false;")
pltscript(plotcven, "Chart.Axes.Left.Minimum=0.0;")
pltscript(plotcven, "Chart.Axes.Left.Maximum=0.025;")
pltscript(plotcven, "Chart.Axes.Left.Title.Text=\"Venous Blood [mg/L]\";")
pltscript(plotcven, "Chart.Axes.Bottom.Title.Text=\"Hours\";")
pltscript(plotcven, "Chart.Series[0].Title=\"Toluene\";")
pltscript(plotcven, "Chart.Series[1].Title=\"Ethylbenzene\";")
pltscript(plotcven, "Chart.Series[2].Title=\"Xylene\";")
pltscript(plotcven, "Chart.Series[3].Title=\"Nonane\";")
```

```
  pltscript(plotcven, "Chart.Series[4].Title=\"Decane\";")
  pltscript(plotcven, "Chart.Series[5].Title=\"Naphthalene\";")
set @preference=NoBackslashEscapes
```
