# Supplementary material for: Complex Mixtures: Array PBPK Modeling of Jet Fuel Components
Source: Toxics. 2023 Feb 17;11(2):187. doi: 10.3390/toxics11020187 (PMC9964161; doi:10.3390/toxics11020187)
Supplement: Supplementary file 1 [file toxics-11-00187-s001.zip › S2-Complex Mixtures Array Model Simulations.pdf]

toxics
MDPI

Supplementary Materials S2:

Complex Mixtures Array Model Simulations

Teresa R. Sterner [1,2,*], Tammie R. Covington [1,2] and David R. Mattie [2]

[1] Henry M. Jackson Foundation for the Advancement of Military Medicine; Wright-Patterson AFB, OH
[2] Air Force Research Laboratory, 711HPW/RHBAF; Wright-Patterson AFB, OH
* Correspondence: tsterner@hjfresearch.org

1. Simulations for Setting of Model Parameters

Validation simulations are simple visual comparisons between digitized published data and output from the array model. No statistical method was applied to determine differences. References are listed in the main article.

2. Toluene Simulations

Haddad *et al.* [46] data were used in part to set toluene parameters. Those simulations are shown in Figure 2 of the main article. Three additional data sets were used in parameterization (Figures S1 through S3).

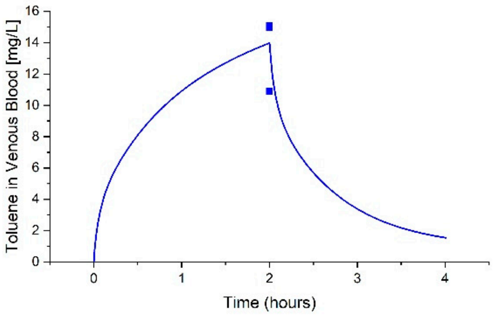

Figure S1. Toluene Parameter Setting Simulation using Lam *et al.* [61] Venous Blood Data. Male Sprague-Dawley (SD) rats were exposed to 488 ppm for 2 hours. The squares indicate individual rat data and the curve shows the array model simulation.

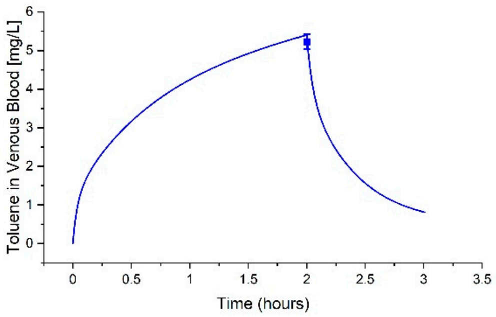

Figure S2. Toluene Parameter Setting Simulation using Romer *et al.* [55] Venous Blood Data. Female SD rats were exposed to 220 ppm for 2 hours. The data point and error bar indicate study mean ± standard error of the mean (SEM); the curve shows the array model simulation.

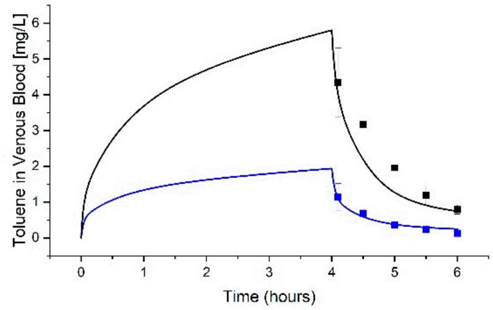

Figure S3. Toluene Parameter Setting Simulation using Tardif *et al.* [62] Venous Blood Data. Male SD rats were exposed to 100 ppm (blue lines and squares) or 200 ppm (black lines and squares) toluene for 4 hours. The data points and error bars indicate study means ± standard deviation (STDEV); the curves show the array model simulations.

3. Ethyl Benzene Simulations

Haddad *et al.* [46] data were used in part to set ethylbenzene parameters. Those simulations are shown in Figure 2 of the main article. One additional data set was used in parameterization (Figure S4).

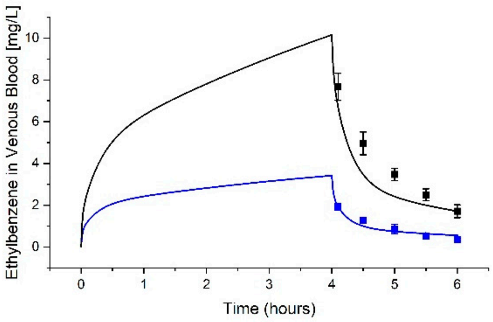

Figure S4. Ethylbenzene Parameter Setting Simulation using Tardif *et al.* [62] Venous Blood Data. Male SD rats were exposed to 100 ppm (blue lines and squares) or 200 ppm (black lines and squares) ethylbenzene for 4 hours. The data points and error bars indicate study means ± STDEV; the curves show the array model simulations.

4. Xylenes Simulations

Haddad *et al.* [46] data were used in part to set xylenes parameters. Those simulations are shown in Figure 2 of the main article. One additional data set was used in parameterization (Figure S5).

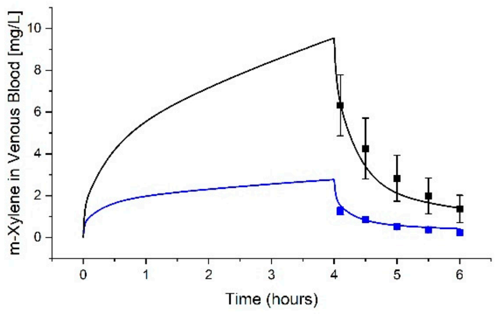

Figure S5. m-Xylene Parameter Setting Simulation using Tardif *et al.* [62] Venous Blood Data. Male SD rats were exposed to 100 ppm (blue lines and squares) or 200 ppm (black lines and squares) m-xylene for 4 hours. The data points and error bars indicate study means ± STDEV; the curves show the array model simulations.

5. n-Nonane Simulations

Lof *et al.* [45] data were used in part to set nonane parameters. Those simulations are shown in Figure 3 of the main article. Two additional data sets were used in parameterization (Figure S6).

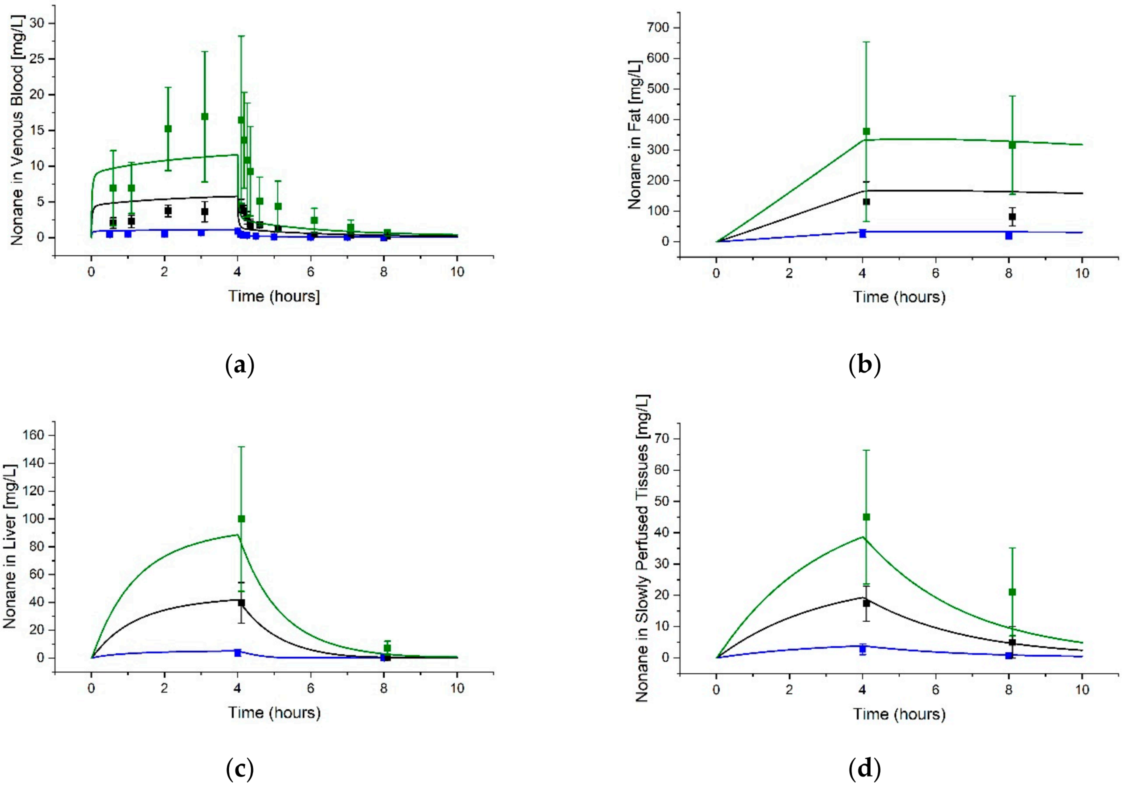

Figure S6. Nonane Parameter Setting Simulations using Robinson and Merrill [51] Data. Simulations depict (**a**) venous blood, (**b**) fat, (**c**) liver, and (**d**) slowly perfused (muscle) data. Female Fischer 344 (F344) rats were exposed to 100 ppm (blue lines and squares), 500 ppm (black lines and squares), or 1000 ppm (green lines and squares) nonane for 4 hours. The data points and error bars indicate study means ± STDEV; the curves show the array model simulations.

6. n-Decane Simulations

Lof *et al.* [45] data were used in part to set decane parameters. Those simulations are shown in Figure 3 of the main article. One additional data set was used in parameterization (Figure S7).

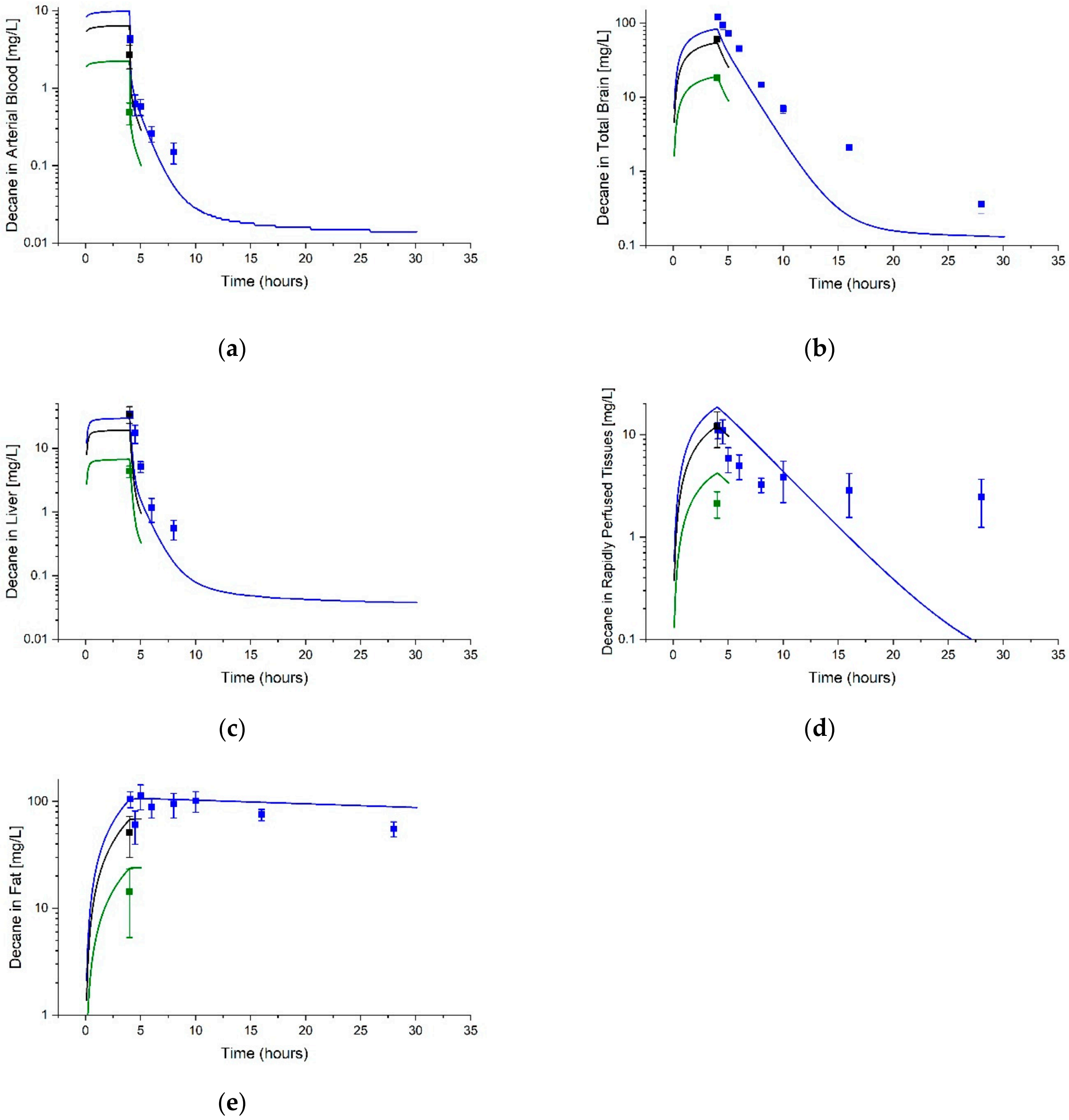

Figure S7. Decane Parameter Setting Simulations using Perleberg *et al.* [63] Data. Simulations depict (**a**) arterial blood, (**b**) total brain, (**c**) liver, (**d**) rapidly perfused (kidney), and (**e**) fat data. Male F344 rats were exposed to 273 ppm (green squares and lines), 781 ppm (black squares and lines), and 1200 ppm decane (blue squares and lines) for 12 hours a day for 3 days and follow-up continued for up to 24 hours post-exposure for the highest exposure group. The data points and error bars indicate study means ± STDEV; the curves show the array model simulations.

7. Naphthalene Simulations

NTP [50] single exposure data were used in part to set naphthalene parameters. Those simulations are shown in Figure 4 of the main article. Two additional data sets were used in parameterization (Figures S8 and S9). It should be noted that metabolic up-regulation was required to simulate the NTP [50] two-week exposure data in Figure S8. Liver metabolism was increased by 40% and lung metabolism was raised 12% for both male and female rats.

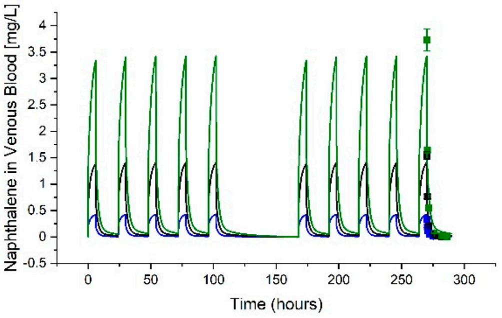

(**a**)

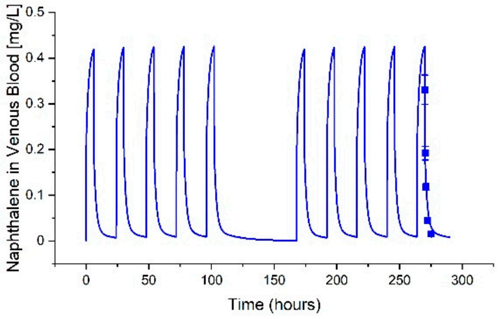

(**b**)

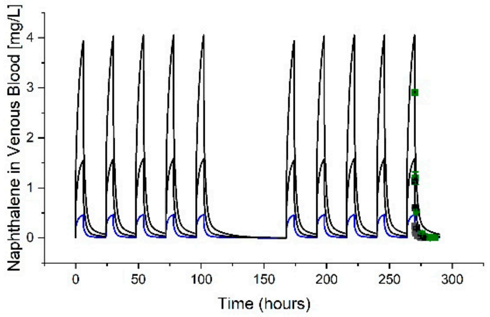

(**c**)

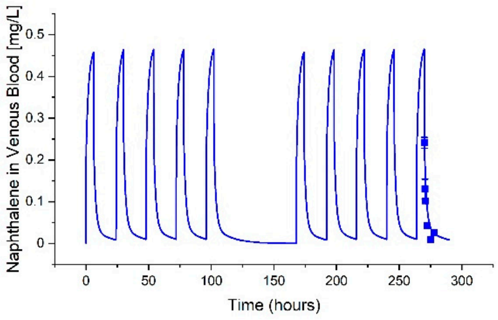

(**d**)

Figure S8. Naphthalene Parameter Setting Simulations using NTP [50] Repeated Exposure Data. (**a**) Simulations depict male rat venous concentrations from 10 ppm (blue squares and lines), 30 ppm (green squares and lines), and 60 ppm (pink squares and lines). (**b**) Closeup simulation for the male rat 10 ppm exposure only is shown. (**c**) Simulations depict female rat venous concentrations using the same color scheme. (**d**) Closeup simulation for the female rat 10 ppm exposure only is shown. F344 rats were exposed 8 hours a day, 5 days a week for 2 years; simulations were for the first 2 weeks of the study. The data points and error bars indicate study means ± SEM; the curves show the array model simulations.

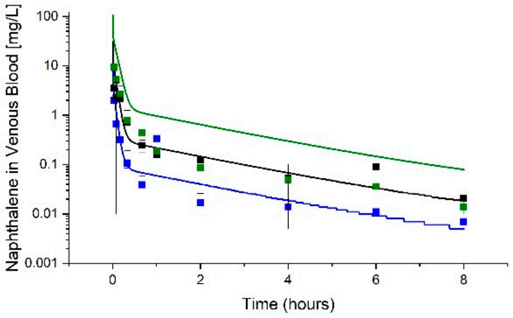

(**a**)

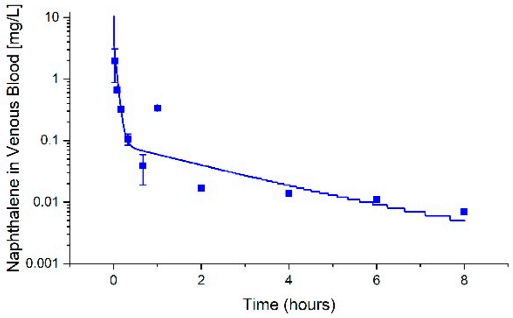

(**b**)

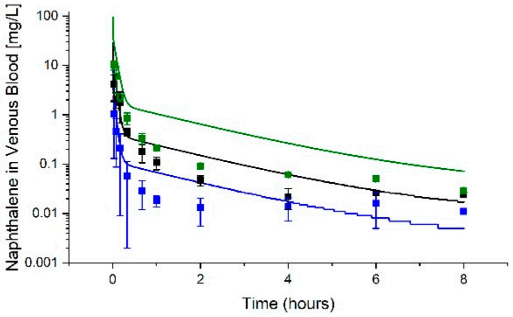

(**c**)

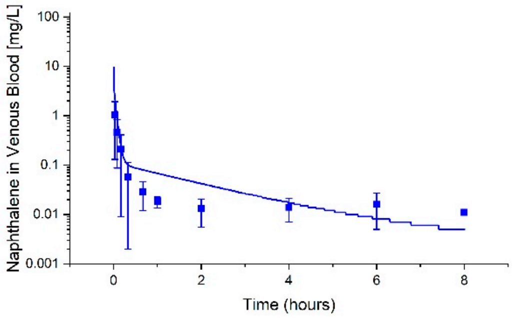

(**d**)

Figure S9. Naphthalene Parameter Setting Simulations using RTI [56] Intravenous Exposure Data. (**a**) Male F344 rat simulations depict venous concentrations from 1 mg/kg (blue squares and lines), 3 mg/kg (green squares and lines), and 10 mg/kg (pink squares and lines). (**b**) Closeup simulation for the male rat 1 mg/kg exposure only is shown. (**c**) Female F344 rat simulations depict venous concentrations from the same exposures as male rats. (**d**) Closeup simulation for the female rat 1 mg/kg exposure only is shown. The data points and error bars indicate study means ± SEM; the curves show the array model simulations.
